# A Concise Approach to *N*-Substituted Rhodanines through a Base-Assisted One-Pot Coupling and Cyclization Process

**DOI:** 10.3390/molecules25051138

**Published:** 2020-03-04

**Authors:** Yongxi Liang, Mei-Lin Tang, Zhipeng Huo, Chenchen Zhang, Xun Sun

**Affiliations:** 1 Department of Natural Medicine, School of Pharmacy, Fudan University, 826 Zhangheng Road, Shanghai 201203, China; 17111030010@fudan.edu.cn (Y.L.); 14111030017@fudan.edu.cn (M.-L.T.); 18111030010@fudan.edu.cn (Z.H.); 18211030009@fudan.edu.cn (C.Z.); 2 State Key Laboratory of Molecular Engineering and Institutes of Biomedical Sciences, Fudan University, 220 Handan Road, Shanghai 200433, China; 3 The Institutes of Integrative Medicine of Fudan University, 12 Wulumuqi Zhong Road, Shanghai 200040, China

**Keywords:** base, one-pot, coupling, cyclization, metal-free

## Abstract

An efficient approach to obtain functionalized rhodanines was developed through a base-assisted one-pot coupling and continuous cyclization of a primary amine, carbon disulfide, and methyl (2-chloroacetyl)carbamate. This conversion tolerates a broad range of functional groups and can be used to scale the preparation of *N*-substituted rhodanines in excellent yields.

## 1. Introduction

**Figure 1:**
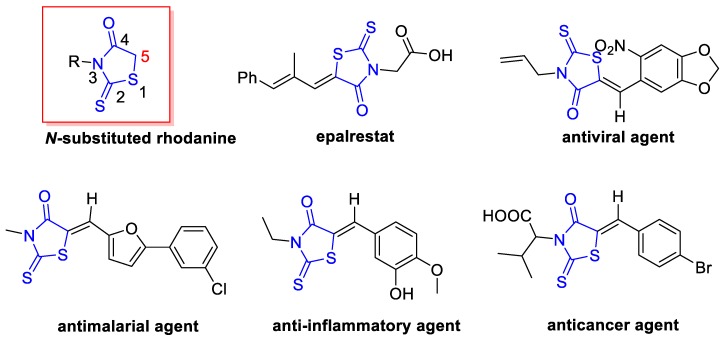
Selected pharmaceuticals and representative compounds bearing *N*-substituted rhodanines.

The exploration of effective approaches to access privileged structural motifs is one of the most important and urgent requirements in modern organic and pharmaceutical chemistry [1,2,3,4]. As a prime example, *N*-substituted rhodanines serve as versatile and useful subunits for numerous biological compounds, which are used in several pharmaceutical agents [5,6,7,8,9]. *N*-substituted rhodanines and their related heterocycles have been identified as synthetic building blocks and structural scaffolds which possess a unique biomolecular interaction profile [10,11,12,13,14]. However, as pan-assay interference compounds (PAINS), *N*-substituted rhodanines have been discovered in screening campaigns that have often been overinterpreted in the past [15]. Epalrestat, an aldose reductase inhibitor which has been used for the treatment of diabetic neuropathy in clinical practice, exemplifies the importance of these heterocycles [16,17]. Furthermore, *N*-substituted rhodanines have been demonstrated to have many biological activities, such as antiviral [18], antimalarial [19], anti-inflammatory [20], and anticancer activities (Figure 1) [21]. In addition, *N*-substituted rhodanines have been utilized in analytical chemistry to detect heavy metal ions [22,23]. Owing to the importance of *N*-substituted rhodanine scaffolds in pharmaceutical chemistry, synthesis methods have attracted a great deal of attention, and a number of powerful approaches have been reported. Even so, the development of an efficient and concise process to obtain *N*-substituted rhodanines is still a challenge in organic and pharmaceutical synthetic chemistry.

**Scheme 1:**
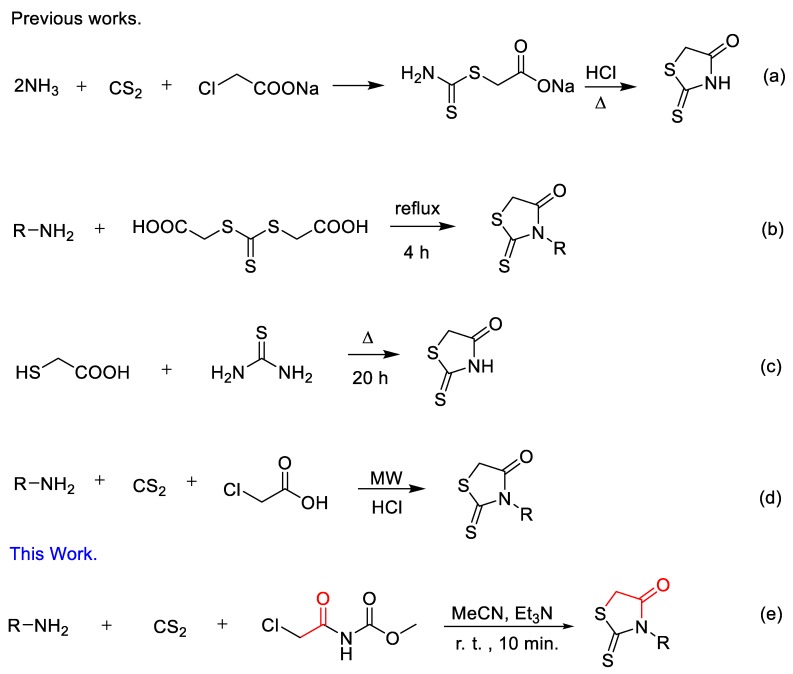
Synthesis of 5-unsubstituted rhodanines. (**1a**–**d**) previous works. (**1e**) our work.

Multicomponent processes are undoubtedly one of the most efficient approaches to forming several important motifs in modern organic synthesis and pharmaceutical synthesis [24,25,26]. For example, rhodanines have been successfully synthesized through multicomponent processes [27,28,29,30,31,32,33]. However, almost all known methods involving multicomponent reactions cannot achieve the synthesis of 5-unsubstituted rhodanines. Moreover, the known method for obtaining 5-unsubstituted rhodanines requires long reaction times and harsh reaction conditions (Scheme 1a−d) [32,34,35,36]. As a continuation of drug synthesis and interest in the synthetic methodology of *N*-substituted rhodanines, we decided to investigate the multicomponent reaction of a primary amine **1**, carbon disulphide **2**, and methyl (2-chloroacetyl)carbamate **3i** to form 5-unsubstituted rhodanines. To the best of our knowledge, no results through base-assisted one-pot coupling and a continuous cyclization process to obtain 5-unsubstituted rhodanines by the reactions of a primary amine, carbon disulfide, and methyl (2-chloroacetyl)carbamate have previously been reported (Scheme 1e).

## 2. Results and Discussion

### 2.1. Optimization of Reaction Conditions

Our investigation started with the three-component reaction of 4-Methylbenzylamine **1a**, carbon disulphide **2**, and 4-methoxyphenyl (2-chloroacetyl)carbamate **3a** (Table 1). First, when the mixture was treated with 1,8-diazabicyclo[5.4.0]undec-7-ene in MeCN, the desired product **4a** was produced in 74% yield (Table 1, Entry 1). Then, various bases, including *N*,*N*-diisopropylethylamine, Et_3_N, K_2_CO_3_, Na_2_CO_3_, K_3_PO_4_, and 4-dimethylaminopyridine, were screened, and the results are summarized in Table 1 (Entries 2−7). The results showed that most of these bases could promote the reaction, and Et_3_N was able to generate **4a** in 94% yield (Entry 3). Other solvents were also investigated, and the results showed that when MeCN was used, the yield of **4a** was up to 94% (Entries 8−17). It is worth mentioning that the base has a great influence on this process. When the reaction was treated in the absence of the base, the desired product **4a** did not form (Entry 18).

### 2.2. Optimization of Substrate Conditions

**Scheme 2:**
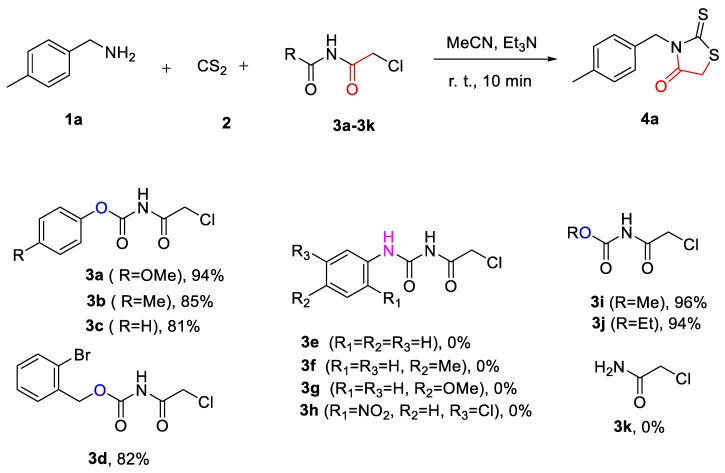
Optimization of substrate conditions. Reaction conditions: **1a** (0.5 mmol), **2** (0.5 mmol), **3a**–**3k** (0.5 mmol), Et_3_N (0.6 mmol), MeCN (3 mL), r. t., 10 min. Isolated yield based on **1a**.

To further investigate the limitations of this three-component system, various *N*-acetyl-2-chloroacetamides **3a**–**3k** were examined under the above-optimized reaction conditions (Scheme 2). When contacting esters **3a**–**3d** were used, the desired product **4a** was obtained in excellent yield. However, when contacting amides **3e**–**3h** were used, the reaction would not progress. In contrast, when methoxy group- (MeO-) and ethoxy group-(EtO-)substituted *N*-acetyl-2-chloroacetamides **3i** and **3j** were examined, an excellent yield of **4a** was obtained. However, 2-chloroacetamide **3k** could not promote this three-component reaction. Finally, as a low-cost and effective substrate, **3i,** was selected to investigate the scope of primary amines.

### 2.3. Substrate Scope Study

**Scheme 3:**
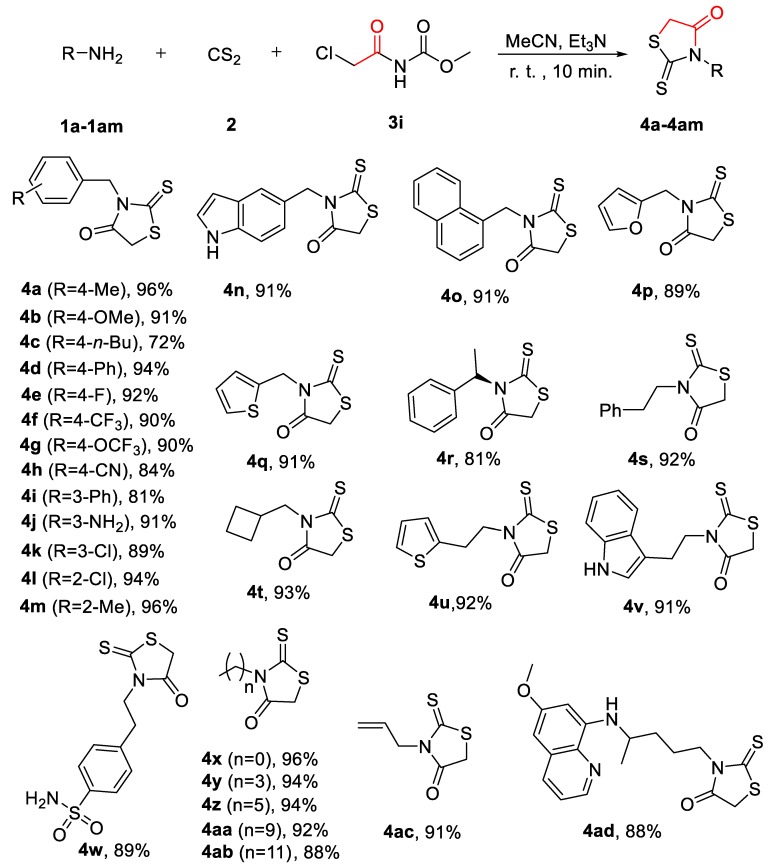

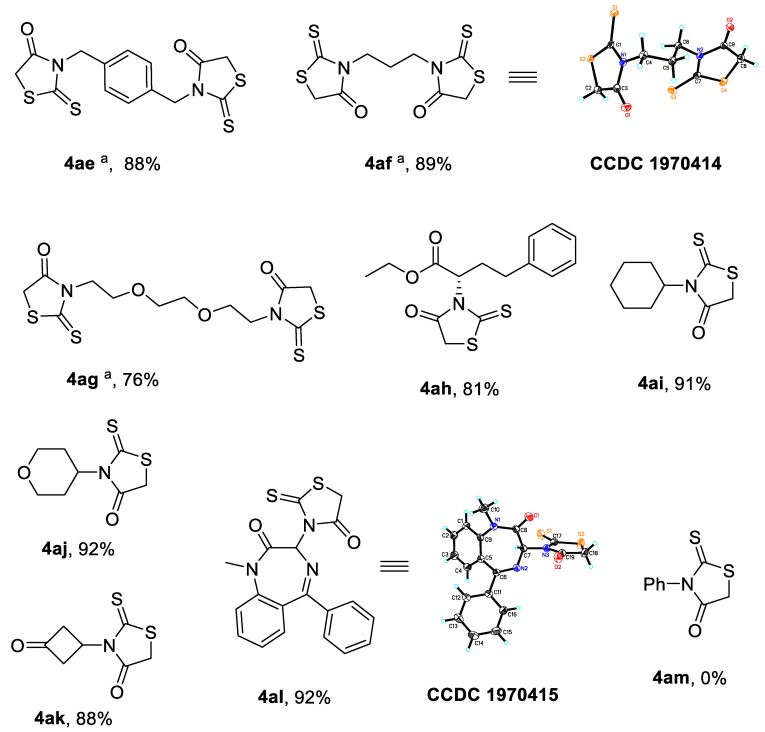
Scope of amines. Reaction conditions: **1a**–**1am** (0.5 mmol), **2** (0.5 mmol), **3i** (0.5 mmol), Et_3_N (0.6 mmol), MeCN (3 mL), r. t., 10 min. Isolated yield based on **1**. ^a^ **1** (0.25 mmol).

Next, we turned our attention to investigate the scope and limitations of three-component reactions of a primary amine **1**, carbon disulphide **2**, and methyl (2-chloroacetyl)carbamate **3i**. First, different substituted benzyl amines **1a**–**1m** were examined under the optimal conditions, as summarized in Scheme 3. In general, the three-component reactions with various substituted benzyl amines proceeded smoothly with excellent yields (**4a**–**4m**). Other benzyl-type amines **1n**–**1r** were also investigated in this process. The results showed that compounds **4n**–**4r** were also obtained in excellent yields. Various substituted ethylamines **1s**–**1w** were also examined, and the desired compounds, **4s**–**4w,** were successfully obtained in excellent yields. A series of linear primary amines **1x**–**1ad** were examined, and the desired 5-unsubstituted rhodanines **4x**–**4ad** were also generated in excellent yields. Notably, 1,4-phenylenedimethanamine, propane-1,3-diamine, and 2,2′-(ethane-1,2-diylbis(oxy))bis(ethan-1-amine) afforded **4ae**, **4af**, and **4ag** in 88%, 89%, and 76% yields, respectively. When several secondary amines, including 1ah and cyclic secondary amines **1ai**–**1al,** were investigated, the desired compounds, **4ah** and **4ai**–**4al,** were also produced in excellent yields. It is worth noting that when aniline was investigated, the desired compound, **4am,** was not obtained. The structures of 5-unsubstituted rhodanines **4af** (CCDC 1970414) and **4al** (CCDC 1970415) were unambiguously confirmed by X-ray crystallographic analysis**.**

### 2.4. Mechanism Study

**Scheme 4:**
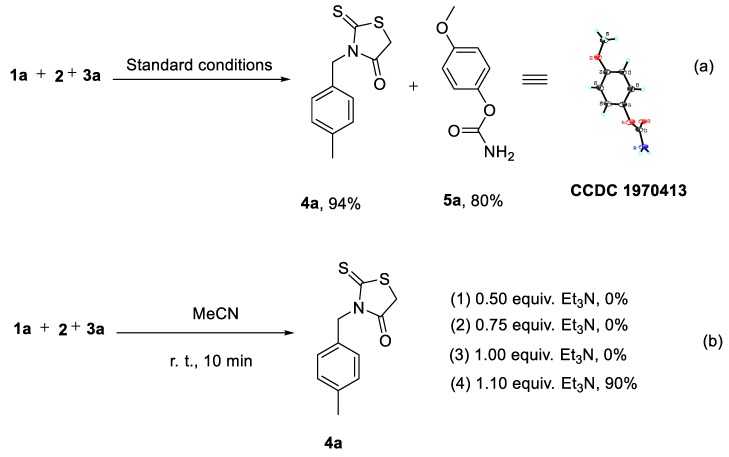
Mechanism study. (**a**) the model reaction. (**b**) comparison with different conditions in the model reaction.

To elucidate the mechanism, mechanistic studies were performed (Scheme 4). When **1a**, **2**, and **3a** were stirred under standard conditions in the absence of Et_3_N, the result showed that the reaction did not proceed (Table 1, Entry 18). Furthermore, when we scaled Et_3_N up to 0.50 or 0.75 equiv., the desired product **4a** was not yet formed. We found that activity returned after increasing from 1 equiv. to 1.10 equiv. Et_3_N (Scheme 4b, 90% yield). In the model reaction progress, we obtained the carbamate product, **5a,** in 80% yield (Scheme 4a). The structure of **5a** (CCDC 1970413) was confirmed by X-ray crystallographic analysis.

### 2.5. Plausible Mechanism

**Scheme 5:**
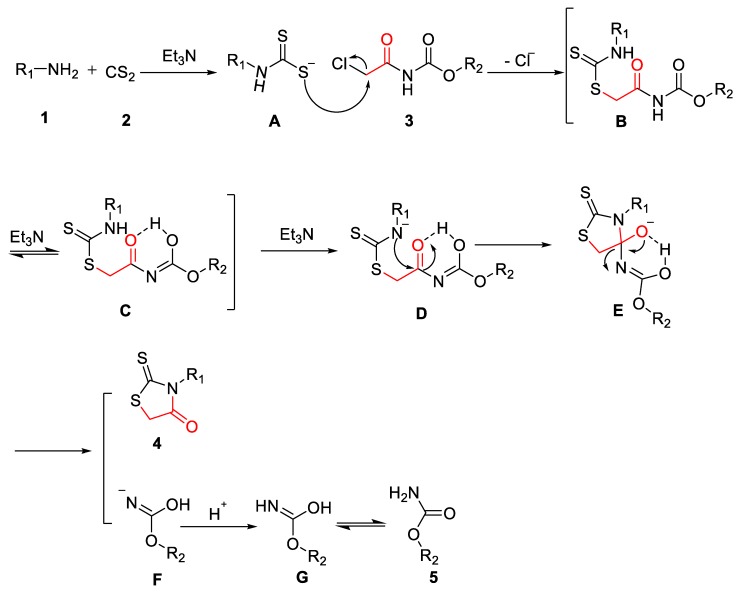
Plausible mechanism.

On the basis of the mechanistic studies and previous results [37,38], a possible mechanism is proposed in Scheme 5. The initial step in this reaction is the nucleophilic attack of amine **1** on carbon disulphide **2** to afford the key intermediate **A**, which subsequently reacts with **3** to form another intermediate, **B**, releasing 1 equiv. of protons from the reaction between **1** and **2**. Thus, at least 1 equiv. of Et_3_N is needed to scavenge this 1 equiv. of protons being released, and additional excess Et_3_N is then available to catalyze **B** to **D**. Considering the fact that the desired product **4a** was not obtained when replacing **3a**–**3d** (contacting ester) with **3e**–**3h** (contacting amide), the key lies in the difference in electronegativity and polarity between O atom and NH group. The O atom of the ester group does not affect the isomerization of imide, while NH group does, containing an active hydrogen atom, which may be the reason why the reaction of **3e**–**3h** could not progress. Therefore, it can be considered that **B** quickly undergoes isomerization to **C,** which, in turn, undergoes an intramolecular six-member ring cyclization and deprotonation to afford **D**. Then, the nucleophilic attack of thionamide on carbonyl produces the five-member ring intermediate **E**. Next, C–N bond cleavage of **E** generates 5-unsubstituted rhodanine **4** and intermediate **F**. Protonation and isomerization of **F** forms carbamate **5**.

## 3. Materials and Methods

### 3.1. General Information

All starting materials were commercially available and used without further purification unless otherwise stated. TLC analysis was performed using pre-coated glass plates. Column chromatography was performed using silica gel (200–300 mesh) for separation and purification. ^1^H NMR and ^19^F NMR spectra were recorded on Varian Mercury Plus400 spectrometers, and ^13^C NMR spectra was recorded on Bruker AVANCE600 spectrometer, with CDCl_3_ or DMSO-*d*_6_ as solvents. Resonances (*δ*) are given in parts per million relatives to tetramethylsilane or a residual solvent peak (CDCl_3_: ^1^H: *δ* = 7.26 ppm, ^13^C: *δ* = 77.00 ppm; DMSO-*d*_6_: ^1^H: *δ* = 2.50 ppm, ^13^C: *δ* = 39.50 ppm). Data are reported as follows: chemical shift; multiplicity (s = singlet, d = doublet, t = triplet, m = multiplet); coupling constants (Hz); and integration. HRMS were obtained on an AB Sciex Triple TOF^®^ 5600^+^. The X-ray crystal-structure determinations of **4af**, **4al,** and **5a** were obtained on a d8 venture system. Melting points were measured using a WRX-4 apparatus. Optical rotations were determined on a Rudolph Autopol IV polarimeter.

### 3.2. General Procedure A: Synthesis of 3a–3j

To a solution of 2-chloroacetamide (1.70 g, 18.2 mmol) in anhydrous 1,2-dichloroethane (20 mL) was added oxalyl chloride (2 mL) at 0 °C, then refluxed in an oil bath at 90 °C for 4 h. The reaction mixture was then cooled to 0 °C, and alcohol or amine (18.2 mmol) was added into the reaction mixture. The reaction mixture was stirred for another 5 min. Upon completion, the solid was filtrated and washed with 1,2-dichloroethane to give **3a**–**3j** [39].

**4-Methoxyphenyl (2-chloroacetyl)carbamate (3a):** 4-Methoxyphenol (2.26 g, 18.2 mmol) was used in general procedure A. The crude product was purified from the culture filtrate providing **3a** as a yellow solid in 78% yield (3.46 g, 14.2 mmol). M.p. 149.5–151.3 °C; ^1^H-NMR (400 MHz, DMSO-*d*_6_) *δ* 11.45 (s, 1H), 7.20–7.08 (m, 2H), 7.02–6.92 (m, 2H), 4.55 (s, 2H), 3.75 (s, 3H) ppm; ^13^C NMR (150 MHz, DMSO-*d*_6_) *δ* 166.9, 157.1, 150.5, 143.1, 122.6 (2C), 114.5, 114.5, 55.4, 44.3 ppm; HRMS (ESI): *m*/*z* [M+H]^+^ calcd for C_10_H_10_ClNO_4_: 244.0371, found: 244.0370.

***p*-Tolyl (2-chloroacetyl)carbamate (3b):***p*-Cresol (1.97 g, 18.2 mmol) was used in general procedure A. The crude product was purified from the culture filtrate providing **3b** as a yellow solid in 91% yield (3.77 g, 16.6 mmol). M.p. 162.8–164.2 °C; ^1^H-NMR (400 MHz, DMSO-*d*_6_) *δ* 11.46 (s, 1H), 7.30–7.16 (m, 2H), 7.13–7.00 (m, 2H), 4.55 (s, 2H), 2.31 (s, 3H) ppm; ^13^C NMR (150 MHz, DMSO-*d*_6_) *δ* 166.9, 150.2, 147.5, 135.4, 129.9 (2C), 121.4 (2C), 44.3, 20.4 ppm; HRMS (ESI): *m*/*z* [M+H]^+^ calcd for C_10_H_10_ClNO_3_: 228.0422, found: 228.0423.

**Phenyl (2-chloroacetyl)carbamate (3c):** Phenol (1.71 g, 18.2 mmol) was used in general procedure A. The crude product was purified from the culture filtrate providing **3c** as a white solid in 88% yield (3.42 g, 16.0 mmol). M.p. 130.1–132.0 °C; ^1^H-NMR (400 MHz, DMSO-*d*_6_) *δ* 11.51 (s, 1H), 7.49–7.40 (m, 2H), 7.33–7.26 (m, 1H), 7.25–7.18 (m, 2H), 4.56 (s, 2H) ppm; ^13^C NMR (150 MHz, DMSO-*d*_6_) *δ* 166.9, 150.1, 149.7, 129.6 (2C), 126.1, 121.7 (2C), 44.3 ppm; HRMS (ESI): *m*/*z* [M+H]^+^ calcd for C_9_H_8_ClNO_3_: 214.0265, found: 214.0266. NMR and HRMS data are consistent with those previously reported [40].

**2-Bromobenzyl (2-chloroacetyl)carbamate (3d):** (2-Bromophenyl)methanol (3.40 g, 18.2 mmol) was used in general procedure A. The crude product was purified from the culture filtrate providing **3d** as a white solid in 90% yield (5.02 g, 16.4 mmol). M.p. 154.7–156.3 °C; ^1^H-NMR (400 MHz, DMSO-*d*_6_) *δ* 11.18 (s, 1H), 7.74–7.62 (m, 1H), 7.60–7.51 (m, 1H), 7.49–7.39 (m, 1H), 7.37–7.26 (m, 1H), 5.20 (s, 2H), 4.49 (s, 2H) ppm; ^13^C NMR (150 MHz, DMSO-*d*_6_) *δ* 166.6, 151.2, 134.6, 132.6, 130.5, 130.4, 128.0, 122.8, 66.3, 44.2 ppm; HRMS (ESI): *m*/*z* [M-H]^−^ calcd for C_10_H_9_BrClNO_3_: 305.9360, found: 305.9350.

**2-Chloro-*N*-(phenylcarbamoyl)acetamide (3e):** Aniline (1.69 g, 18.2 mmol) was used in general procedure A. The crude product was purified from the culture filtrate providing **3e** as a white solid in 73% yield (2.83 g, 13.3 mmol). M.p. 150.4–152.1 °C; ^1^H-NMR (400 MHz, DMSO-*d*_6_) *δ* 10.92 (s, 1H), 10.17 (s, 1H), 7.62–7.45 (m, 2H), 7.40–7.32 (m, 2H), 7.18–7.04 (m, 1H), 4.40 (s, 2H) ppm; ^13^C NMR (150 MHz, DMSO-*d*_6_) *δ* 168.6, 150.2, 137.4, 128.9 (2C), 123.8, 119.7 (2C), 43.2 ppm; HRMS (ESI): *m*/*z* [M+H]^+^ calcd for C_9_H_9_ClN_2_O_2_: 213.0425, found: 213.0425. NMR and HRMS data are consistent with those previously reported [39].

**2-Chloro-*N*-(*p*-tolylcarbamoyl)acetamide (3f):***p*-Toluidine (1.95 g, 18.2 mmol) was used in general procedure A. The crude product was purified from the culture filtrate providing **3f** as a white solid in 71% yield (2.93 g, 12.9 mmol). M.p. 183.5–185.1 °C; ^1^H-NMR (400 MHz, DMSO-*d*_6_) *δ* 10.89 (s, 1H), 10.10 (s, 1H), 7.46–7.38 (m, 2H), 7.17–7.09 (m, 2H), 4.39 (s, 2H), 2.26 (s, 3H) ppm; ^13^C NMR (150 MHz, DMSO-*d*_6_) *δ* 168.6, 150.2, 134.9, 132.9, 129.3 (2C), 119.7 (2C), 43.2, 20.4 ppm; HRMS (ESI): *m*/*z* [M-H]^-^ calcd for C_10_H_11_ClN_2_O_2_: 225.0436, found: 225.0426. NMR and HRMS data are consistent with those previously reported [41].

**2-Chloro-*N*-((4-methoxyphenyl)carbamoyl)acetamide (3g):** 4-Methoxyaniline (2.24 g, 18.2 mmol) was used in general procedure A. The crude product was purified from the culture filtrate providing **3g** as a white solid in 70% yield (3.09 g, 12.7 mmol). M.p. 170.7–172.4 °C; ^1^H-NMR (400 MHz, DMSO-*d*_6_) *δ* 10.87 (s, 1H), 10.01 (s, 1H), 7.46–7.40 (m, 2H), 6.95–6.87 (m, 2H), 4.38 (s, 2H), 3.73 (s, 3H) ppm; ^13^C NMR (150 MHz, DMSO-*d*_6_) *δ* 168.5, 155.8, 150.2, 130.3, 121.6 (2C), 114.1 (2C), 55.2, 43.1 ppm; HRMS (ESI): *m*/*z* [M-H]^−^ calcd for C_10_H_11_ClN_2_O_3_: 241.0385, found: 241.0381. NMR and HRMS data are consistent with those previously reported [41].

**2-Chloro-*N*-((5-chloro-2-nitrophenyl)carbamoyl)acetamide (3h):** 5-Chloro-2-nitroaniline (3.14 g, 18.2 mmol) was used in general procedure A. The crude product was purified from the culture filtrate providing **3h** as a yellow solid in 42% yield (2.23 g, 7.6 mmol). M.p. 226.8–228.4 °C; ^1^H-NMR (400 MHz, DMSO-*d*_6_) *δ* 11.97 (s, 1H), 11.36 (s, 1H),8.61–8.53 (m, 1H), 8.25–8.17 (m, 1H), 7.42–7.35 (m, 1H), 4.41 (s, 2H) ppm; ^13^C NMR (150 MHz, DMSO-*d*_6_) *δ* 168.5, 150.4, 139.6, 136.6, 134.3, 127.5, 123.7, 122.1, 43.1 ppm; HRMS (ESI): *m*/*z* [M-H]^−^ calcd for C_9_H_7_Cl_2_N_3_O_4_: 289.9741, found: 289.9740.

**Methyl (2-chloroacetyl)carbamate (3i):** Methanol (0.58 g, 18.2 mmol) was used in general procedure A. The crude product was purified from the culture filtrate providing **3i** as a white solid in 95% yield (2.62 g, 17.3 mmol). M.p. 143.6–145.4 °C; ^1^H-NMR (400 MHz, DMSO-*d*_6_) *δ* 10.99 (s, 1H), 4.50 (s, 2H), 3.66 (s, 3H) ppm; ^13^C NMR (150 MHz, DMSO-*d*_6_) *δ* 166.7, 152.2, 52.5, 44.3 ppm; HRMS (ESI): *m*/*z* [M+H]^+^ calcd for C_4_H_6_ClNO_3_:152.0109, found: 152.0109. NMR and HRMS data are consistent with those previously reported [42].

**Ethyl (2-chloroacetyl)carbamate (3j):** Ethanol (0.84 g, 18.2 mmol) was used in general procedure A. The crude product was purified from the culture filtrate providing **3j** as a white solid in 95% yield (2.86 g, 17.3 mmol). M.p. 45.1–46.2 °C; ^1^H-NMR (400 MHz, DMSO-*d*_6_) *δ* 10.94 (s, 1H), 4.48 (s, 2H), 4.12 (q, *J* = 6.7 Hz, 2H), 1.21 (t, *J* = 7.0 Hz, 3H) ppm; ^13^C NMR (150 MHz, DMSO-*d*_6_) *δ* 166.7, 151.6, 61.4, 44.3, 14.1 ppm; HRMS (ESI): *m*/*z* [M+H]^+^ calcd for C_5_H_8_ClNO_3_: 166.0265, found: 166.0264.

### 3.3. General Procedure B: Synthesis of 4a-4al

A mixture of amine (0.5 mmol) or diamine (0.25 mmol), carbon disulfide (38 mg, 0.5 mmol), methyl (2-chloroacetyl)carbamate (76 mg, 0.5 mmol), and triethylamine (61 mg, 0.6 mmol) in MeCN (3 mL) was stirred at room temperature for 10 min. After disappearance of the reactant (monitored by TLC), added 50 mL water to the mixture, then extracted with ethyl acetate 3 times (3 × 50 mL). The extract was dried over anhydrous Na_2_SO_4_ and evaporated. The residue was purified by column chromatography on silica gel to give **4a**–**4al**.

**3-(4-Methylbenzyl)-2-thioxothiazolidin-4-one (4a):** Amine **1a** (61 mg, 0.5 mmol) was used in general procedure B. The crude product was purified by flash chromatography on silica gel providing **4a** as a red solid in 96% yield (114 mg, 0.48 mmol). TLC *R*_f_ = 0.24 (petroleum ether/ethyl acetate, 10:1); M.p. 71.2–72.7 °C; ^1^H-NMR (400 MHz, CDCl_3_) *δ* 7.37–7.31 (m, 2H), 7.15–7.09 (m, 2H), 5.15 (s, 2H), 3.96 (s, 2H), 2.32 (s, 3H) ppm; ^13^C NMR (150 MHz, CDCl_3_) *δ* 201.0, 173.9, 138.0, 131.7, 129.2 (2C), 129.1 (2C), 47.4, 35.4, 21.2 ppm; HRMS (ESI): *m*/*z* [M-H]^−^ calcd for C_11_H_11_NOS_2_: 236.0209, found: 236.0208. NMR and HRMS data are consistent with those previously reported [33].

**3-(4-Methoxybenzyl)-2-thioxothiazolidin-4-one (4b):** Amine **1b** (69 mg, 0.5 mmol) was used in general procedure B. The crude product was purified by flash chromatography on silica gel providing **4b** as a red solid in 91% yield (115 mg, 0.46 mmol). TLC *R*_f_ = 0.23 (petroleum ether/ethyl acetate, 10:1); M.p. 98.1–99.3 °C; ^1^H-NMR (400 MHz, CDCl_3_) *δ* 7.47–7.38 (m, 2H), 6.87–6.79 (m, 2H), 5.12 (s, 2H), 3.95 (s, 2H), 3.78 (s, 3H) ppm; ^13^C NMR (150 MHz, CDCl_3_) *δ* 201.1, 173.9, 159.5, 130.8 (2C), 127.0, 113.8 (2C), 55.3, 47.1, 35.4 ppm; HRMS (ESI): *m*/*z* [M-H]^−^ calcd for C_11_H_11_NO_2_S_2_: 252.0158, found: 252.0158. NMR and HRMS data are consistent with those previously reported [33].

**3-(4-Butylbenzyl)-2-thioxothiazolidin-4-one (4c):** Amine **1c** (82 mg, 0.5 mmol) was used in general procedure B. The crude product was purified by flash chromatography on silica gel providing **4c** as a red oil in 72% yield (101 mg, 0.36 mmol). TLC *R*_f_ = 0.24 (petroleum ether/ethyl acetate, 10:1); ^1^H-NMR (400 MHz, CDCl_3_) *δ* 7.41–7.33 (m, 2H), 7.16–7.08 (m, 2H), 5.15 (d, *J* = 2.5 Hz, 2H), 3.97 (d, *J* = 2.9 Hz, 2H), 2.59–2.55 (m, 2H), 1.63–1.51 (m, 2H), 1.38–1.29 (m, 2H), 0.97–0.88 (m, 3H) ppm; ^13^C NMR (150 MHz, CDCl_3_) *δ* 201.1, 173.9, 143.1, 131.9, 129.1 (2C), 128.6 (2C), 47.4, 35.4, 35.3, 33.5, 22.3, 13.9 ppm; HRMS (ESI): *m*/*z* [M-H]^−^ calcd for C_14_H_17_NOS_2_: 278.0679, found: 278.0675.

**3-([1,1′-Biphenyl]-4-ylmethyl)-2-thioxothiazolidin-4-one (4d):** Amine **1d** (92 mg, 0.5 mmol) was used in general procedure B. The crude product was purified by flash chromatography on silica gel providing **4d** as a white solid in 94% yield (141 mg, 0.47 mmol). TLC *R*_f_ = 0.21 (petroleum ether/ethyl acetate, 10:1); M.p. 130.2–131.9 °C; ^1^H-NMR (400 MHz, CDCl_3_) *δ* 7.61–7.49 (m, 6H), 7.47–7.41 (m, 2H), 7.39–7.32 (m, 1H), 5.23 (s, 2H), 4.00 (s, 2H) ppm; ^13^C NMR (150 MHz, CDCl_3_) *δ* 201.0, 173.9, 141.2, 140.6, 133.7, 129.6 (2C), 128.8 (2C), 127.5, 127.3 (2C), 127.1 (2C), 47.3, 35.4 ppm; HRMS (ESI): *m*/*z* [M-H]^−^ calcd for C_16_H_13_NOS_2_: 298.0366, found: 298.0367.

**3-(4-Fluorobenzyl)-2-thioxothiazolidin-4-one (4e):** Amine **1e** (63 mg, 0.5 mmol) was used in general procedure B. The crude product was purified by flash chromatography on silica gel providing **4e** as a brown oil in 92% yield (111 mg, 0.46 mmol). TLC *R*_f_ = 0.29 (petroleum ether/ethyl acetate, 10:1); ^1^H-NMR (400 MHz, CDCl_3_) *δ* 7.61–7.52 (m, 4H), 5.23 (s, 2H), 4.02 (s, 2H) ppm; ^13^C NMR (150 MHz, CDCl_3_) *δ* 200.8, 173.7, 138.4, 129.3 (3C), 125.6, 125.6, 47.0, 35.4 ppm; ^19^F NMR (376 MHz, CDCl_3_) *δ* −113.6 ppm; HRMS (ESI): *m*/*z* [M-H]^−^ calcd for C_10_H_8_FNOS_2_: 239.9959, found: 239.9958. NMR and HRMS data are consistent with those previously reported [34].

**2-Thioxo-3-(4-(trifluoromethyl)benzyl)thiazolidin-4-one (4f):** Amine **1f** (88 mg, 0.5 mmol) was used in general procedure B. The crude product was purified by flash chromatography on silica gel providing **4f** as a brown solid in 90% yield (131 mg, 0.45 mmol). TLC *R*_f_ = 0.31 (petroleum ether/ethyl acetate, 10:1); M.p. 124.0–125.5 °C; ^1^H-NMR (400 MHz, CDCl_3_) *δ* 7.49–7.42 (m, 2H), 7.03–6.95 (m, 2H), 5.14 (s, 2H), 3.98 (s, 2H) ppm; ^13^C NMR (150 MHz, CDCl_3_) *δ* 200.9, 173.8, 163.3, 161.7, 131.2, 131.1, 130.5, 115.5, 115.3, 46.8, 35.4 ppm; ^19^F NMR (376 MHz, CDCl_3_) *δ* −62.9 ppm; HRMS (ESI): *m*/*z* [M-H]^−^ calcd for C_11_H_8_F_3_NOS_2_: 289.9927, found: 289.9928. NMR and HRMS data are consistent with those previously reported [43].

**2-Thioxo-3-(4-(trifluoromethoxy)benzyl)thiazolidin-4-one (4g):** Amine **1g** (96 mg, 0.5 mmol) was used in general procedure B. The crude product was purified by flash chromatography on silica gel providing **4g** as a brown oil in 90% yield (138 mg, 0.45 mmol). TLC *R*_f_ = 0.24 (petroleum ether/ethyl acetate, 10:1); ^1^H-NMR (400 MHz, CDCl_3_) *δ* 7.57–7.46 (m, 2H), 7.20–7.12 (m, 2H), 5.17 (s, 2H), 4.00 (s, 2H) ppm; ^13^C NMR (150 MHz, CDCl_3_) *δ* 200.9, 173.8, 149.1, 133.3, 130.8 (2C), 121.0 (2C), 119.5, 46.8, 35.4 ppm; ^19^F NMR (376 MHz, CDCl_3_) *δ* −58.0 ppm; HRMS (ESI): *m*/*z* [M-H]^−^ calcd for C_11_H_8_F_3_NO_2_S_2_: 305.9876, found: 305.9875.

**4-((4-Oxo-2-thioxothiazolidin-3-yl)methyl)benzonitrile (4h):** Amine **1h** (66 mg, 0.5 mmol) was used in general procedure B. The crude product was purified by flash chromatography on silica gel providing **4h** as a yellow solid in 84% yield (104 mg, 0.42 mmol). TLC *R*_f_ = 0.29 (petroleum ether/ethyl acetate, 6:1); M.p. 158.0–159.8 °C; ^1^H-NMR (400 MHz, CDCl_3_) *δ* 7.53–7.47 (m, 2H), 7.19–7.13 (m, 2H), 5.21 (s, 2H), 4.04 (s, 2H) ppm; ^13^C NMR (150 MHz, CDCl_3_) *δ* 200.7, 173.7, 139.7, 132.4 (2C), 129.6 (2C), 118.4, 112.1, 47.0, 35.5 ppm; HRMS (ESI): *m*/*z* [M-H]^−^ calcd for C_11_H_8_N_2_OS_2_: 247.0005, found: 247.0007. NMR and HRMS data are consistent with those previously reported [44].

**3-([1,1′-Biphenyl]-3-ylmethyl)-2-thioxothiazolidin-4-one (4i):** Amine **1i** (92 mg, 0.5 mmol) was used in general procedure B. The crude product was purified by flash chromatography on silica gel providing **4i** as a red oil in 81% yield (121 mg, 0.41 mmol). TLC *R*_f_ = 0.30 (petroleum ether/ethyl acetate, 10:1); ^1^H-NMR (400 MHz, CDCl_3_) *δ* 7.71–7.67 (m, 1H), 7.60–7.51 (m, 3H), 7.50–7.32 (m, 5H), 5.25 (s, 2H), 3.98 (s, 2H) ppm; ^13^C NMR (150 MHz, CDCl_3_) *δ* 201.0, 173.9, 141.6, 140.6, 135.2, 129.0, 128.8 (2C), 128.0 (2C), 127.5, 127.2 (2C), 127.0, 47.6, 35.4 ppm; HRMS (ESI): *m*/*z* [M-H]^−^ calcd for C_16_H_13_NOS_2_: 298.0366, found: 298.0368.

**3-(3-Aminobenzyl)-2-thioxothiazolidin-4-one (4j):** Amine **1j** (61 mg, 0.5 mmol) was used in general procedure B. The crude product was purified by flash chromatography on silica gel providing **4j** as a yellow oil in 91% yield (108 mg, 0.46 mmol). TLC *R*_f_ = 0.21 (petroleum ether/ethyl acetate, 3:1); ^1^H-NMR (400 MHz, CDCl_3_) *δ* 7.14–7.05 (m, 1H), 6.85–6.79 (m, 1H), 6.75 (s, 1H), 6.64–6.57 (m, 1H), 5.09 (s, 2H), 3.97 (s, 2H), 3.30 (s, 2H) ppm; ^13^C NMR (150 MHz, CDCl_3_) *δ* 201.1, 173.9, 146.6, 135.8, 129.5, 119.2, 115.3, 114.9, 47.6, 35.4 ppm; HRMS (ESI): *m*/*z* [M-H]^−^ calcd for C_10_H_10_N_2_OS_2_: 237.0162, found: 237.0156.

**3-(3-Chlorobenzyl)-2-thioxothiazolidin-4-one (4k):** Amine **1k** (71 mg, 0.5 mmol) was used in general procedure B. The crude product was purified by flash chromatography on silica gel providing **4k** as a brown oil in 89% yield (115 mg, 0.45 mmol). TLC *R*_f_ = 0.30 (petroleum ether/ethyl acetate, 10:1); ^1^H-NMR (400 MHz, CDCl_3_) *δ* 7.41 (s, 1H), 7.36–7.30 (m, 1H), 7.29–7.22 (m, 2H), 5.14 (s, 2H), 4.01 (s, 2H) ppm; ^13^C NMR (150 MHz, CDCl_3_) *δ* 200.8, 173.7, 136.5, 134.4, 129.8, 129.0, 128.4, 127.3, 47.0, 35.4 ppm; HRMS (ESI): *m*/*z* [M-H]^−^ calcd for C_10_H_8_ClNOS_2_: 255.9663, found: 255.9663.

**3-(2-Chlorobenzyl)-2-thioxothiazolidin-4-one (4l):** Amine **1l** (71 mg, 0.5 mmol) was used in general procedure B. The crude product was purified by flash chromatography on silica gel providing **4l** as a yellow solid in 94% yield (121 mg, 0.47 mmol). TLC *R*_f_ = 0.25 (petroleum ether/ethyl acetate, 8:1); M.p. 73.4–74.6 °C; ^1^H-NMR (400 MHz, CDCl_3_) *δ* 7.41–7.36 (m, 1H), 7.25–7.15 (m, 2H), 6.95-6.87 (m, 1H), 5.29 (s, 2H), 4.10 (s, 2H) ppm; ^13^C NMR (150 MHz, CDCl_3_) *δ* 200.5, 173.5, 132.9, 131.6, 129.8, 128.8, 126.9, 126.9, 45.4, 35.5 ppm; HRMS (ESI): *m*/*z* [M-H]^−^ calcd for C_10_H_8_ClNOS_2_: 255.9663, found: 255.9663. NMR and HRMS data are consistent with those previously reported [33].

**3-(2-Methylbenzyl)-2-thioxothiazolidin-4-one (4m):** Amine **1m** (61 mg, 0.5 mmol) was used in general procedure B. The crude product was purified by flash chromatography on silica gel providing **4m** as a white solid in 96% yield (114 mg, 0.48 mmol). TLC *R*_f_ = 0.31 (petroleum ether/ethyl acetate, 8:1); M.p. 107.0–108.4 °C; ^1^H-NMR (400 MHz, CDCl_3_) *δ* 7.21–7.15 (m, 2H), 7.15–7.06 (m, 1H), 6.95–6.87 (m, 1H), 5.17 (s, 2H), 4.06 (s, 2H), 2.43 (s, 3H) ppm; ^13^C NMR (150 MHz, CDCl_3_) *δ* 200.9, 173.8, 135.7, 132.2, 130.5, 127.6, 126.1, 125.5, 45.2, 35.5, 19.5 ppm; HRMS (ESI): *m*/*z* [M-H]^−^ calcd for C_11_H_11_NOS_2_: 236.0209, found: 236.0208.

**3-((1*H*-Indol-5-yl)methyl)-2-thioxothiazolidin-4-one (4n):** Amine **1n** (73 mg, 0.5 mmol) was used in general procedure B. The crude product was purified by flash chromatography on silica gel providing **4n** as a white solid in 91% yield (119 mg, 0.46 mmol). TLC *R*_f_ = 0.22 (petroleum ether/ethyl acetate, 3:1); M.p. 157.8–159.2 °C; ^1^H-NMR (400 MHz, DMSO-*d*_6_) *δ* 11.11 (s, 1H), 7.54–7.46 (m, 1H), 7.39–7.29 (m, 2H), 7.15–7.06 (m, 1H), 6.43–6.35 (m, 1H), 5.13 (s, 2H), 4.34 (s, 2H) ppm; ^13^C NMR (150 MHz, DMSO-*d*_6_) *δ* 203.2, 174.6, 135.3, 127.4, 125.9, 125.4, 121.4, 119.8, 111.2, 101.0, 47.5, 35.9 ppm; HRMS (ESI): *m*/*z* [M-H]^−^ calcd for C_12_H_10_N_2_OS_2_: 261.0162, found: 261.0161.

**3-(Naphthalen-1-ylmethyl)-2-thioxothiazolidin-4-one (4o):** Amine **1o** (79 mg, 0.5 mmol) was used in general procedure B. The crude product was purified by flash chromatography on silica gel providing **4o** as a yellow oil in 91% yield (124 mg, 0.46 mmol). TLC *R*_f_ = 0.29 (petroleum ether/ethyl acetate, 10:1); ^1^H-NMR (400 MHz, CDCl_3_) *δ* 8.15–8.08 (m, 1H), 7.91–7.84 (m, 1H), 7.81–7.74 (m, 1H), 7.63–7.47 (m, 2H), 7.42–7.33 (m, 1H), 7.17–7.09 (m, 1H), 5.66 (s, 2H), 4.05 (s, 2H) ppm; ^13^C NMR (150 MHz, CDCl_3_) *δ* 200.9, 173.7, 133.7, 130.8, 129.2, 128.9, 128.4, 126.5, 125.9, 125.1, 123.9, 122.9, 45.4, 35.4 ppm; HRMS (ESI): *m*/*z* [M-H]^−^ calcd for C_14_H_11_NOS_2_: 272.0209, found: 272.0206.

**3-(Furan-2-ylmethyl)-2-thioxothiazolidin-4-one (4p):** Amine **1p** (49 mg, 0.5 mmol) was used in general procedure B. The crude product was purified by flash chromatography on silica gel providing **4p** as a red oil in 89% yield (95 mg, 0.45 mmol). TLC *R*_f_ = 0.28 (petroleum ether/ethyl acetate, 10:1); ^1^H-NMR (400 MHz, CDCl_3_) *δ* 7.41–7.31 (m, 1H), 6.48–6.38 (m, 1H), 6.36–7.26 (m, 1H), 5.19 (s, 2H), 4.01 (s, 2H) ppm; ^13^C NMR (150 MHz, CDCl_3_) *δ* 200.3, 173.4, 147.7, 142.6, 110.5, 110.5, 40.4, 35.3 ppm; HRMS (ESI): *m*/*z* [M-H]^−^ calcd for C_8_H_7_NO_2_S_2_: 211.9845, found: 211.9845. NMR and HRMS data are consistent with those previously reported [45].

**3-(Thiophen-2-ylmethyl)-2-thioxothiazolidin-4-one (4q):** Amine **1q** (57 mg, 0.5 mmol) was used in general procedure B. The crude product was purified by flash chromatography on silica gel providing **4q** as a red solid in 91% yield (104 mg, 0.46 mmol). TLC *R*_f_ = 0.22 (petroleum ether/ethyl acetate, 10:1); M.p. 93.7–95.4 °C; ^1^H-NMR (400 MHz, CDCl_3_) *δ* 7.34–7.17 (m, 2H), 7.03–6.89 (m, 1H), 5.33 (s, 2H), 3.97 (s, 2H) ppm; ^13^C NMR (150 MHz, CDCl_3_) *δ* 200.2, 173.3, 135.8, 129.2, 126.6, 126.4, 41.9, 35.4 ppm; HRMS (ESI): *m*/*z* [M-H]^−^ calcd for C_8_H_7_NOS_3_: 227.9617, found: 227.9619. NMR and HRMS data are consistent with those previously reported [45].

**(*S*)-3-(1-Phenylethyl)-2-thioxothiazolidin-4-one (4r):** Amine **1r** (61 mg, 0.5 mmol) was used in general procedure B. The crude product was purified by flash chromatography on silica gel providing **4r** as a brown oil in 81% yield (96 mg, 0.41 mmol). TLC *R*_f_ = 0.30 (petroleum ether/ethyl acetate, 10:1); [α]_D_^25^ = 26.2 (*c* 1.00, CH_3_OH); ^1^H-NMR (400 MHz, CDCl_3_) *δ* 7.38–7.27 (m, 2H), 7.50–7.40 (m, 3H), 6.39 (q, *J* = 7.2 Hz, 1H), 3.79 (q, 2H), 1.86 (d, 3H) ppm; ^13^C NMR (150 MHz, CDCl_3_) *δ* 202.1, 173.4, 138.0, 128.3 (2C), 127.9, 127.6 (2C), 55.2, 34.1, 15.0 ppm; HRMS (ESI): *m*/*z* [M-H]^−^ calcd for C_11_H_11_NOS_2_: 236.0209, found: 236.0207. NMR and HRMS data are consistent with those previously reported [46].

**3-Phenethyl-2-thioxothiazolidin-4-one (4s):** Amine **1s** (61 mg, 0.5 mmol) was used in general procedure B. The crude product was purified by flash chromatography on silica gel providing **4s** as a red solid in 92% yield (109 mg, 0.46 mmol). TLC *R*_f_ = 0.24 (petroleum ether/ethyl acetate, 10:1); M.p. 111.3–112.7 °C; ^1^H-NMR (400 MHz, CDCl_3_) *δ* 7.45–7.15 (m, 5H), 4.25–4.15 (m, 2H), 3.93 (s, 2H), 3.03–2.89 (m, 2H) ppm; ^13^C NMR (150 MHz, CDCl_3_) *δ* 200.9, 173.5, 137.3, 128.9 (2C), 128.6 (2C), 126.8, 45.7, 35.2, 32.6 ppm; HRMS (ESI): *m*/*z* [M-H]^−^ calcd for C_11_H_11_NOS_2_: 236.0209, found: 236.0211. NMR and HRMS data are consistent with those previously reported [34].

**3-(Cyclobutylmethyl)-2-thioxothiazolidin-4-one (4t):** Amine **1t** (43 mg, 0.5 mmol) was used in general procedure B. The crude product was purified by flash chromatography on silica gel providing **4t** as a yellow oil in 93% yield (94 mg, 0.47 mmol). TLC *R*_f_ = 0.21 (petroleum ether/ethyl acetate, 10:1); ^1^H-NMR (400 MHz, CDCl_3_) *δ* 4.06 (dd, *J* = 7.3, 1.6 Hz, 2H), 3.97 (d, *J* = 1.7 Hz, 2H), 2.80 (dd, *J* = 14.7, 7.3 Hz, 1H), 2.09–1.92 (m, 2H), 1.75–1.91 (m, 4H) ppm; ^13^C NMR (150 MHz, CDCl_3_) *δ* 201.6, 174.2, 49.4, 35.3, 33.4, 26.2 (2C), 18.3 ppm; HRMS (ESI): *m*/*z* [M-H]^−^ calcd for C_8_H_11_NOS_2_: 200.0209, found: 200.0208.

**3-(2-(Thiophen-2-yl)ethyl)-2-thioxothiazolidin-4-one (4u):** Amine **1u** (64 mg, 0.5 mmol) was used in general procedure B. The crude product was purified by flash chromatography on silica gel providing **4u** as a brown solid in 92% yield (112 mg, 0.46 mmol). TLC *R*_f_ = 0.30 (petroleum ether/ethyl acetate, 10:1); M.p. 125.5–127.3 °C; ^1^H-NMR (400 MHz, CDCl_3_) *δ* 7.24–7.13 (m, 1H), 7.00–6.90 (m, 1H), 6.91–6.82 (m, 1H), 4.24 (dd, *J* = 10.6, 4.6 Hz, 2H), 3.95 (d, *J* = 1.7 Hz, 2H), 3.18 (t, *J* = 6.7 Hz 2H) ppm; ^13^C NMR (150 MHz, CDCl_3_) *δ* 200.8, 173.5, 139.2, 127.0, 125.9, 124.3, 45.5, 35.3, 26.6 ppm; HRMS (ESI): *m*/*z* [M-H]^−^ calcd for C_9_H_9_NOS_3_: 241.9774, found: 241.9773.

**3-(2-(1*H*-Indol-3-yl)ethyl)-2-thioxothiazolidin-4-one (4v):** Amine **1v** (80 mg, 0.5 mmol) was used in general procedure B. The crude product was purified by flash chromatography on silica gel providing **4v** as a yellow solid in 91% yield (126 mg, 0.46 mmol). TLC *R*_f_ = 0.26 (petroleum ether/ethyl acetate, 4:1); M.p. 178.0–179.4 °C; ^1^H-NMR (400 MHz, CDCl_3_) *δ* 8.04 (s, 1H), 7.83-7.71 (m, 1H), 7.40–7.30 (m, 1H), 7.24–7.13 (m, 2H), 7.12–7.06 (m, 1H), 4.34–4.21 (m, 2H), 3.87 (s, 2H), 3.11 (dd, *J* = 9.1, 6.7 Hz, 2H) ppm; ^13^C NMR (150 MHz, CDCl_3_) *δ* 201.3, 173.7, 136.2, 127.4, 122.4, 122.2, 119.7, 118.8, 111.9, 111.2, 45.4, 35.4, 22.6 ppm; HRMS (ESI): *m*/*z* [M-H]^−^ calcd for C_13_H_12_N_2_OS_2_: 275.0318, found: 275.0316.

**4-(2-(4-Oxo-2-thioxothiazolidin-3-yl)ethyl)benzenesulfonamide (4w):** Amine **1w** (100 mg, 0.5 mmol) was used in general procedure B. The crude product was purified by flash chromatography on silica gel providing **4w** as a yellow solid in 89% yield (141 mg, 0.45 mmol). TLC *R*_f_ = 0.24 (petroleum ether/ethyl acetate, 1:1); M.p. 197.6-199.0 °C; ^1^H-NMR (400 MHz, DMSO-*d*_6_) *δ* 7.81–7.71 (m, 2H), 7.47–7.37 (m, 2H), 7.34 (s, 2H), 4.25 (s, 2H), 4.07 (t, *J* =7.5 Hz, 2H), 2.92 (t, *J* = 7.4 Hz, 2H) ppm; ^13^C NMR (150 MHz, DMSO-*d*_6_) *δ* 203.0, 174.1, 142.5, 141.8, 129.2 (2C), 125.9 (2C), 44.6, 35.8, 31.8 ppm; HRMS (ESI): *m*/*z* [M-H]^−^ calcd for C_11_H_12_N_2_O_3_S_3_: 314.9937, found: 314.9934.

**3-Methyl-2-thioxothiazolidin-4-one (4x):** Amine **1x** (16 mg, 0.5 mmol) was used in general procedure B. The crude product was purified by flash chromatography on silica gel providing **4x** as a yellow solid in 96% yield (71 mg, 0.48 mmol). TLC *R*_f_ = 0.29 (petroleum ether/ethyl acetate, 10:1); M.p. 259.4–261.0 °C; ^1^H-NMR (400 MHz, CDCl_3_) *δ* 4.02 (s, 2H), 3.39 (s, 3H) ppm; ^13^C NMR (150 MHz, CDCl_3_) *δ* 201.3, 173.8, 35.7, 31.3 ppm; HRMS (ESI): *m*/*z* [M-H]^−^ calcd for C_4_H_5_NOS_2_: 145.9740, found: 145.9735. NMR and HRMS data are consistent with those previously reported [34].

**3-Butyl-2-thioxothiazolidin-4-one (4y):** Amine **1y** (37 mg, 0.5 mmol) was used in general procedure B. The crude product was purified by flash chromatography on silica gel providing **4y** as a yellow oil in 94% yield (89 mg, 0.47 mmol). TLC *R*_f_ = 0.21 (petroleum ether/ethyl acetate, 10:1); ^1^H-NMR (400 MHz, CDCl_3_) *δ* 4.03–3.92 (m, 4H), 1.69–1.56 (m, 2H), 1.46–1.25 (m, 2H), 1.01–0.89 (m, 3H) ppm; ^13^C NMR (150 MHz, CDCl_3_) *δ* 201.2, 173.9, 44.6, 35.3, 28.8, 20.0, 13.7 ppm; HRMS (ESI): *m*/*z* [M-H]^−^ calcd for C_7_H_11_NOS_2_: 188.0209, found: 188.0200. NMR and HRMS data are consistent with those previously reported [34].

**3-Hexyl-2-thioxothiazolidin-4-one (4z):** Amine **1z** (51 mg, 0.5 mmol) was used in general procedure B. The crude product was purified by flash chromatography on silica gel providing **4z** as a yellow oil in 94% yield (102 mg, 0.47 mmol). TLC *R*_f_ = 0.28 (petroleum ether/ethyl acetate, 10:1); ^1^H-NMR (400 MHz, CDCl_3_) *δ* 4.07–3.89 (m, 4H), 1.71–1.55 (m, 2H), 1.37–1.28 (m, 6H), 0.95–0.83 (m, 3H) ppm; ^13^C NMR (150 MHz, CDCl_3_) *δ* 201.2, 173.9, 44.8, 35.4, 31.3, 26.7, 26.4, 22.5, 14.0 ppm; HRMS (ESI): *m*/*z* [M-H]^−^ calcd for C_9_H_15_NOS_2_: 216.0522, found: 216.0512. NMR and HRMS data are consistent with those previously reported [47].

**3-Decyl-2-thioxothiazolidin-4-one (4aa):** Amine **1aa** (79 mg, 0.5 mmol) was used in general procedure B. The crude product was purified by flash chromatography on silica gel providing **4aa** as a yellow oil in 92% yield (126 mg, 0.46 mmol). TLC *R*_f_ = 0.29 (petroleum ether/ethyl acetate, 10:1); ^1^H-NMR (400 MHz, CDCl_3_) *δ* 4.08–3.82 (m, 4H), 1.70–1.55 (m, 2H), 1.38–1.02 (m, 14H), 0.98–0.76 (m, 3H) ppm; ^13^C NMR (150 MHz, CDCl_3_) *δ* 201.2, 173.9, 44.8, 35.3, 31.9, 29.5, 29.4, 29.3, 29.1, 26.8, 26.7, 22.7, 14.1 ppm; HRMS (ESI): *m*/*z* [M-H]^−^ calcd for C_13_H_23_NOS_2_: 272.1148, found: 272.1150.

**3-Dodecyl-2-thioxothiazolidin-4-one (4ab):** Amine **1ab** (93 mg, 0.5 mmol) was used in general procedure B. The crude product was purified by flash chromatography on silica gel providing **4ab** as a yellow oil in 88% yield (133 mg, 0.44 mmol). TLC *R*_f_ = 0.25 (petroleum ether/ethyl acetate, 10:1); ^1^H-NMR (400 MHz, CDCl_3_) *δ* 4.10–3.82 (m, 4H), 1.72–1.54 (m, 2H), 1.44–1.08 (m, 18H), 0.95–0.78 (m, 3H) ppm; ^13^C NMR (150 MHz, CDCl_3_) *δ* 201.2, 173.9, 44.8, 35.3, 31.9, 29.6 (2C), 29.5, 29.5, 29.3, 29.1, 26.8, 26.7, 22.7, 14.1 ppm; HRMS (ESI): *m*/*z* [M-H]^−^ calcd for C_15_H_27_NOS_2_: 300.1461, found: 300.1464.

**3-Allyl-2-thioxothiazolidin-4-one (4ac):** Amine **1ac** (29 mg, 0.5 mmol) was used in general procedure B. The crude product was purified by flash chromatography on silica gel providing **4ac** as a red oil in 91% yield (79 mg, 0.46 mmol). TLC *R*_f_ = 0.30 (petroleum ether/ethyl acetate, 10:1); ^1^H-NMR (400 MHz, CDCl_3_) *δ* 5.90–5.73 (m, 1H), 5.27 (t, *J* = 14.6 Hz, 2H), 4.61 (d, *J* = 5.9 Hz, 2H), 4.00 (s, 2H) ppm; ^13^C NMR (150 MHz, CDCl_3_) *δ* 200.7, 173.5, 129.4, 119.6, 46.5, 35.4 ppm; HRMS (ESI): *m*/*z* [M-H]^−^ calcd for C_6_H_7_NOS_2_: 171.9896, found: 171.9895. NMR and HRMS data are consistent with those previously reported [34].

**3-(4-((6-Methoxyquinolin-8-yl)amino)pentyl)-2-thioxothiazolidin-4-one (4ad):** Amine **1ad** (130 mg, 0.5 mmol) was used in general procedure B. The crude product was purified by flash chromatography on silica gel providing **4ad** as a yellow oil in 88% yield (165 mg, 0.44 mmol). TLC *R*_f_ = 0.29 (petroleum ether/ethyl acetate, 8:1); ^1^H-NMR (400 MHz, CDCl_3_) *δ* 8.64-8.44 (m, 1H), 7.93 (d, *J* = 8.2 Hz, 1H), 7.36–7.27 (m, 1H), 6.36 (s, 1H), 6.28 (s, 1H), 6.00 (d, *J* = 7.8 Hz, 1H), 3.89 (s, 3H), 3.67 (dd, *J* = 16.3, 9.9 Hz, 1H), 3.59–3.45 (m, 2H), 1.96–1.69 (m, 5H), 1.42–1.21 (m, 4H) ppm; ^13^C NMR (150 MHz, CDCl_3_) *δ* 159.4, 144.7, 144.3, 135.2, 134.9, 130.0, 129.9, 121.9 (2C), 96.9, 91.9 (2C), 55.2, 47.5, 45.1, 33.6, 26.7, 20.7 ppm; HRMS (ESI): *m*/*z* [M-H]^−^ calcd for C_18_H_21_N_3_O_2_S_2_: 374.1002, found: 374.1007.

**3,3′-(1,4-Phenylenebis(methylene))bis(2-thioxothiazolidin-4-one) (4ae):** Diamine **1ae** (34 mg, 0.25 mmol) was used in general procedure B. The crude product was purified by flash chromatography on silica gel providing **4ae** as a yellow solid in 88% yield (81 mg, 0.22 mmol). TLC *R*_f_ = 0.24 (petroleum ether/ethyl acetate, 4:1); M.p. 266.5–268.3 °C; ^1^H-NMR (400 MHz, DMSO-*d*_6_) *δ* 7.24 (s, 4H), 5.03 (s, 4H), 4.34 (s, 4H) ppm; ^13^C NMR (150 MHz, DMSO-*d*_6_) *δ* 203.3 (2C), 174.5 (2C), 134.4 (2C), 127.7 (4C), 46.5 (2C), 36.0 (2C) ppm; HRMS (ESI): *m*/*z* [M-H]^−^ calcd for C_14_H_12_N_2_O_2_S_4_: 366.9709, found: 366.9705.

**3,3′-(Propane-1,3-diyl)bis(2-thioxothiazolidin-4-one) (4af):** Diamine **1af** (19 mg, 0.25 mmol) was used in general procedure B. The crude product was purified by flash chromatography on silica gel providing **4af** as a yellow solid in 89% yield (68 mg, 0.22 mmol). TLC *R*_f_ = 0.29 (petroleum ether/ethyl acetate, 3:1); M.p. 122.8–124.5 °C; ^1^H-NMR (400 MHz, CDCl_3_) *δ* 4.08-4.01 (m, 4H), 4.00 (s, 4H), 2.13–2.00 (m, 2H) ppm; ^13^C NMR (150 MHz, CDCl_3_) *δ* 201.2 (2C), 173.9 (2C), 41.8 (2C), 35.4 (2C), 24.8 ppm; HRMS (ESI): *m*/*z* [M-H]^−^ calcd for C_9_H_10_N_2_O_2_S_4_: 304.9552, found: 304.9549. NMR and HRMS data are consistent with those previously reported [48].

**3,3′-((Ethane-1,2-diylbis(oxy))bis(ethane-2,1-diyl))bis(2-thioxothiazolidin-4-one) (4ag):** Diamine **1ag** (37 mg, 0.25 mmol) was used in general procedure B. The crude product was purified by flash chromatography on silica gel providing **4ag** as a yellow oil in 76% yield (72 mg, 0.19 mmol). TLC *R*_f_ = 0.29 (petroleum ether/ethyl acetate, 3:1); ^1^H-NMR (400 MHz, CDCl_3_) *δ* 7.10-6.99 (m, 4H), 6.91–6.81 (m, 4H), 5.41–4.71 (m, 4H), 3.81–3.75 (m, 4H) ppm; ^13^C NMR (150 MHz, CDCl_3_) *δ* 157.2 (2C), 155.6 (2C), 144.3 (2C), 122.5 (2C), 114.4 (2C), 55.6 (2C) ppm; HRMS (ESI): *m*/*z* [M-H]^−^ calcd for C_12_H_16_N_2_O_4_S_4_: 378.9920, found: 378.9917.

**Ethyl (*S*)-2-(4-oxo-2-thioxothiazolidin-3-yl)-4-phenylbutanoate (4ah):** Amine **1ah** (104 mg, 0.5 mmol) was used in general procedure B. The crude product was purified by flash chromatography on silica gel providing **4ah** as a yellow oil in 81% yield (131 mg, 0.41 mmol). TLC *R*_f_ = 0.23 (petroleum ether/ethyl acetate, 8:1); [α]_D_^25^ = 23.6 (*c* 1.00, CH_3_OH); ^1^H-NMR (400 MHz, CDCl_3_) *δ* 7.29–7.24 (m, 2H), 7.21–7.12 (m, 3H), 5.59 (d, *J* = 8.6 Hz, 1H), 4.27–4.08 (m, 2H), 3.72 (d, *J* = 18.1 Hz, 1H), 3.55 (d, *J* = 18.2 Hz, 1H), 2.82 (dd, *J* = 13.1, 6.8 Hz, 1H), 2.74–2.50 (m, 3H), 1.28–1.20 (m, 3H) ppm; ^13^C NMR (150 MHz, CDCl_3_) *δ* 201.2, 173.3, 167.9, 140.2, 128.5 (2C), 128.2 (2C), 126.2, 62.0, 57.7, 34.3, 32.5, 28.2, 14.1 ppm; HRMS (ESI): *m*/*z* [M-H]^−^ calcd for C_15_H_17_NO_3_S_2_: 322.0577, found: 322.0568.

**3-Cyclohexyl-2-thioxothiazolidin-4-one (4ai):** Amine **1ai** (50 mg, 0.5 mmol) was used in general procedure B. The crude product was purified by flash chromatography on silica gel providing **4ai** as a yellow solid in 91% yield (98 mg, 0.46 mmol). TLC *R*_f_ = 0.22 (petroleum ether/ethyl acetate, 10:1); M.p. 120.3–122.1 °C; ^1^H-NMR (400 MHz, CDCl_3_) *δ* 4.86 (t, *J* = 10.9 Hz, 1H), 3.82 (s, 2H), 2.45-2.15 (m, 2H), 1.94–1.76 (m, 2H), 1.60–1.51 (m, 2H), 1.44–1.31 (m, 2H), 1.32–1.16 (m, 2H) ppm; ^13^C NMR (150 MHz, CDCl_3_) *δ* 202.4, 174.3, 58.5, 33.9, 27.5 (2C), 26.0 (2C), 25.0 ppm; HRMS (ESI): *m*/*z* [M-H]^−^ calcd for C_9_H_13_NOS_2_: 214.0366, found: 214.0365. NMR and HRMS data are consistent with those previously reported [49].

**3-(Tetrahydro-2*H*-pyran-4-yl)-2-thioxothiazolidin-4-one (4aj):** Amine **1aj** (51 mg, 0.5 mmol) was used in general procedure B. The crude product was purified by flash chromatography on silica gel providing **4aj** as a white solid in 92% yield (100 mg, 0.46 mmol). TLC *R*_f_ = 0.27 (petroleum ether/ethyl acetate, 4:1); M.p. 160.7–162.1 °C; ^1^H-NMR (400 MHz, CDCl_3_) *δ* 5.13 (t, *J* = 12.2 Hz, 1H), 4.08 (d, *J* = 11.3 Hz, 2H), 3.86 (s, 2H), 3.46 (t, *J* = 11.9 Hz, 2H), 2.78–2.61 (m, 2H), 1.60–1.49 (m, 2H) ppm; ^13^C NMR (150 MHz, CDCl_3_) *δ* 202.1, 174.0, 67.6 (2C), 55.3, 33.9, 27.7 (2C) ppm; HRMS (ESI): *m*/*z* [M-H]^−^ calcd for C_8_H_11_NO_2_S_2_: 216.0158, found: 216.0160. NMR and HRMS data are consistent with those previously reported [49].

**3-(3-Oxocyclobutyl)-2-thioxothiazolidin-4-one (4ak):** Amine **1ak** (43 mg, 0.5 mmol) was used in general procedure B. The crude product was purified by flash chromatography on silica gel providing **4ak** as a yellow solid in 88% yield (89 mg, 0.44 mmol). TLC *R*_f_ = 0.28 (petroleum ether/ethyl acetate, 4:1); M.p. 166.0–167.6 °C; ^1^H-NMR (400 MHz, CDCl_3_) *δ* 5.85–5.65 (m, 1H), 4.00–3.91 (m, 4H), 3.45–3.34 (m, 2H) ppm; ^13^C NMR (150 MHz, CDCl_3_) *δ* 201.8, 201.4, 174.2, 51.0 (2C), 41.9, 34.3 ppm; HRMS (ESI): *m*/*z* [M-H]^−^ calcd for C_7_H_7_NO_2_S_2_: 199.9845, found: 199.9847.

**3-(1-methyl-2-oxo-5-phenyl-2,3-dihydro-1*H*-benzo[e][1,4]diazepin-3-yl)-2-thioxothiazolidin-4-one (4al):** Amine **1al** (133 mg, 0.5 mmol) was used in general procedure B. The crude product was purified by flash chromatography on silica gel providing **4al** as a white solid in 92% yield (176 mg, 0.46 mmol). TLC *R*_f_ = 0.30 (petroleum ether/ethyl acetate, 4:1); M.p. 178.5-180.1 °C; ^1^H-NMR (400 MHz, CDCl_3_) *δ* 7.68–7.58 (m, 3H), 7.50–7.31 (m, 5H), 7.28 (s, 1H), 6.75 (s, 1H), 4.10 (s, 2H), 3.46 (s, 3H) ppm; ^13^C NMR (150 MHz, CDCl_3_) *δ* 202.1, 172.0, 168.0, 166.8, 143.0, 137.9, 132.2, 130.8, 130.7, 130.0 (2C), 128.3, 128.2 (2C), 124.4, 121.4, 74.8, 35.8, 34.3 ppm; HRMS (ESI): *m*/*z* [M+H]^+^ calcd for C_19_H_15_N_3_O_2_S_2_: 382.0678, found: 382.0681.

### 3.4. General Procedure for the Synthesis of 4-Methoxyphenyl Carbamate (5a)

A mixture of *p*-tolylmethanamine (61 mg, 0.5 mmol), carbon disulfide (38 mg, 0.5 mmol), 4-methoxyphenyl (2-chloroacetyl)carbamate (122 mg, 0.5 mmol), and triethylamine (61 mg, 0.6 mmol) in MeCN (3 mL) was stirred at room temperature for 10 min. After disappearance of the reactant (monitored by TLC), added 50 mL water to the mixture, and then extracted with ethyl acetate 3 times (3 × 50 mL). The extract was dried over anhydrous Na_2_SO_4_ and evaporated. The residue was purified by column chromatography on silica gel to afford the product **4a** (TLC *R*_f_ = 0.24, petroleum ether/ethyl acetate = 10:1) as a red solid in 94% yield (112 mg, 0.47 mmol) and **5a** (TLC *R*_f_ = 0.30, petroleum ether/ethyl acetate = 3:1) as a white solid in 80% yield (67 mg, 0.40 mmol). M.p. 127.3–129.0 ^o^C; ^1^H-NMR (400 MHz, CDCl_3_) *δ* 7.15–7.00 (m, 2H), 6.95–6.83 (m, 2H), 5.05 (s, 2H), 3.80 (s, 3H); ^13^C NMR (150 MHz, CDCl_3_) *δ* 157.2, 155.5, 144.3, 122.5 (2C), 114.4 (2C), 55.6; HRMS (ESI): *m*/*z* [M+H]^+^ calcd for C_8_H_9_NO_3_: 168.0655, found: 168.0654.

CCDC 1970414 (**4af**), CCDC 1970415 (**4al**), and CCDC 1970413 (**5a**) contain the Supplementary Crystallographic Data for this paper. These data can be obtained free of charge via http://www.ccdc.cam.ac.uk/conts/retrieving.html (or from the CCDC, 12 Union Road, Cambridge CB2 1EZ, UK; Fax: +44 1223 336033; E-mail: deposit@ccdc.cam.ac.uk).

## 4. Conclusions

In summary, we established an efficient approach for the synthesis of *N*-substituted rhodanines via a base-assisted one-pot coupling and continuous cyclization of a primary amine, carbon disulphide, and methyl (2-chloroacetyl)carbamate. A series of substituted alkyl- and benzylamines were successively obtained, providing structurally diverse *N*-substituted rhodanines in outstanding yield. Further application of this promising method in pharmaceutical synthesis is in progress in our laboratory.

## Figures and Tables

**Table 1:**
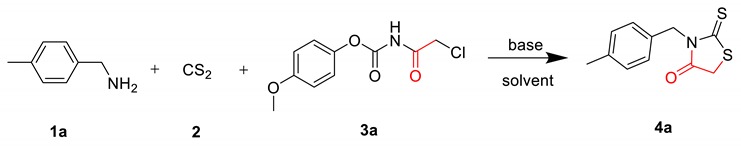
Optimization of reaction conditions ^a.^

Entry	Solvent	Base	Yield ^b^ (%)
1	MeCN	DBU	74
2	MeCN	DIPEA	90
3	MeCN	Et_3_N	94
4	MeCN	K_2_CO_3_	82
5	MeCN	Na_2_CO_3_	84
6	MeCN	K_3_PO_4_	88
7	MeCN	DMAP	80
8	DMSO	Et_3_N	81
9	DMA	Et_3_N	84
10	EA	Et_3_N	86
11	acetone	Et_3_N	88
12	toluene	Et_3_N	82
13	DCM	Et_3_N	84
14	DCE	Et_3_N	88
15	MeOH	Et_3_N	76
16	EtOH	Et_3_N	74
17	DMF	Et_3_N	76
18	MeCN	−	0

^a^ Reaction conditions: **1a** (0.5 mmol), **2** (0.5 mmol), **3a** (0.5 mmol), base (0.6 mmol), solvent (3 mL), r. t., 10 min. ^b^ Isolated yield based on **1a**. DMSO = dimethyl sulfoxide; DMA = *N*,*N*-dimethylacetamide; EA = ethyl acetate; DCM = dichloromethane; DCE = dichloroethane; DMF = *N*,*N*-dimethylformamide; DBU = 1,8-diazabicyclo[5.4.0]undec-7-ene; DIPEA = *N*,*N*-diisopropylethylamine; DMAP = 4-dimethylaminopyridine.

## Supplementary Materials

The following are available online. Supporting information including the spectrums of ^1^H, ^13^C-NMR, ^19^F-NMR, and X-ray Crystallography.
