# Supplementary material for: A Concise Approach to N-Substituted Rhodanines through a Base-Assisted One-Pot Coupling and Cyclization Process
Source: Molecules. 2020 Mar 4;25(5):1138. doi: 10.3390/molecules25051138 (PMC7179173; doi:10.3390/molecules25051138)
Supplement: Supplementary file 1 [file molecules-25-01138-s001.pdf]

Supporting Information

A Concise Approach to N-substituted Rhodanines through a Base-assisted One-pot Coupling and Cyclization Process

Yongxi Liang, [1] Mei-Lin Tang, [1,3] Zhipeng Huo, [1] Chenchen Zhang [1] and Xun Sun [1,2,*]

[1] Department of Natural Medicine, School of Pharmacy, Fudan University, 826 Zhangheng Road, Shanghai, 201203, China

[2] The Institutes of Integrative Medicine of Fudan University, 12 Wulumuqi Zhong Road, Shanghai 200040, China

[3] State Key Laboratory of Molecular Engineering and Institutes of Biomedical Sciences, Fudan University, 220 Handan Road, Shanghai 200433, China

* Correspondence: sunxunf@shmu.edu.cn (X.S.); Tel./Fax: +86-21-51980101

Table of Contents

1 Copies of ^{1}H NMR, ^{13}C NMR and ^{19}F NMR Spectrum for All Compounds....................S2

2 X-ray Crystallography of Compounds 4af, 4al and 5a....................S52

1 Copies of ^{1}H NMR, ^{13}C NMR and ^{19}F NMR Spectrum for All Compounds

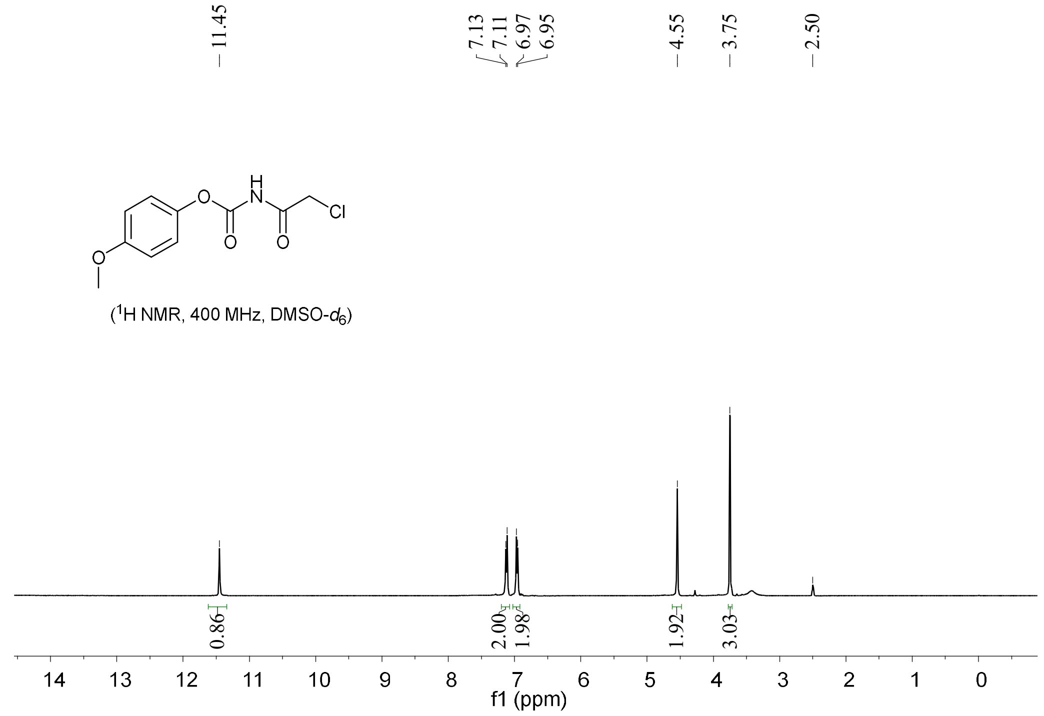

^{1}H NMR of compound **3a**

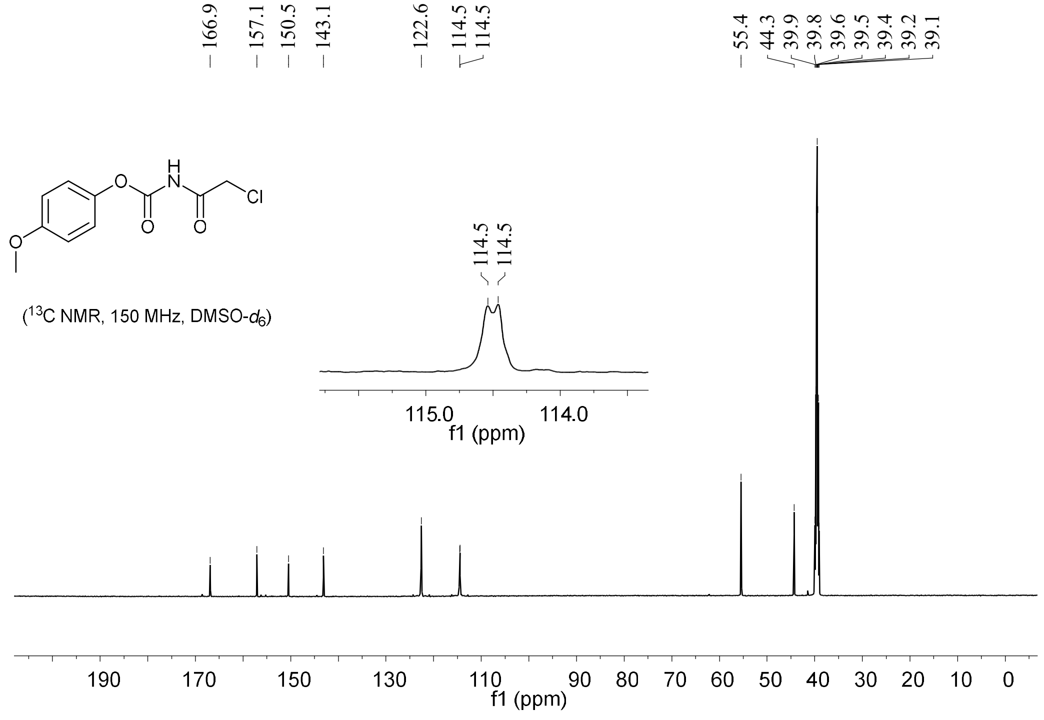

^{13}C NMR of compound **3a**

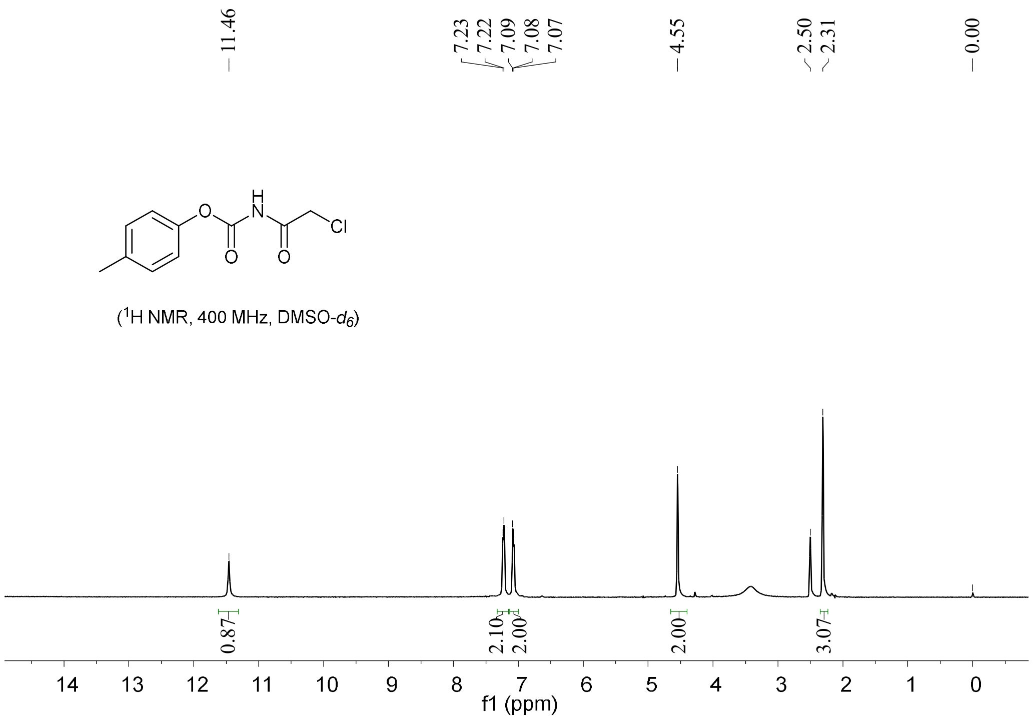

^{1}H NMR of compound **3b**

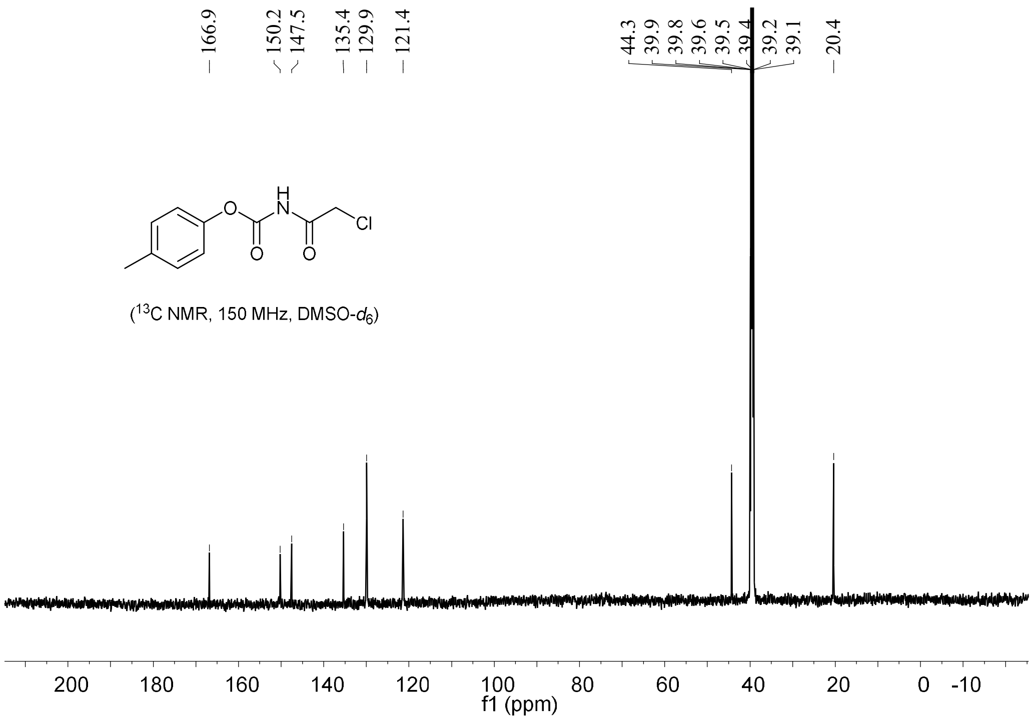

^{13}C NMR of compound **3b**

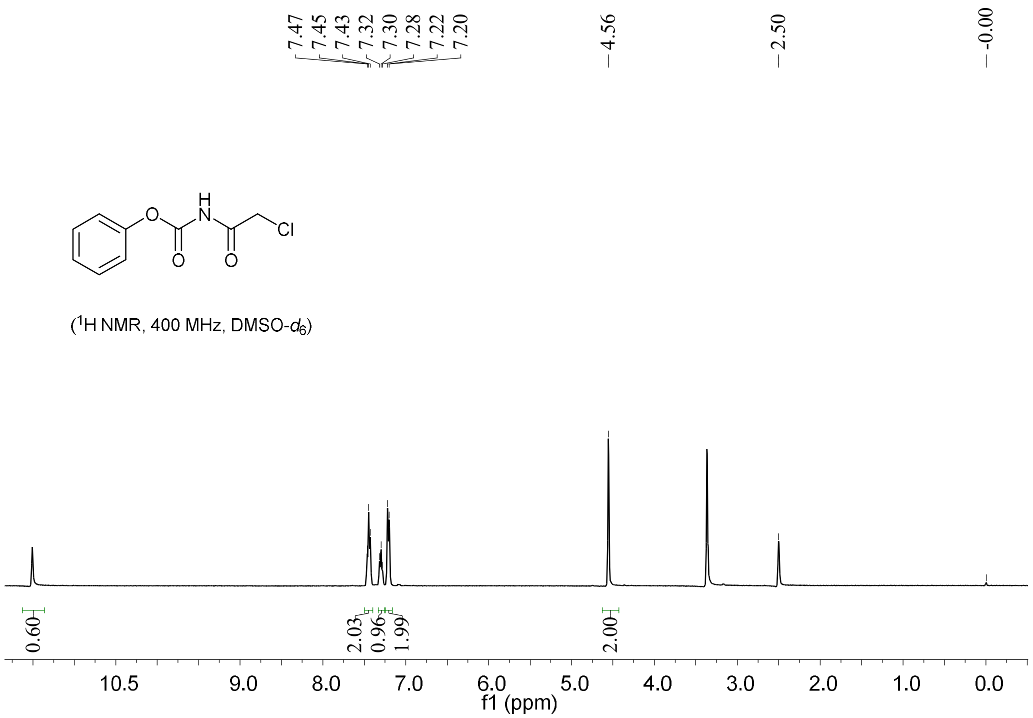

^{1}H NMR of compound **3c**

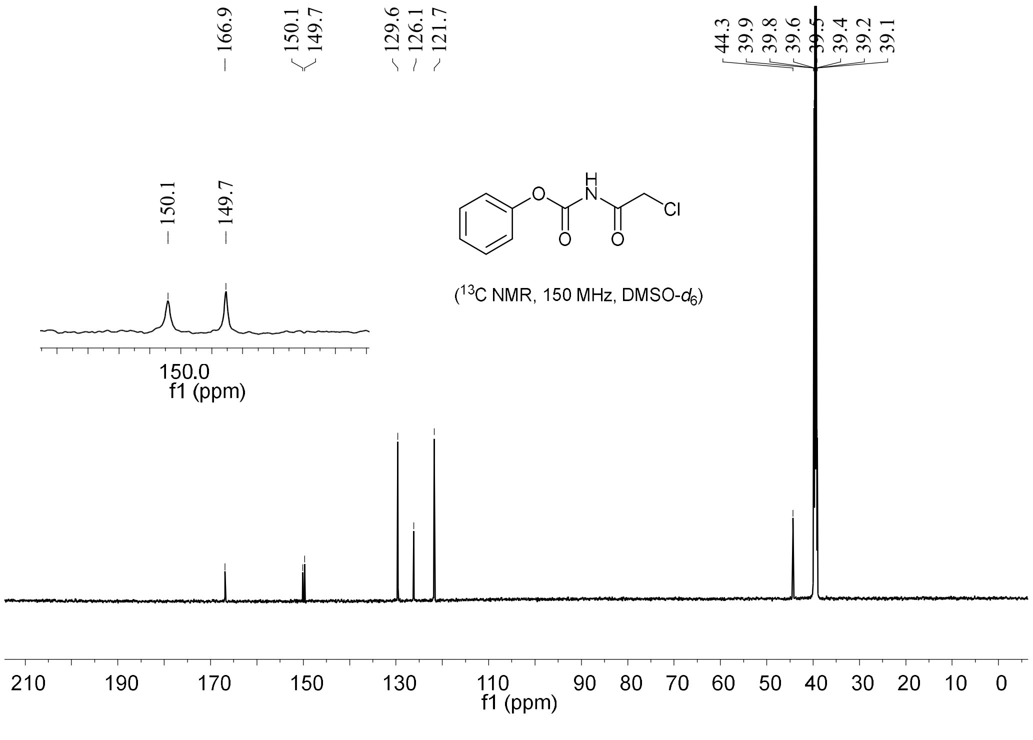

^{13}C NMR of compound **3c**

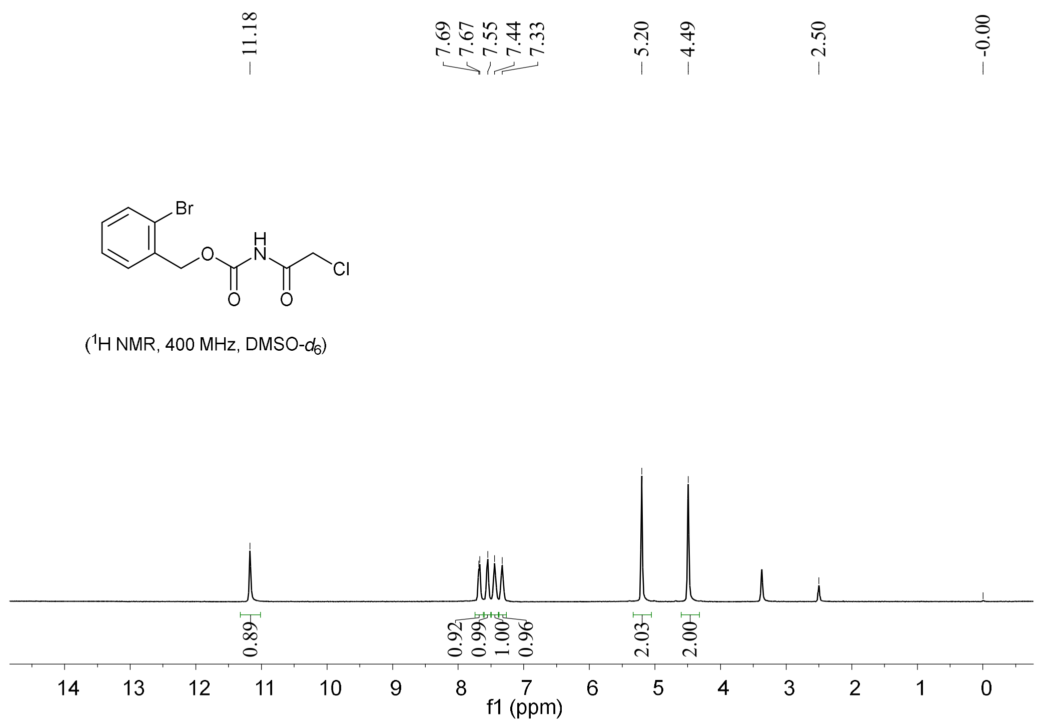

^{1}H NMR of compound **3d**

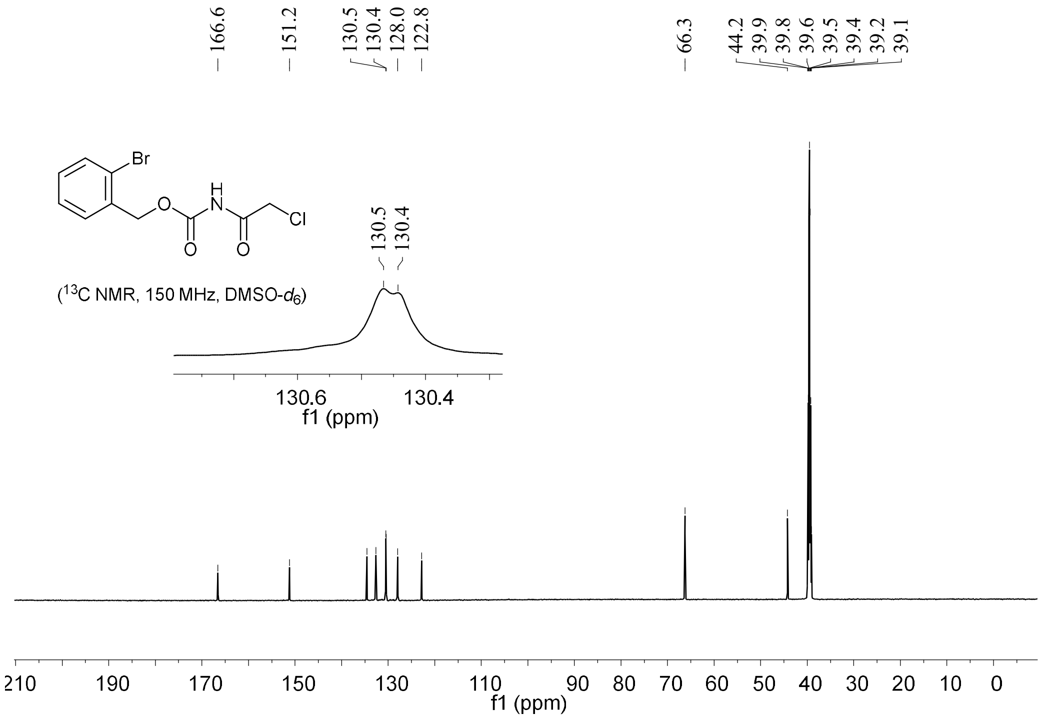

^{13}C NMR of compound **3d**

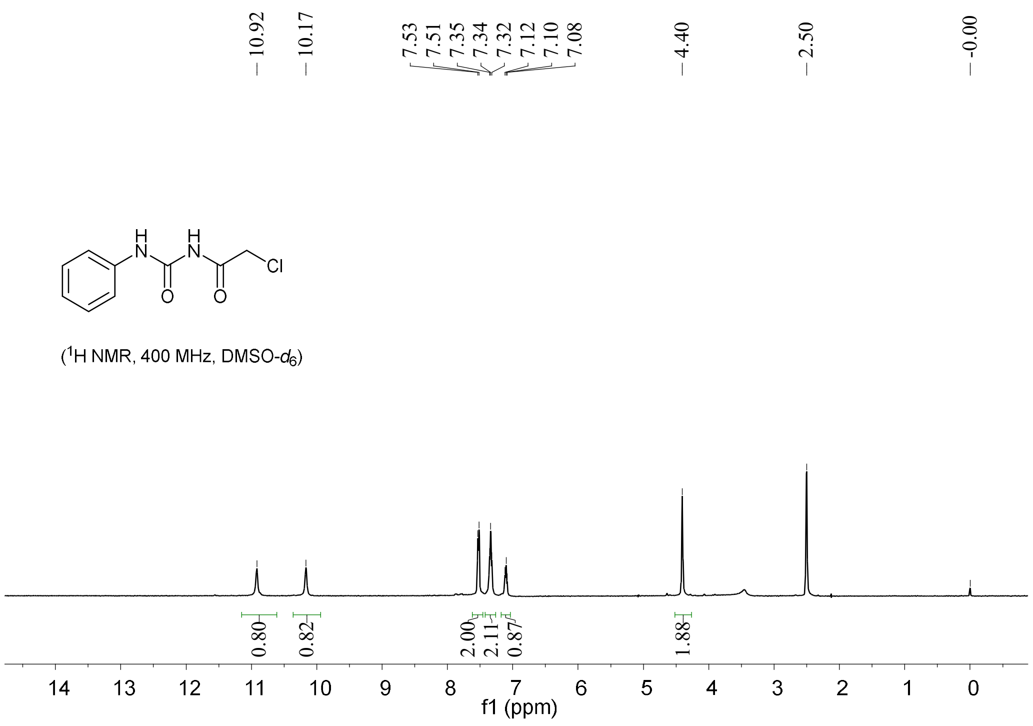

^{1}H NMR of compound **3e**

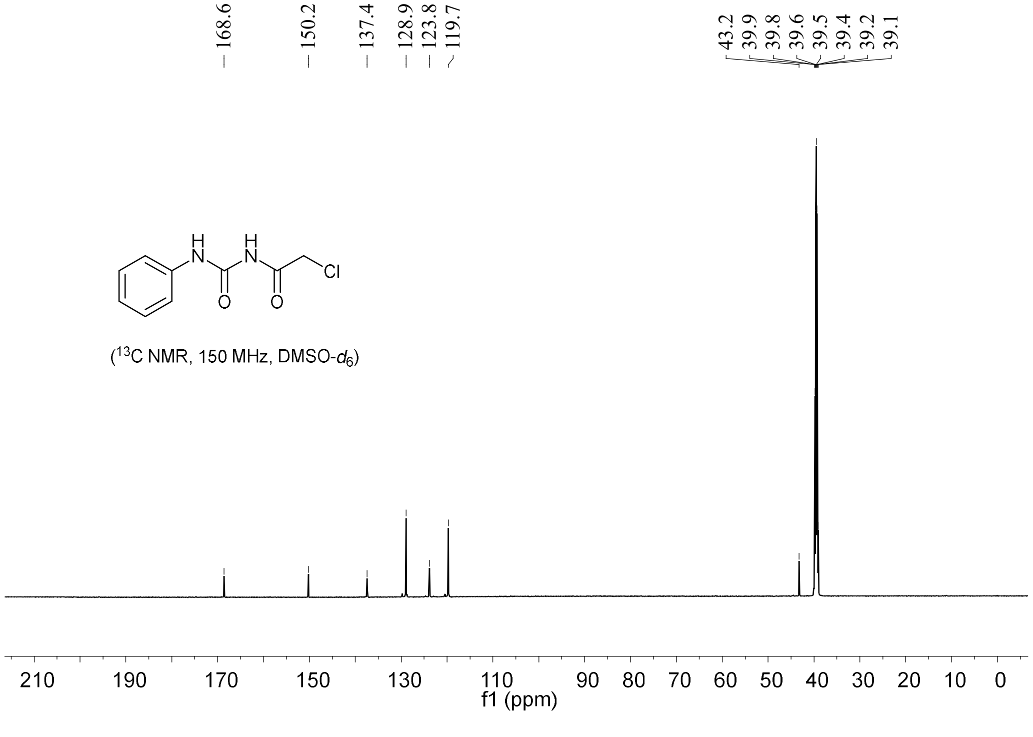

^{13}C NMR of compound **3e**

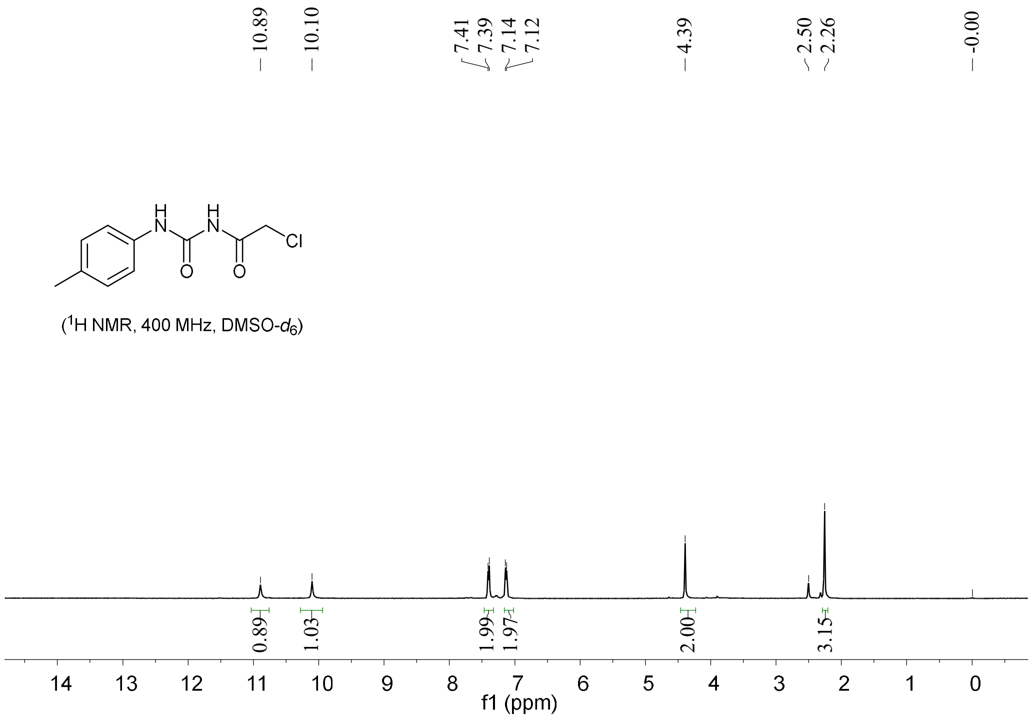

^{1}H NMR of compound **3f**

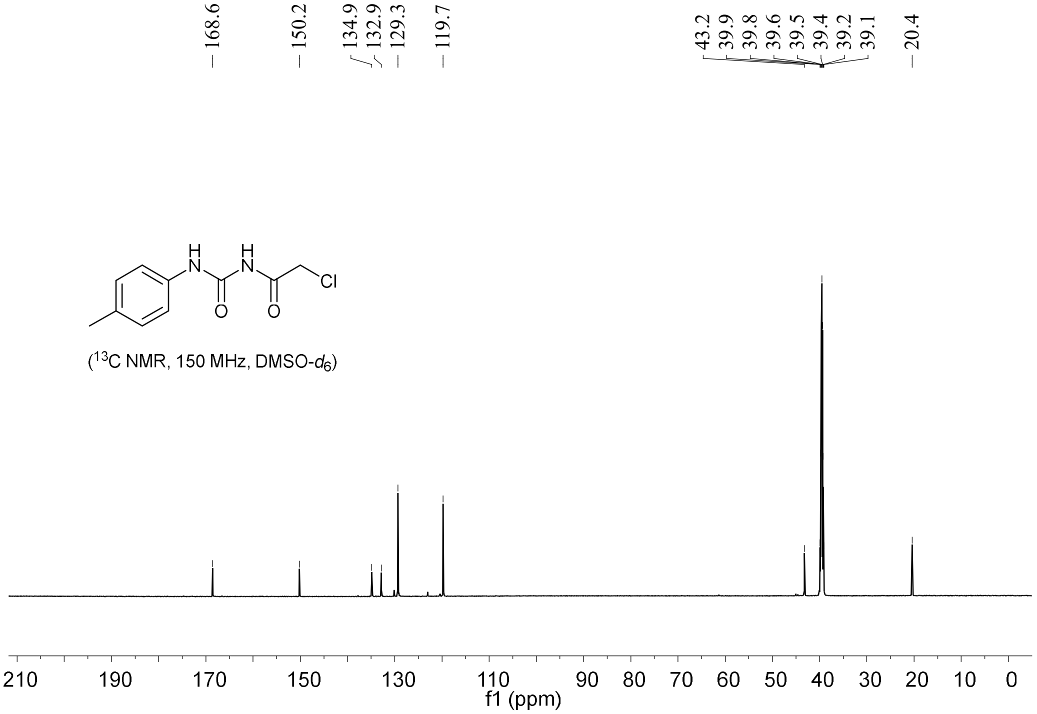

^{13}C NMR of compound **3f**

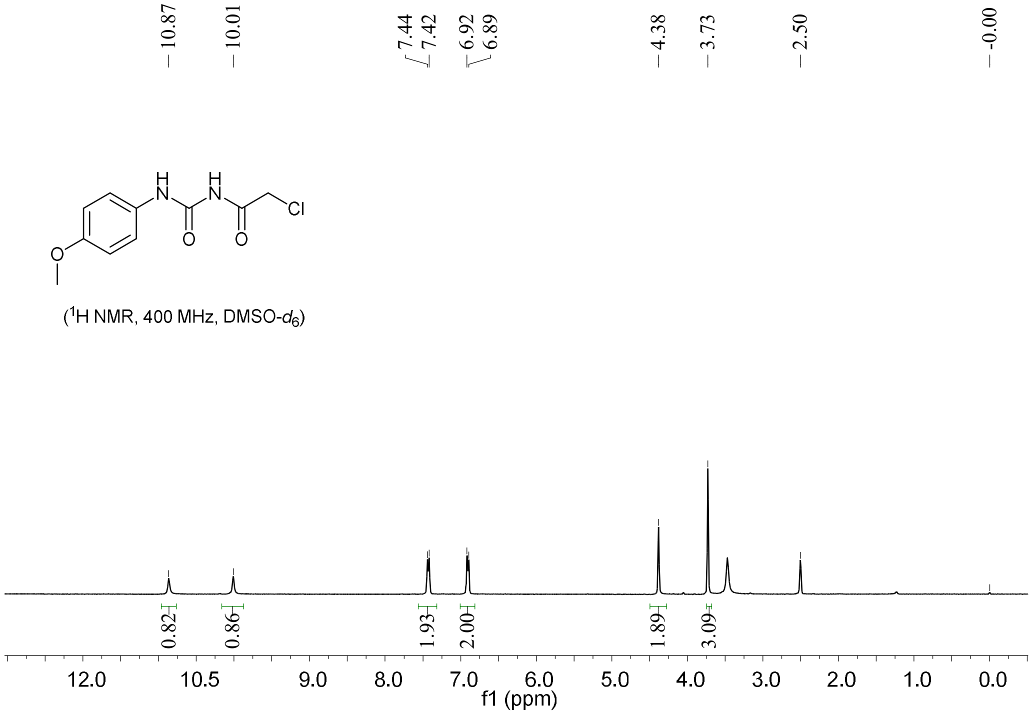

^{1}H NMR of compound **3g**

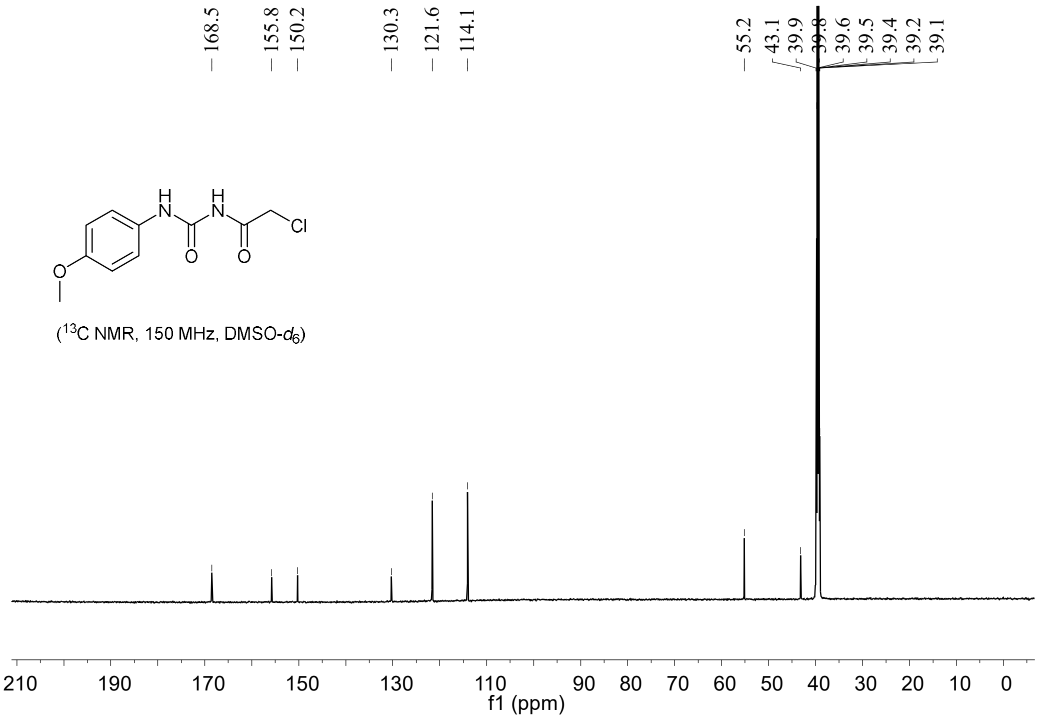

^{13}C NMR of compound **3g**

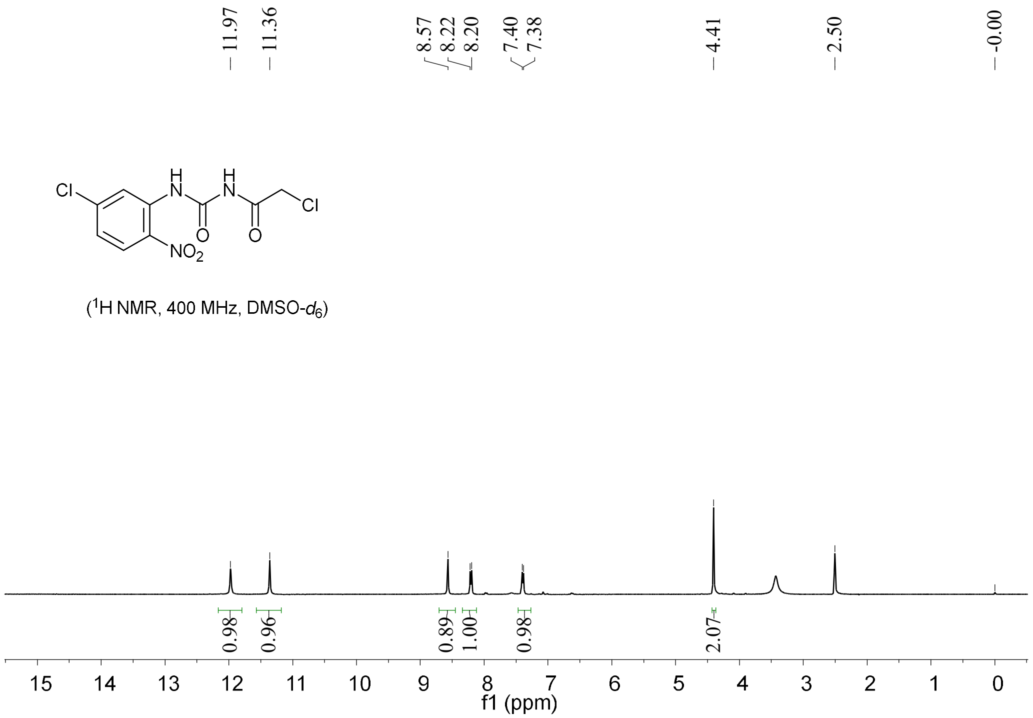

^{1}H NMR of compound **3h**

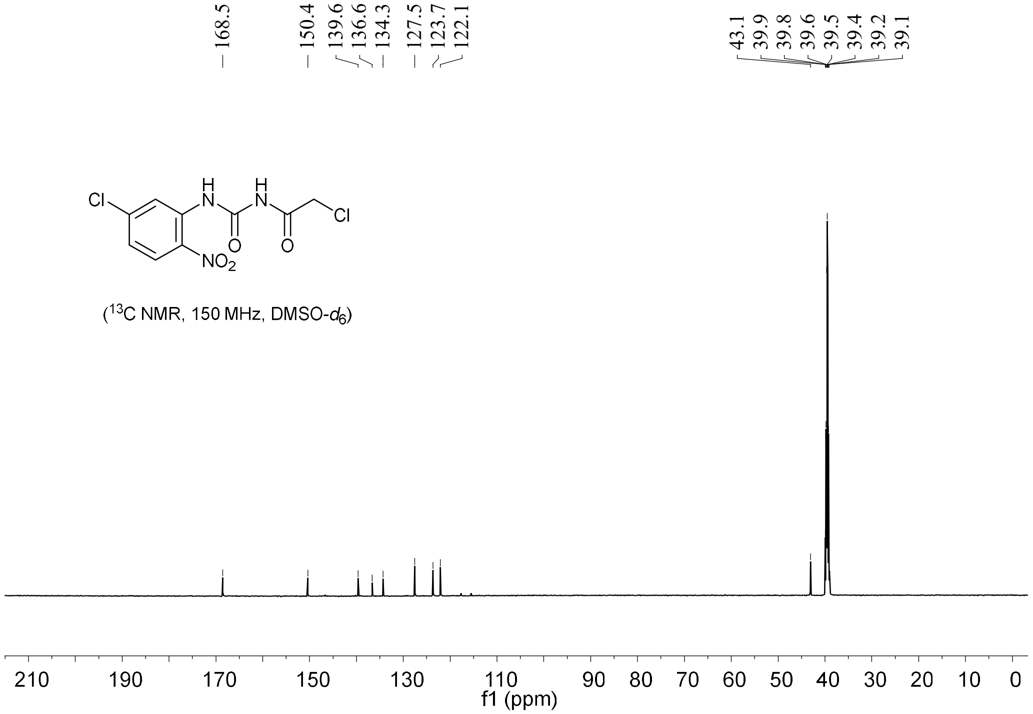

^{13}C NMR of compound **3h**

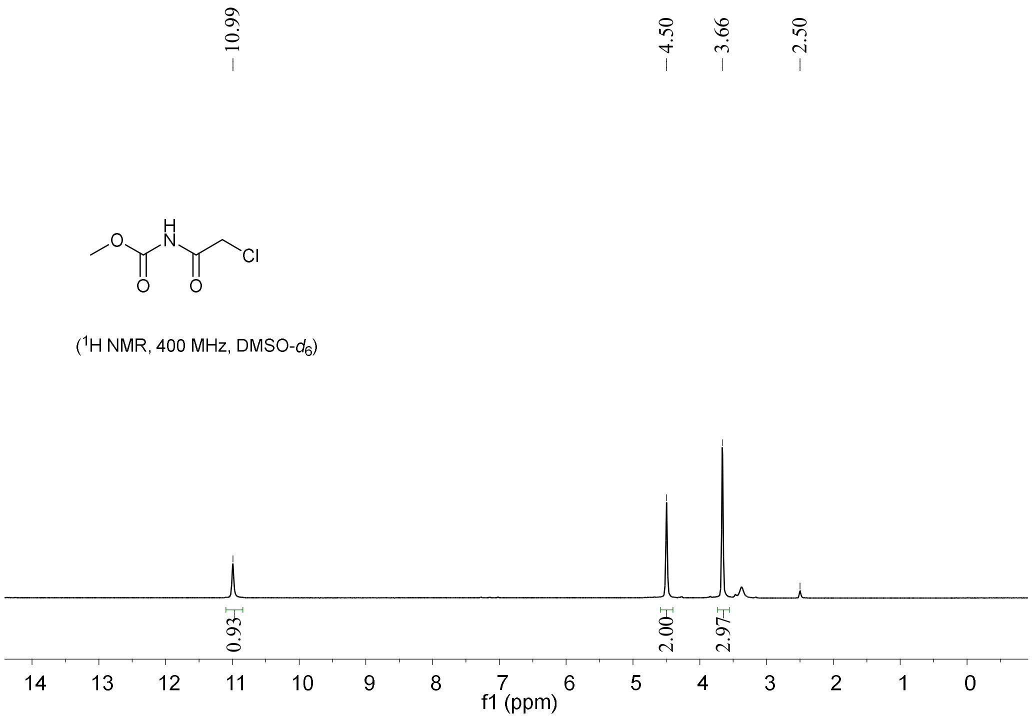

^{1}H NMR of compound **3i**

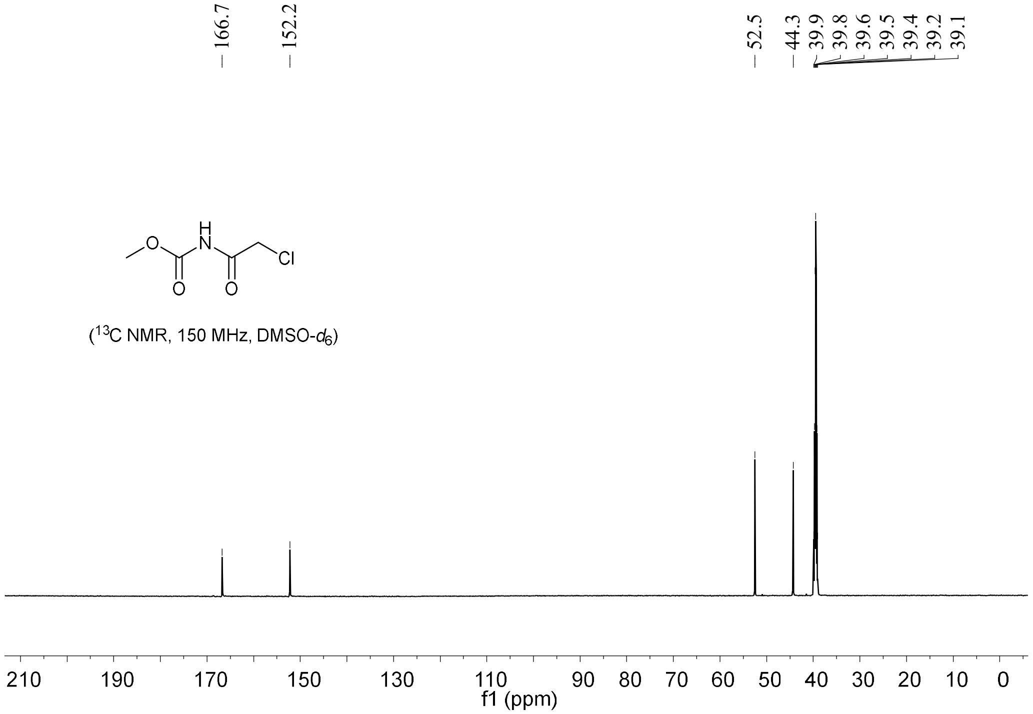

^{13}C NMR of compound **3i**

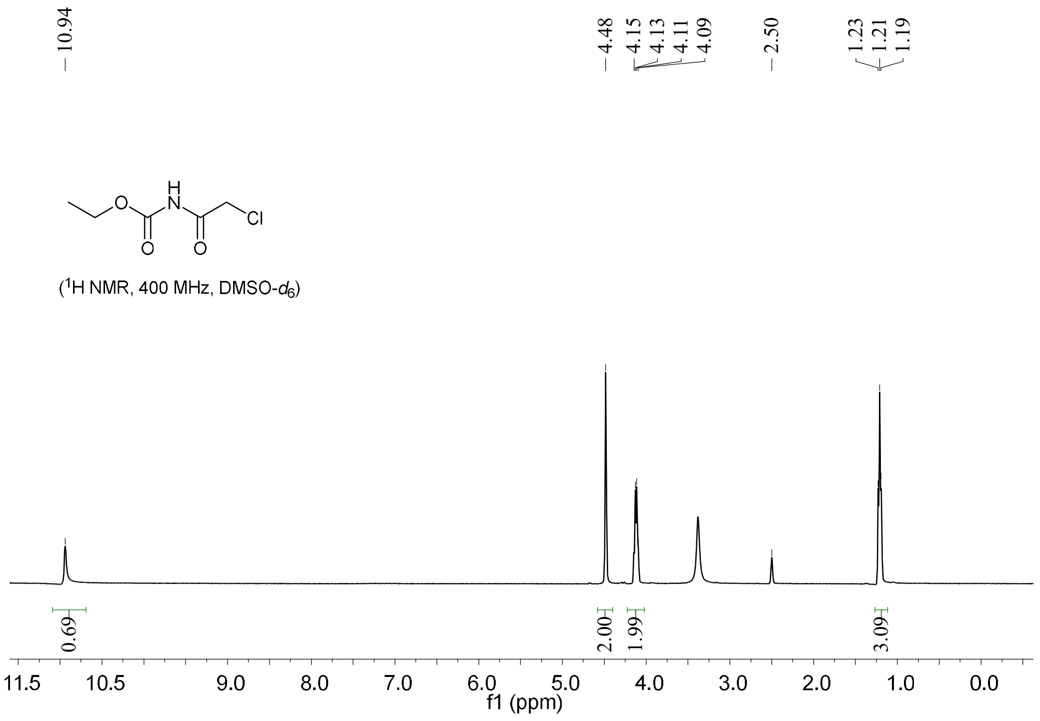

^{1}H NMR of compound **3j**

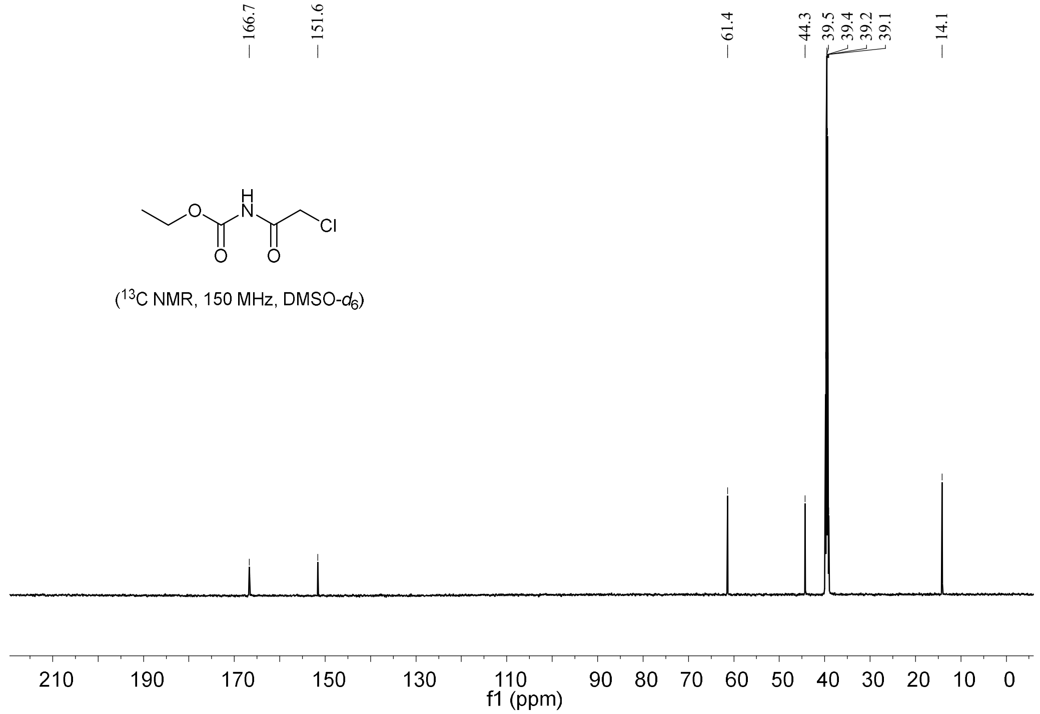

^{13}C NMR of compound **3j**

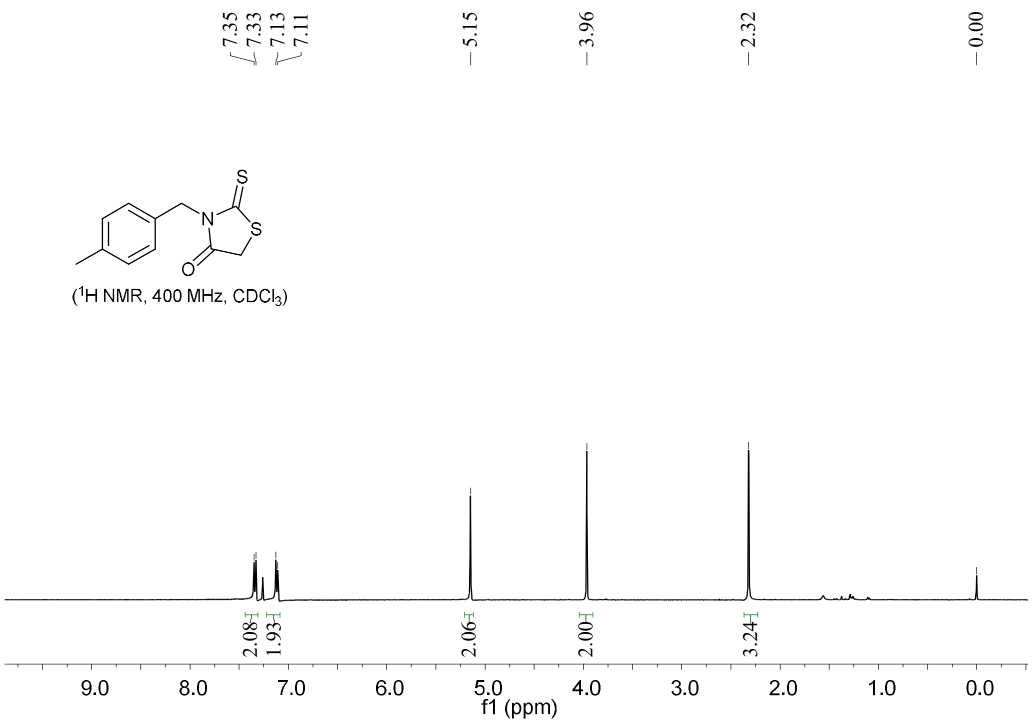

^{1}H NMR of compound **4a**

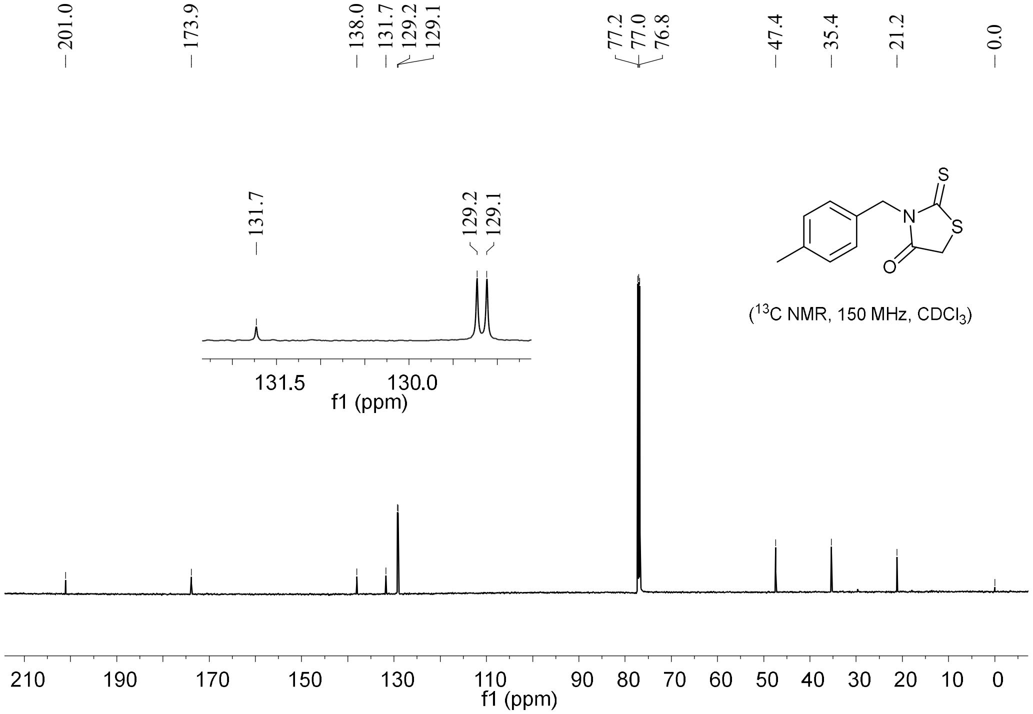

^{13}C NMR of compound **4a**

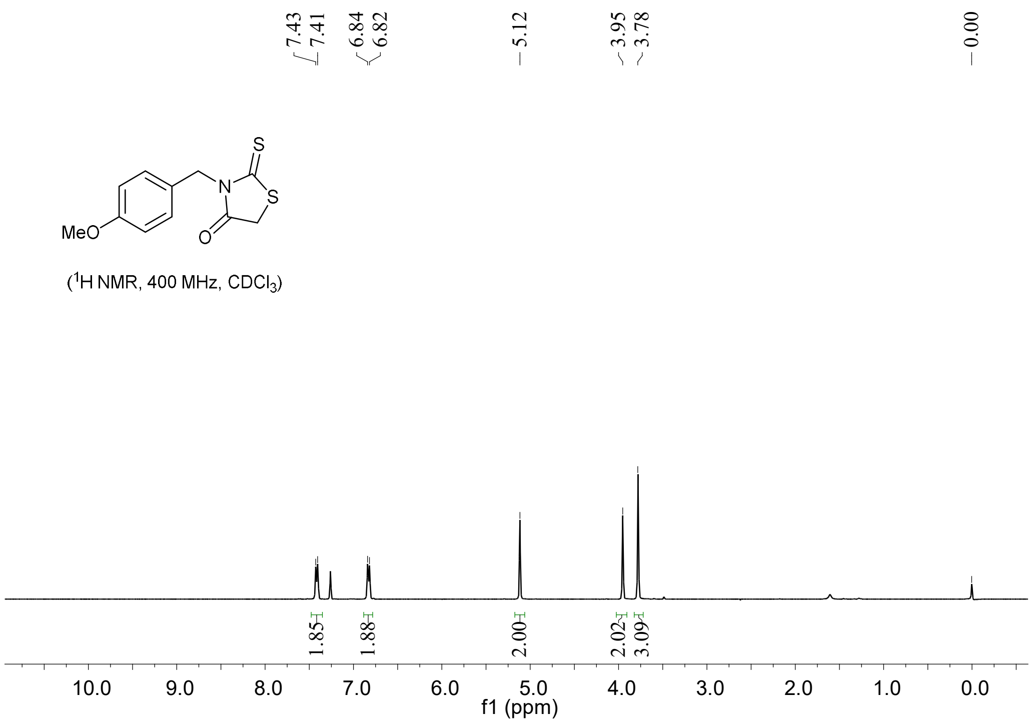

^{1}H NMR of compound **4b**

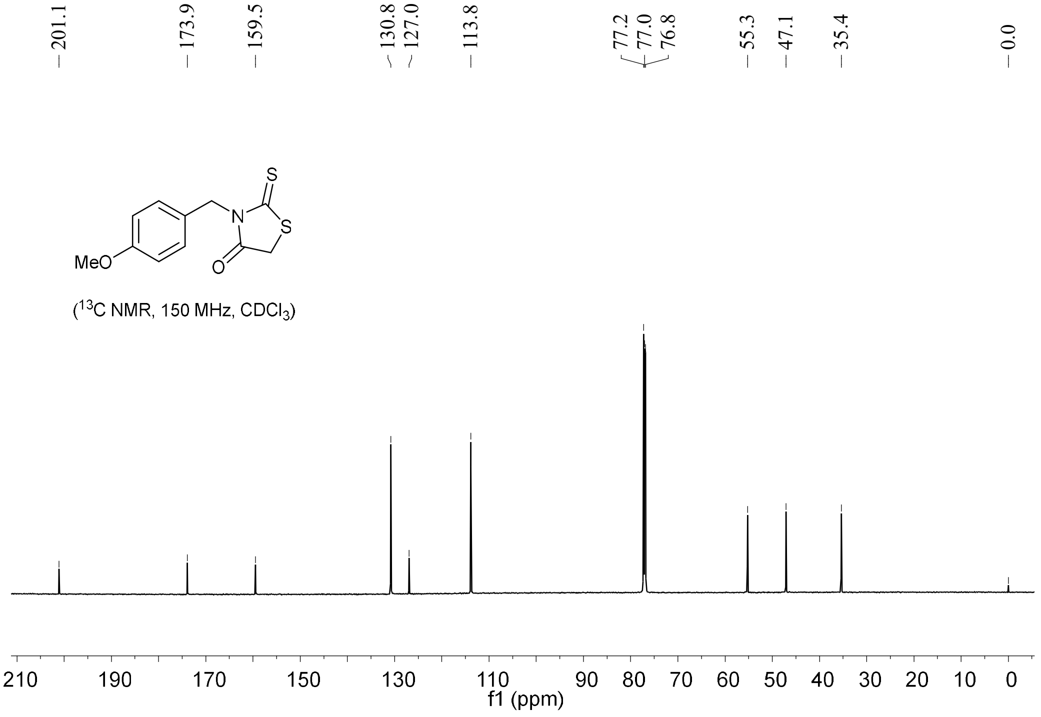

^{13}C NMR of compound **4b**

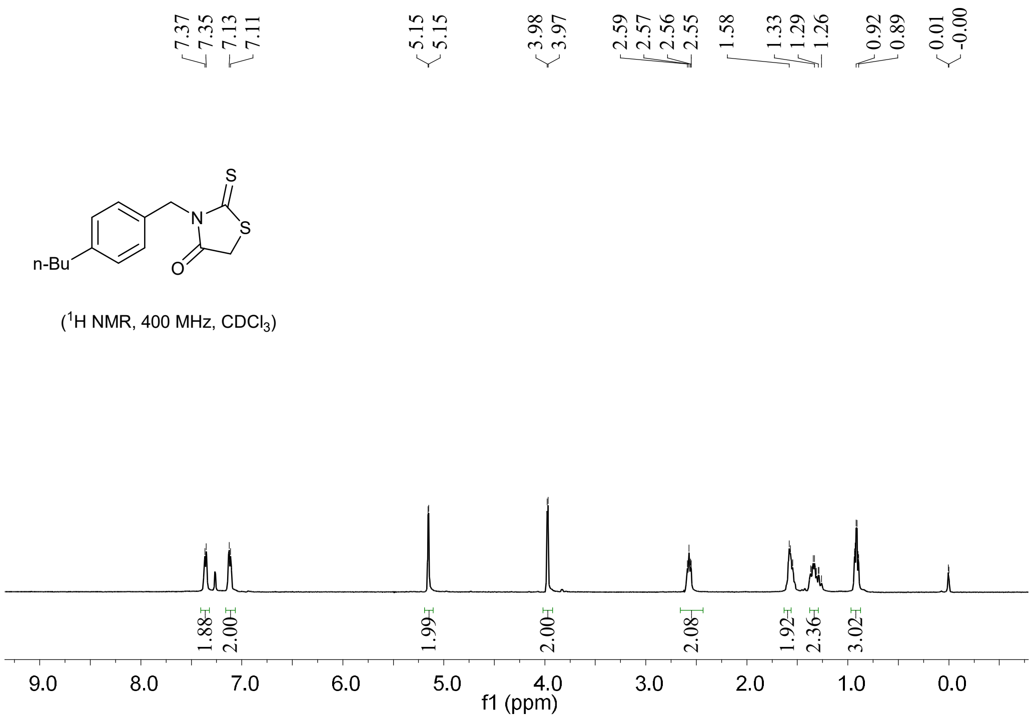

^{1}H NMR of compound **4c**

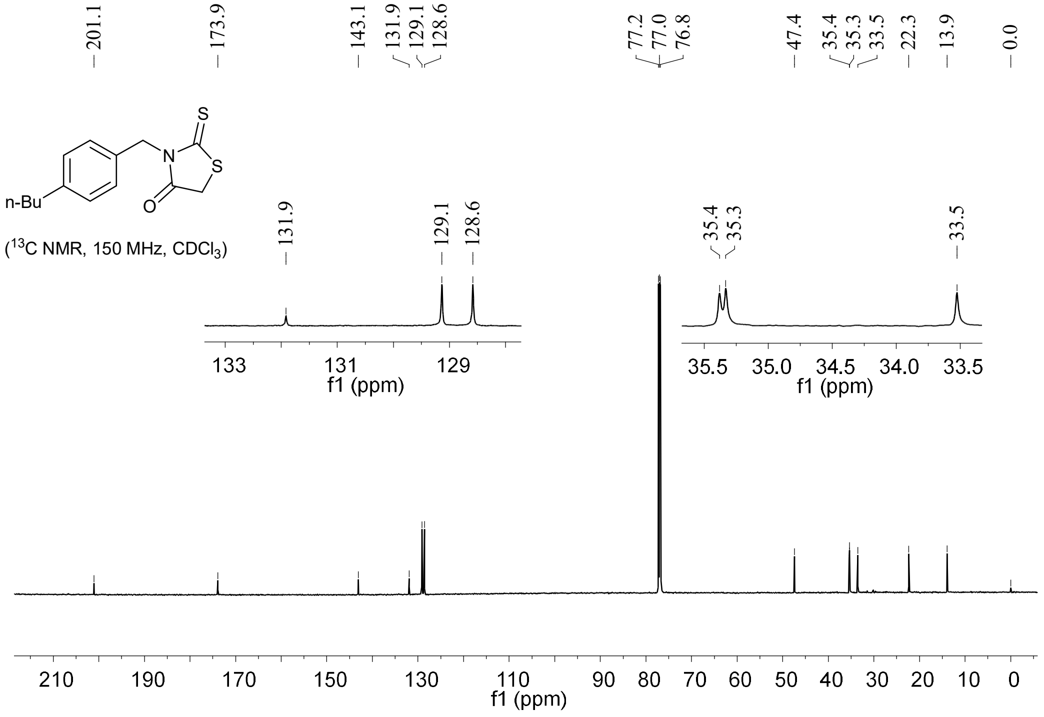

^{13}C NMR of compound **4c**

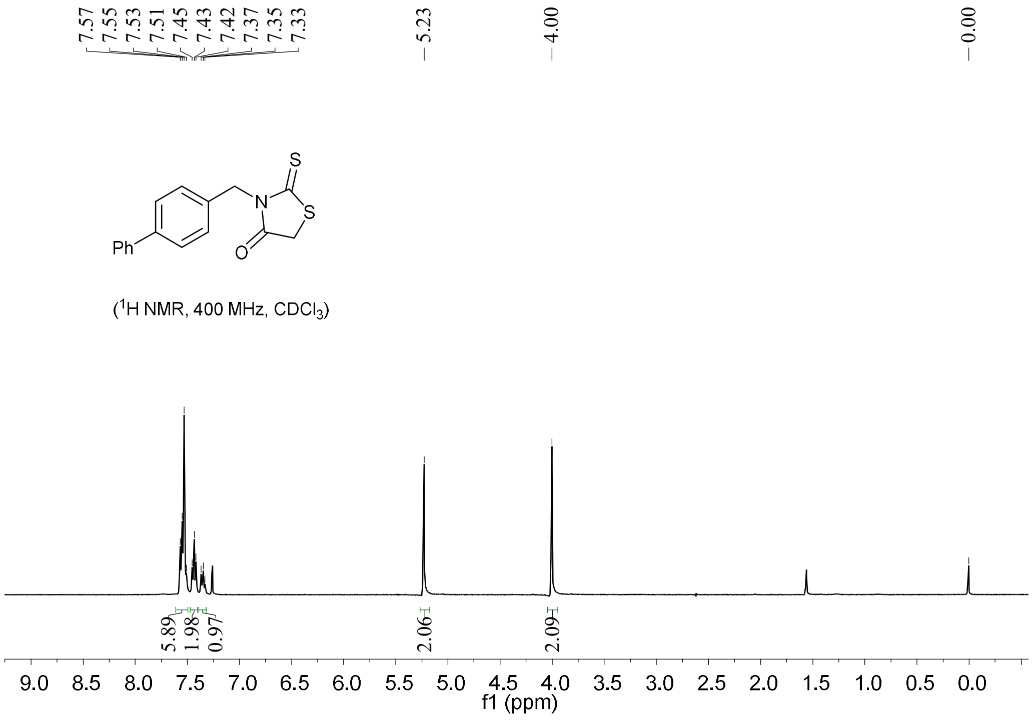

^{1}H NMR of compound **4d**

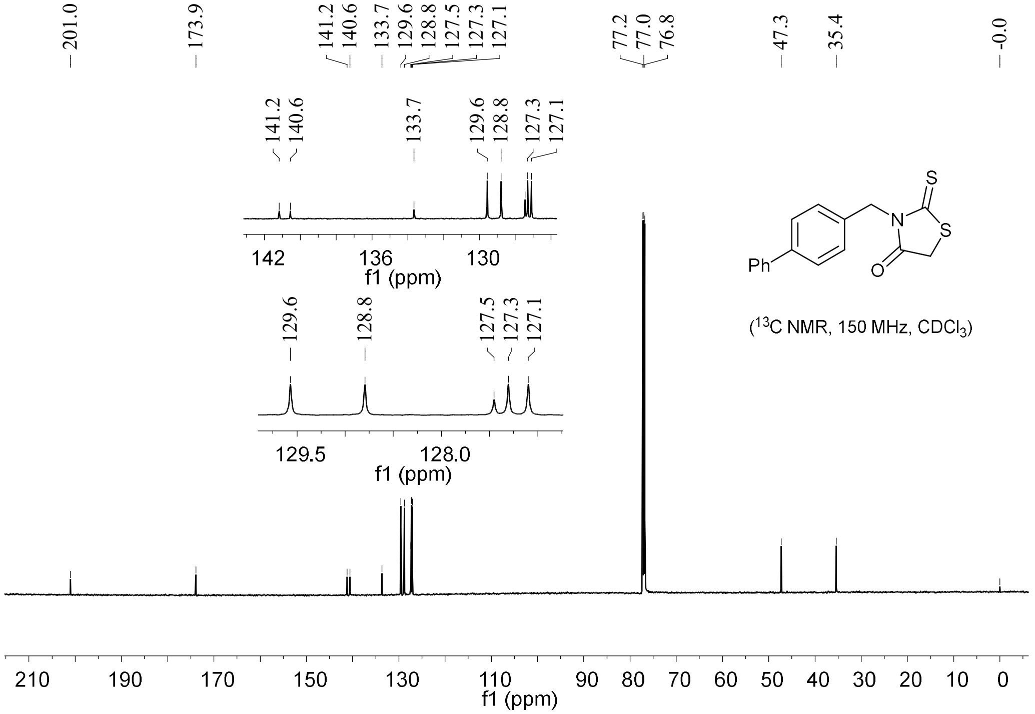

^{13}C NMR of compound **4d**

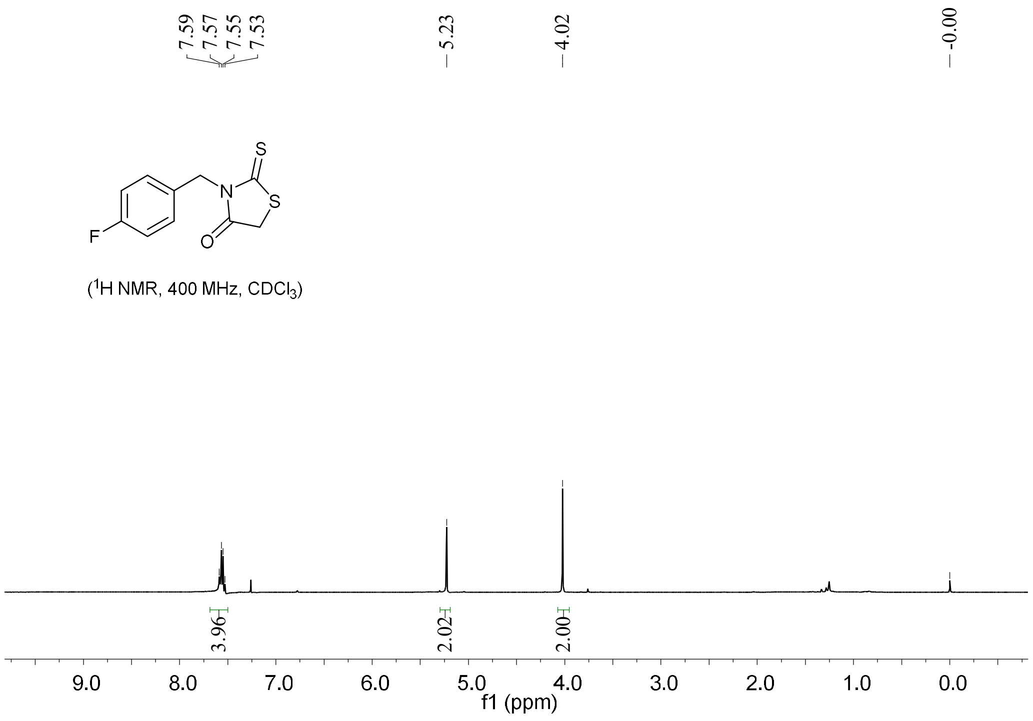

^{1}H NMR of compound **4e**

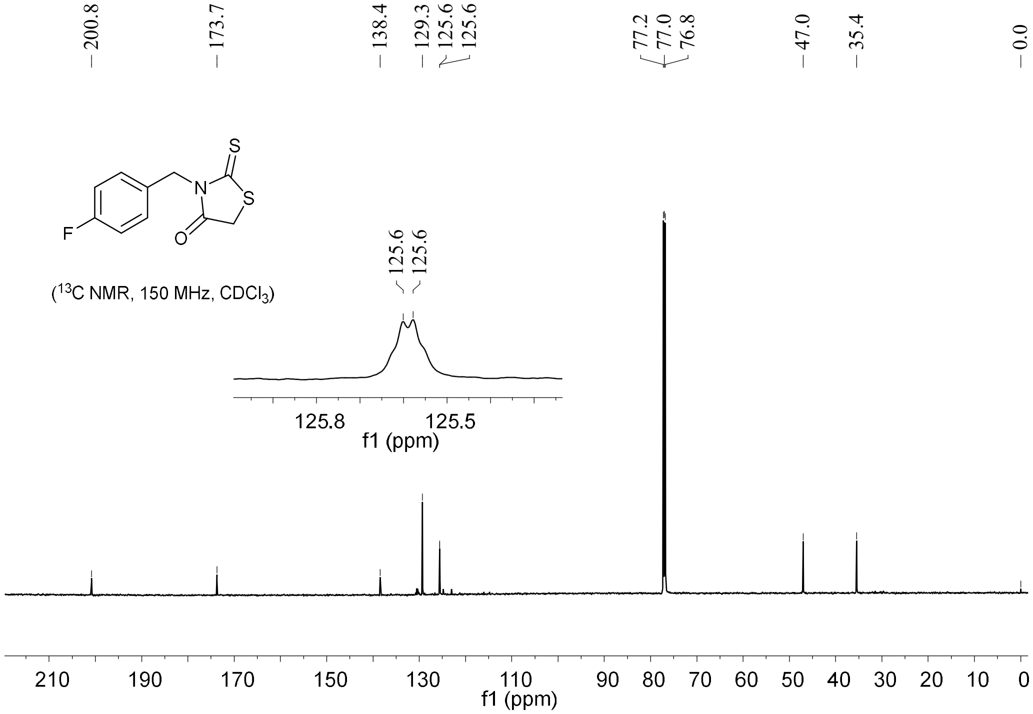

^{13}C NMR of compound **4e**

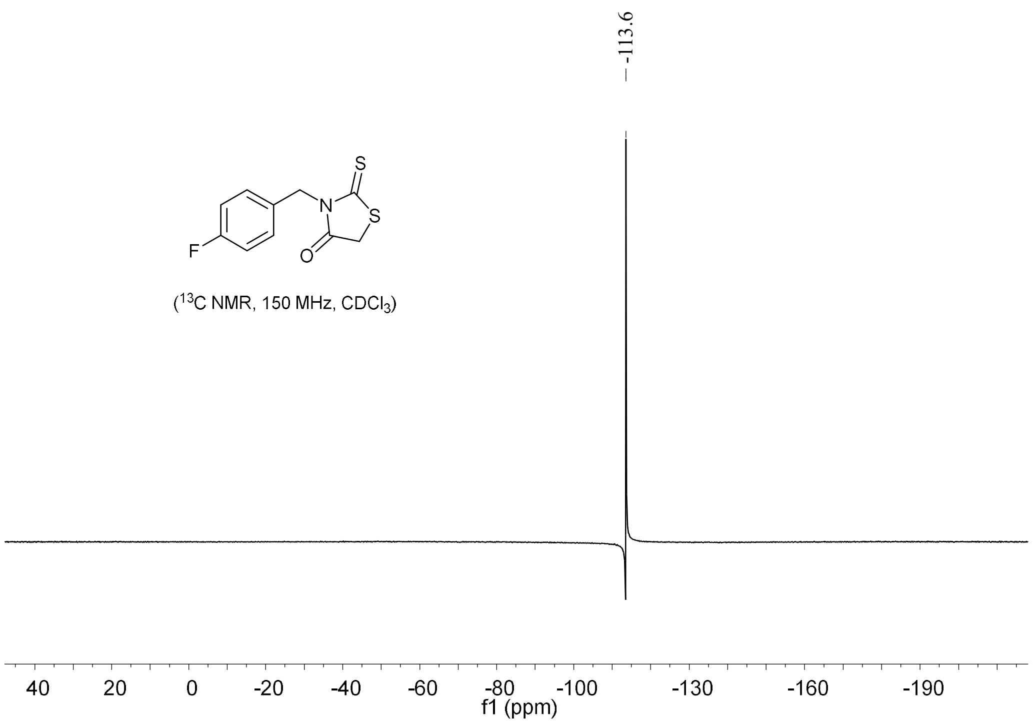

^{19}F NMR of compound **4e**

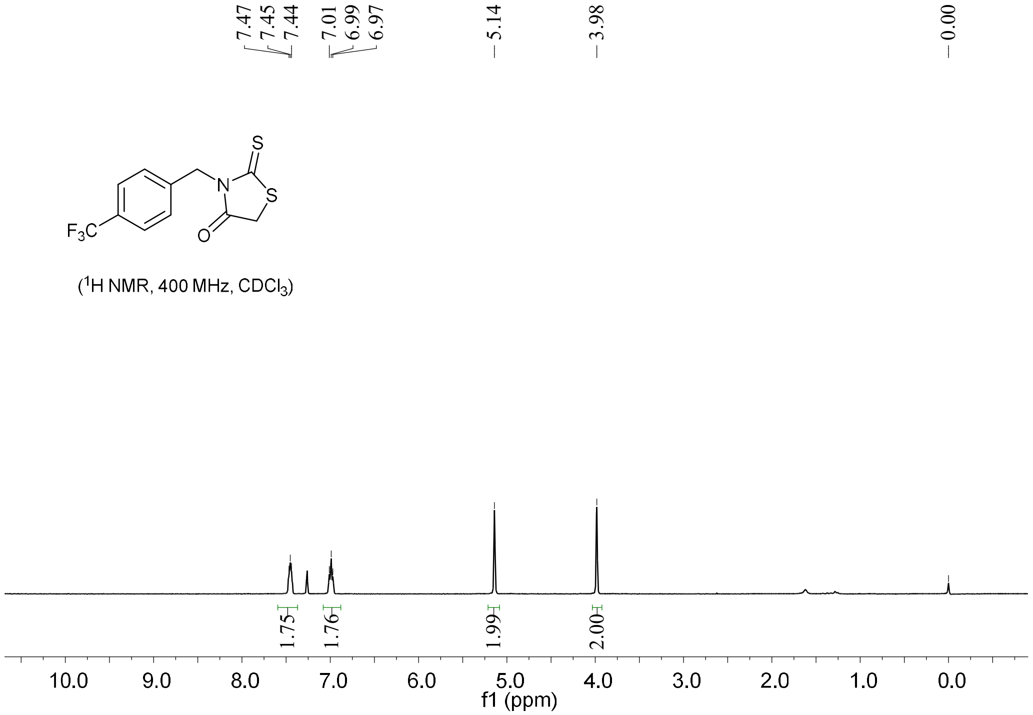

^{1}H NMR of compound **4f**

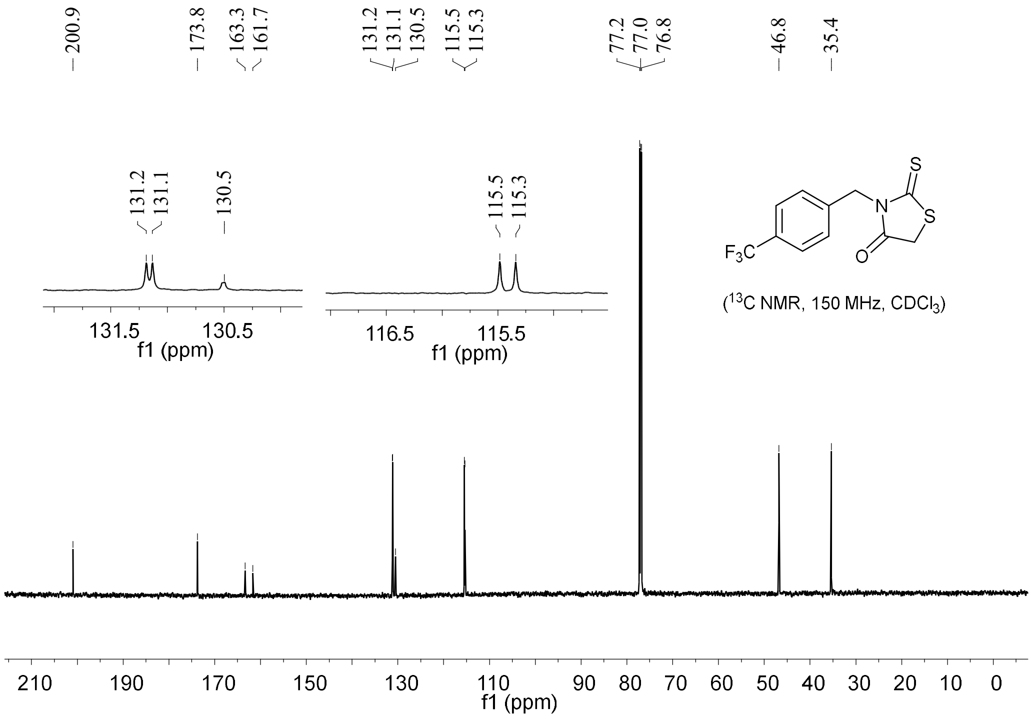

^{13}C NMR of compound **4f**

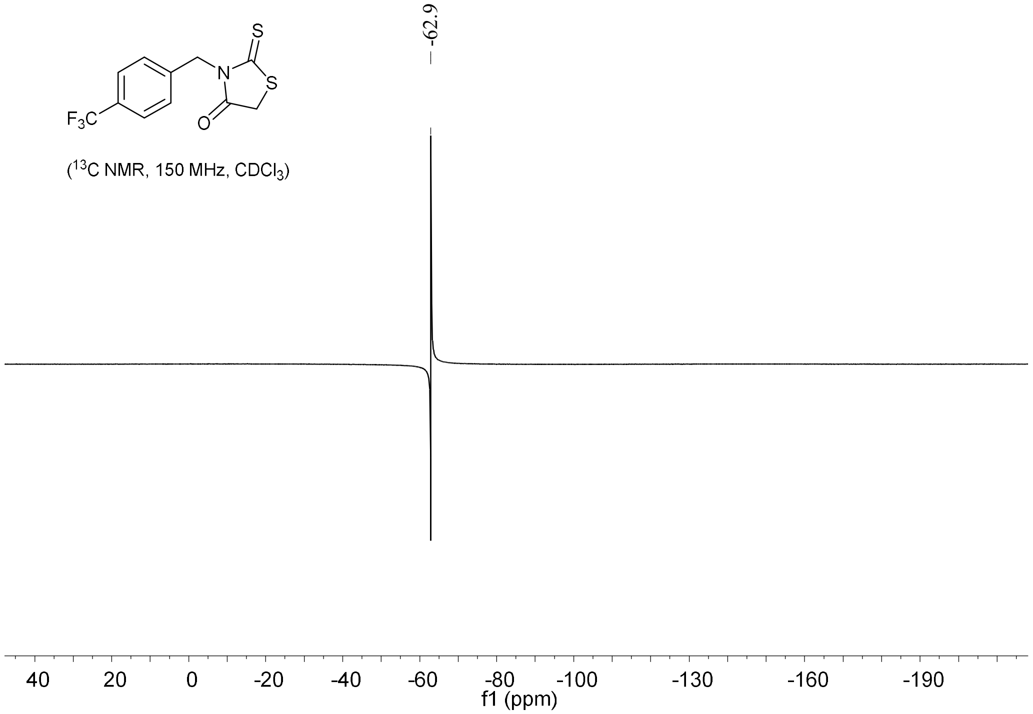

^{19}F NMR of compound **4f**

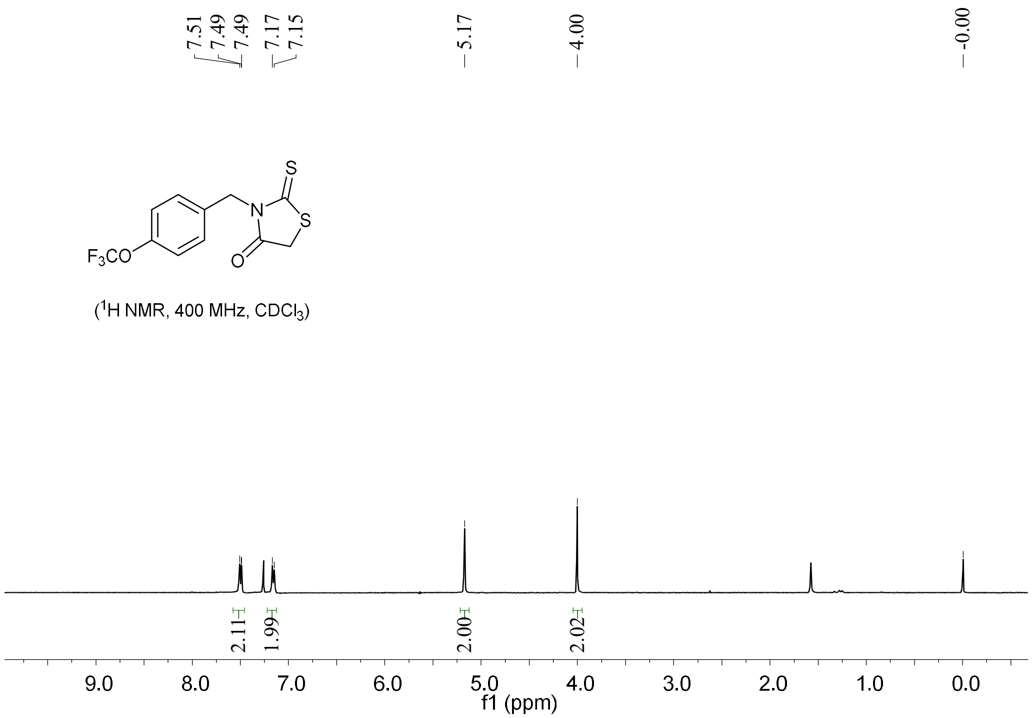

^{1}H NMR of compound **4g**

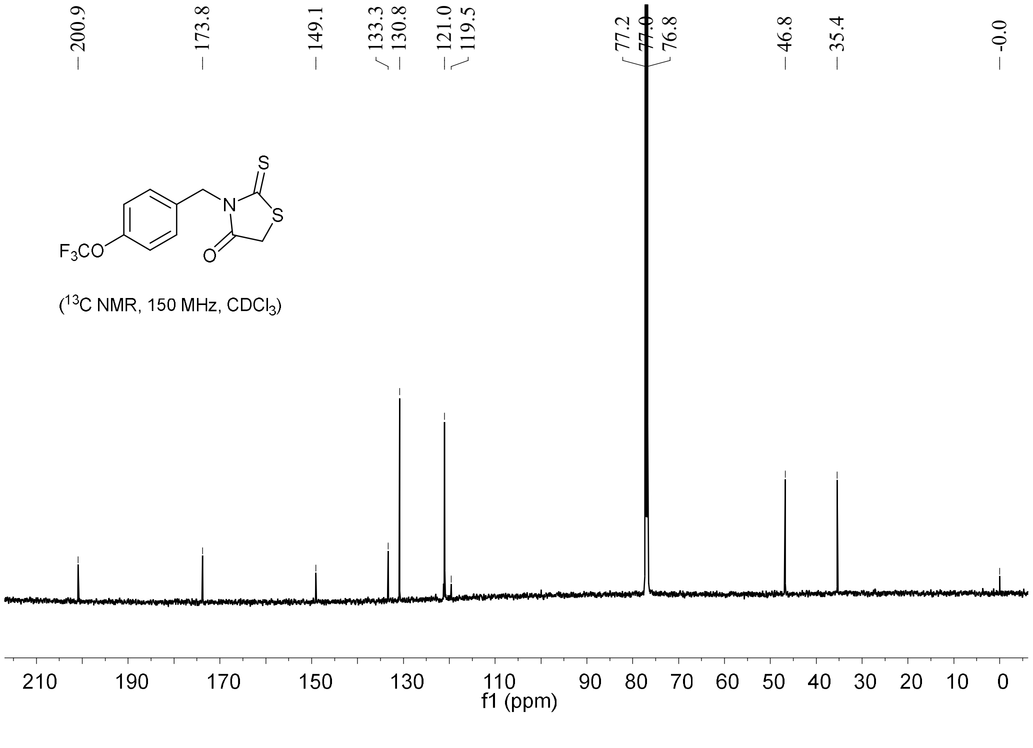

^{13}C NMR of compound **4g**

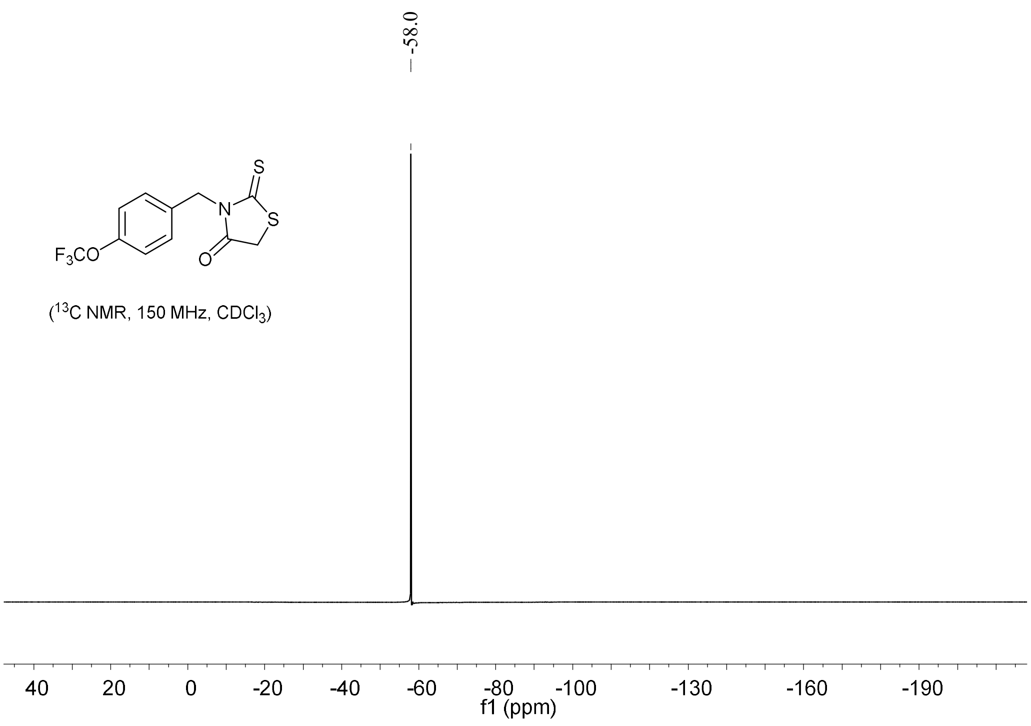

^{19}F NMR of compound **4g**

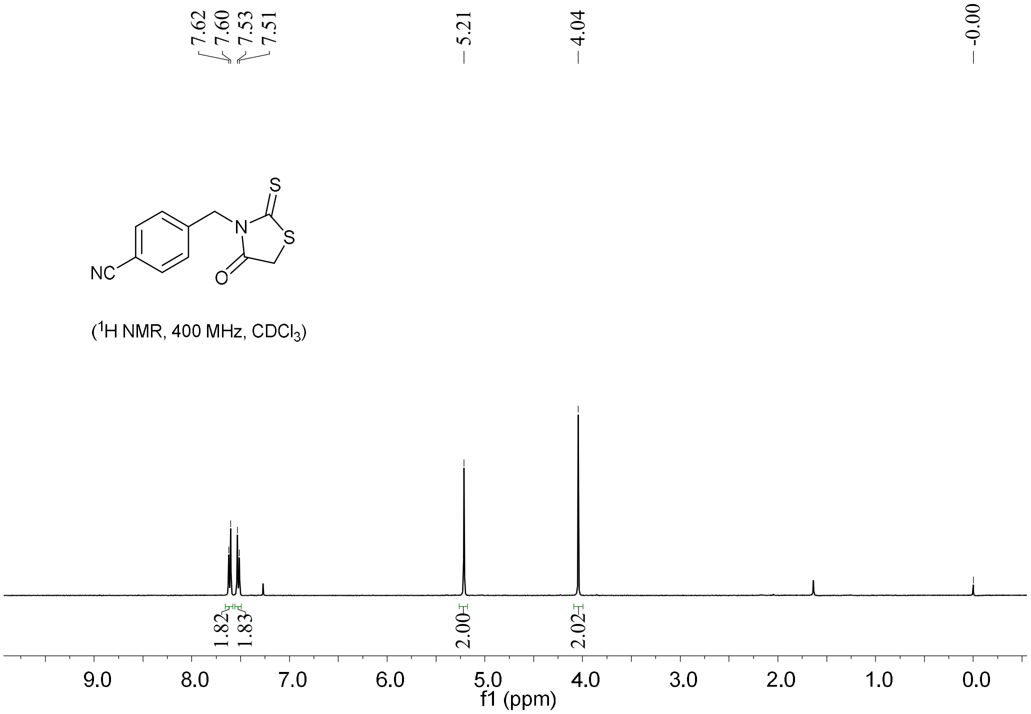

^{1}H NMR of compound **4h**

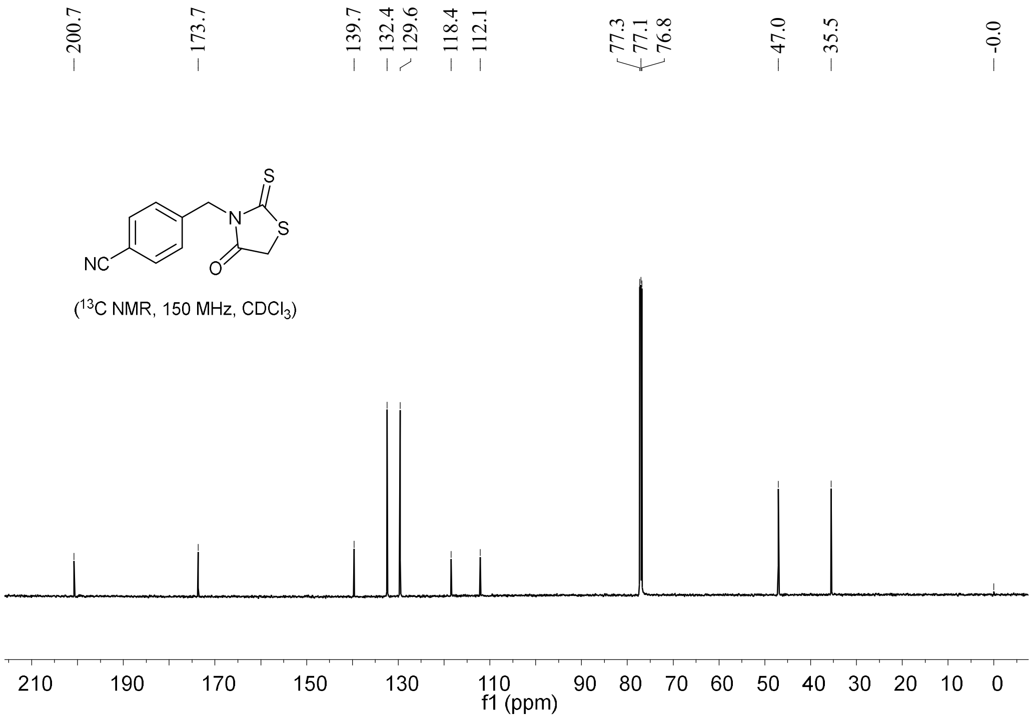

^{13}C NMR of compound **4h**

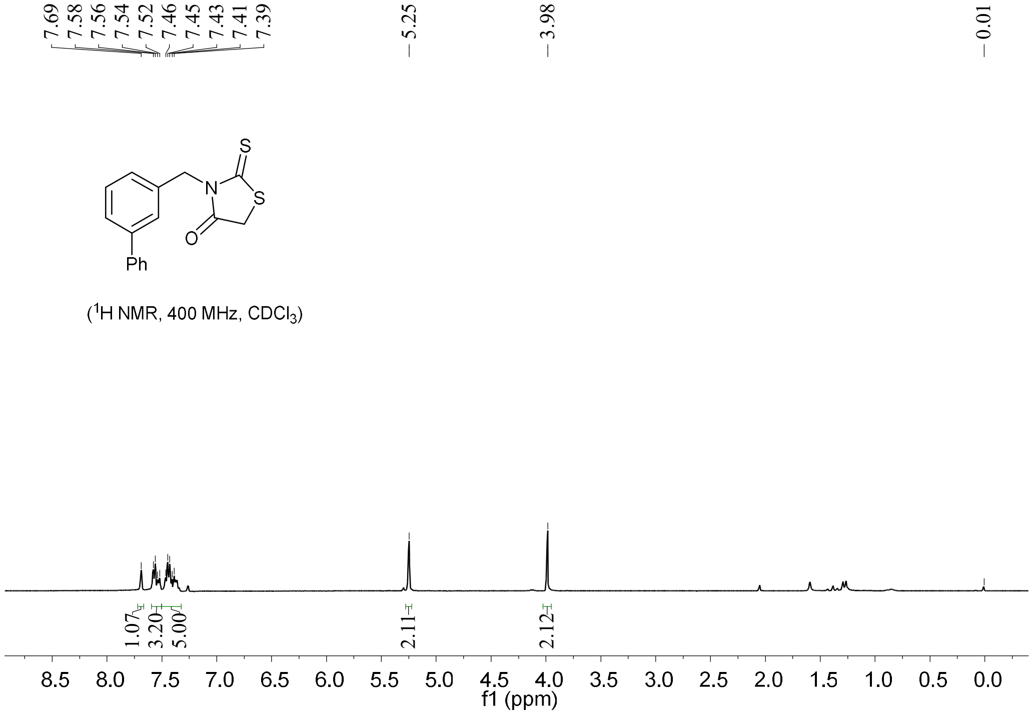

^{1}H NMR of compound **4i**

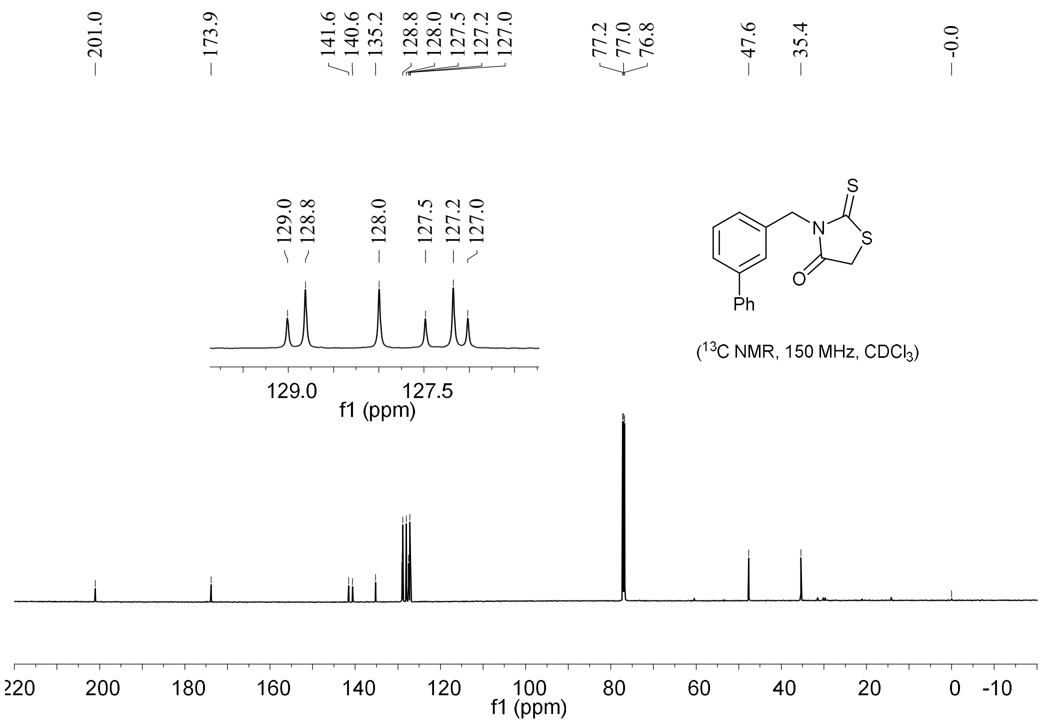

^{13}C NMR NMR of compound **4i**

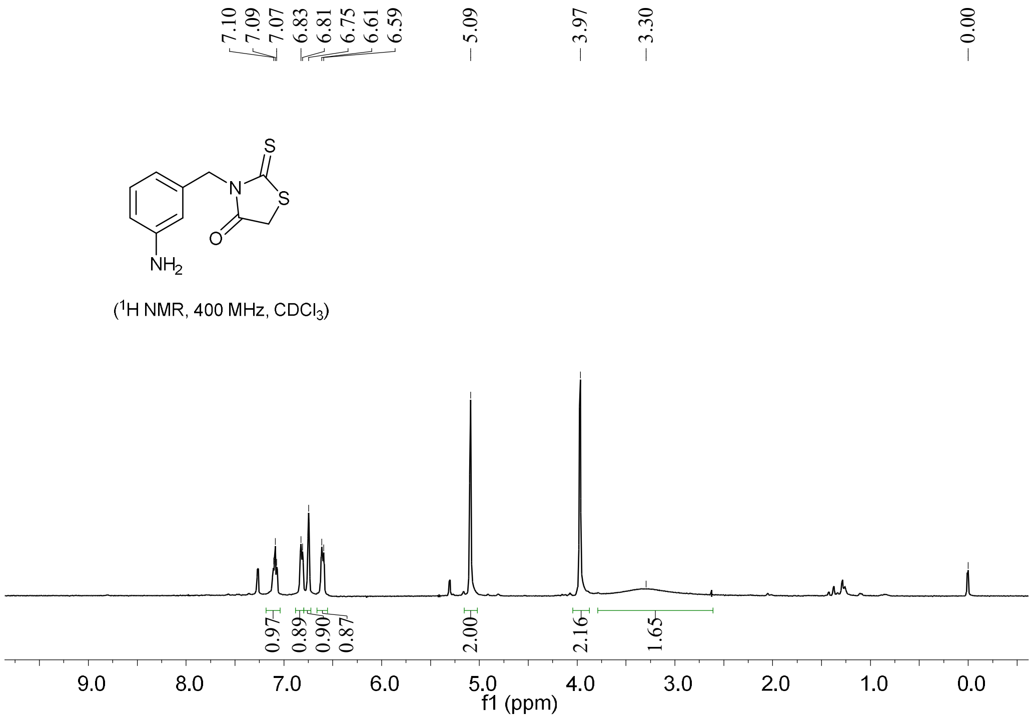

^{1}H NMR of compound **4j**

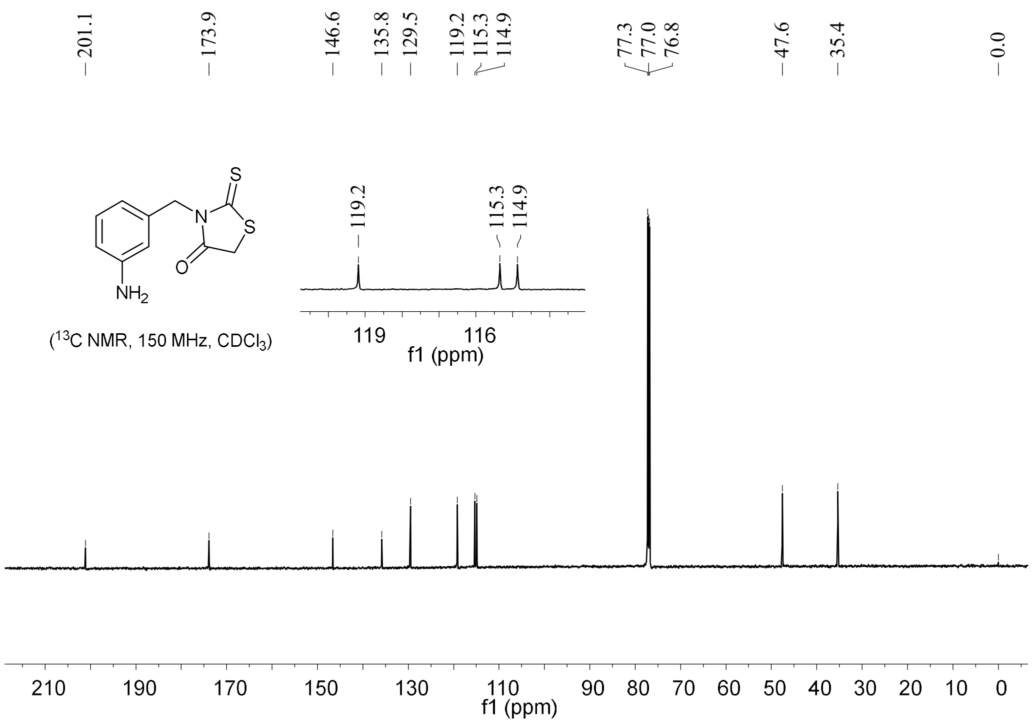

^{13}C NMR of compound **4j**

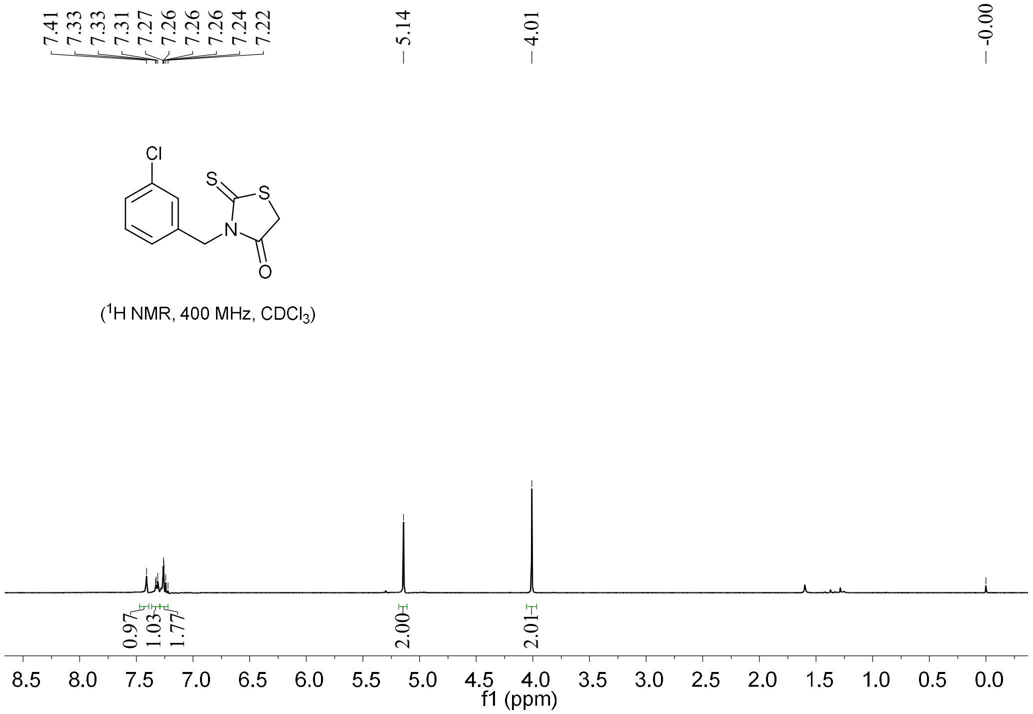

^{1}H NMR of compound **4k**

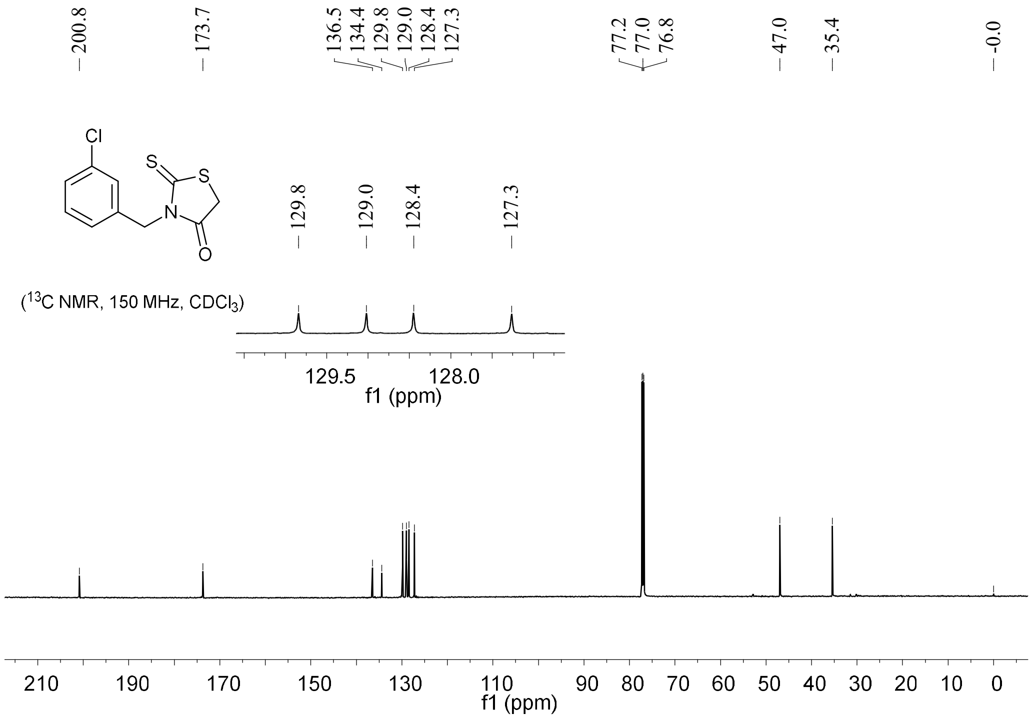

^{13}C NMR of compound **4k**

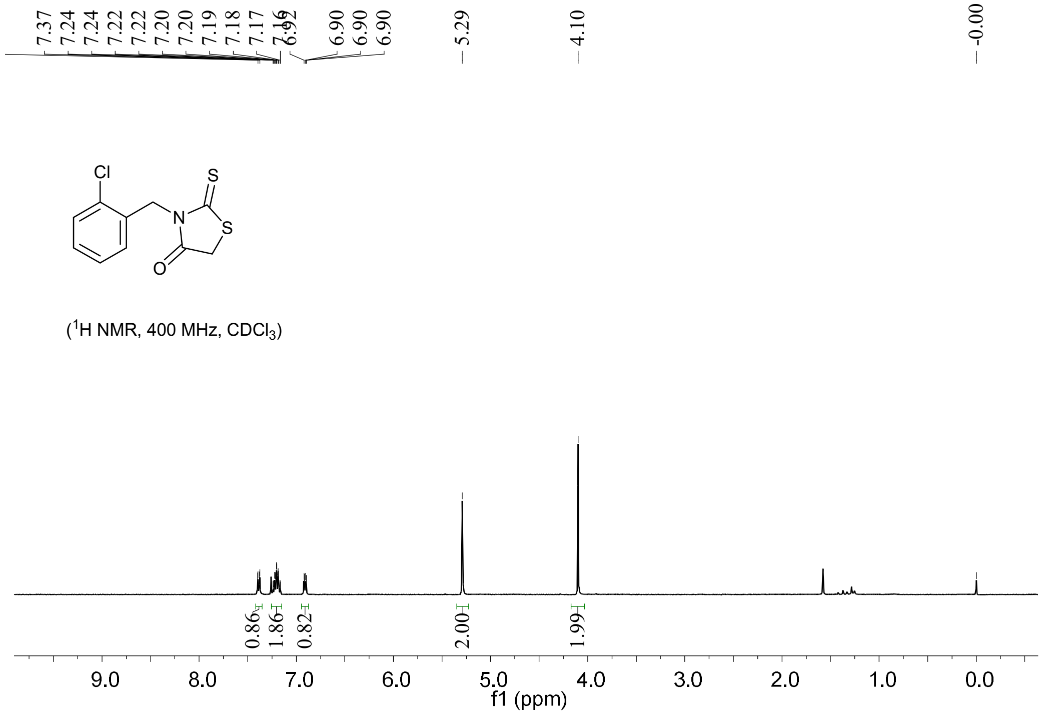

^{1}H NMR of compound **4l**

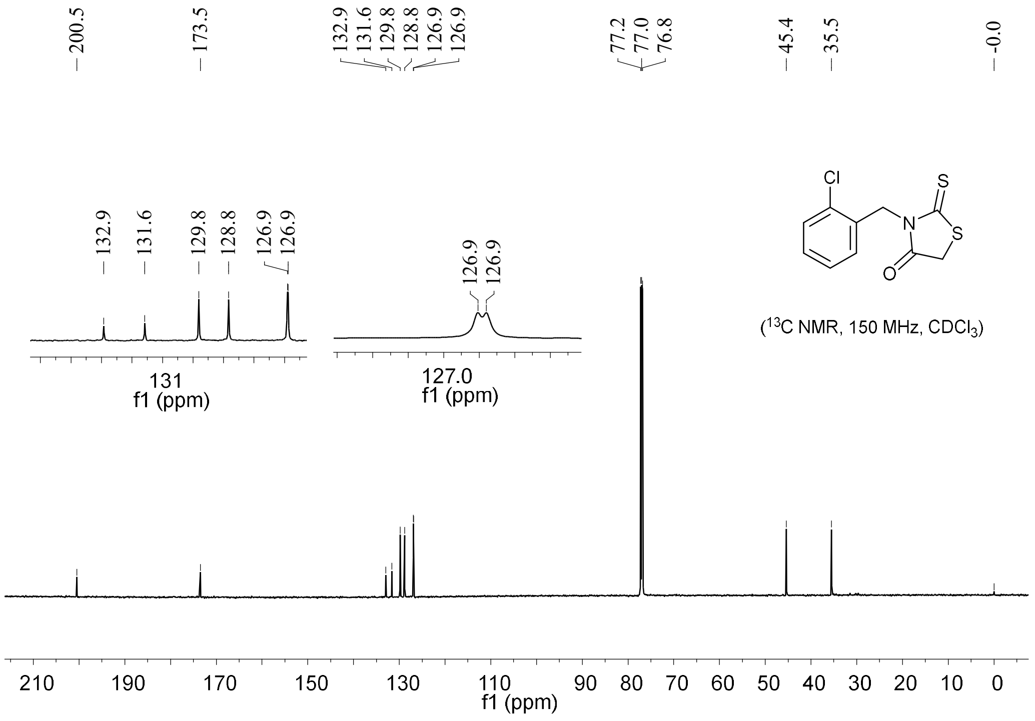

^{13}C NMR of compound **4l**

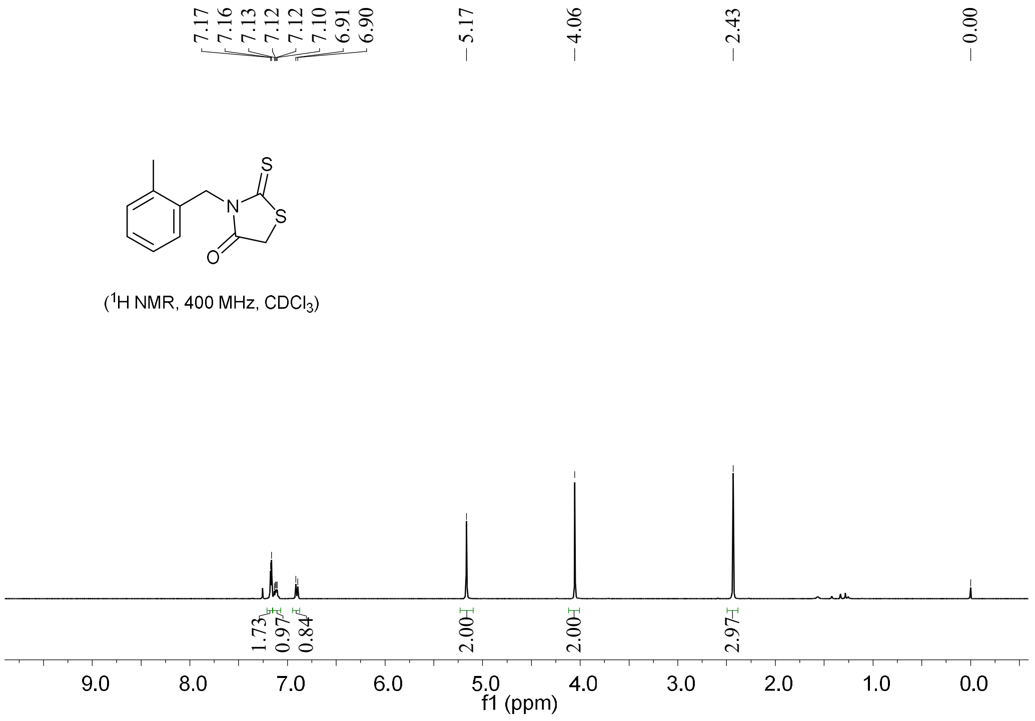

^{1}H NMR of compound **4m**

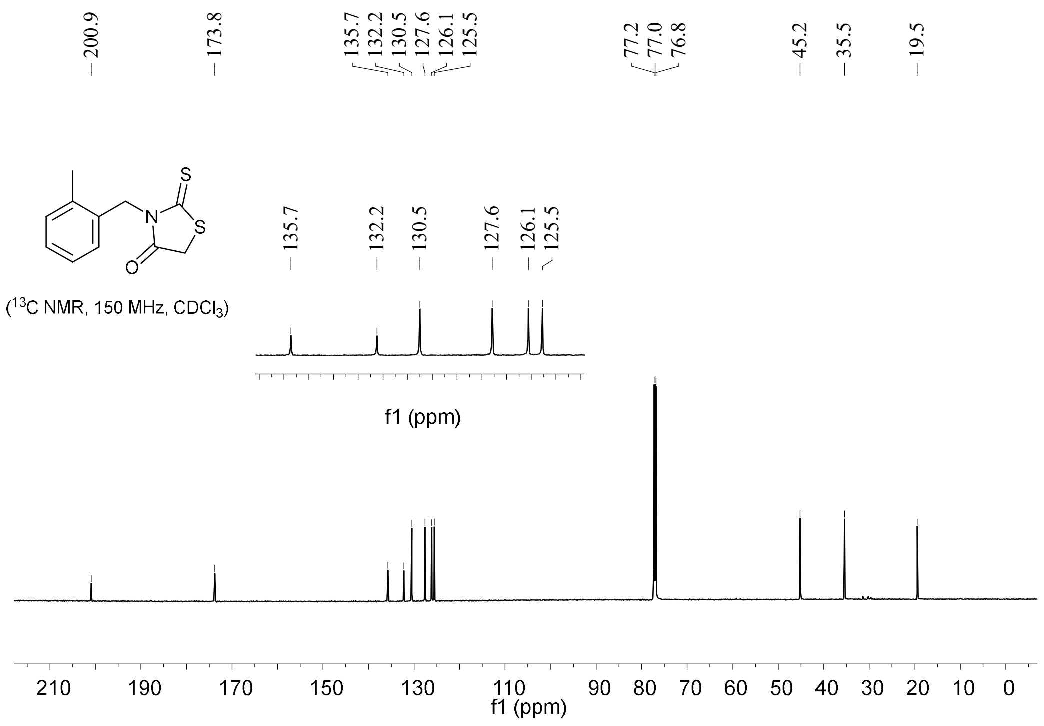

^{13}C NMR of compound **4m**

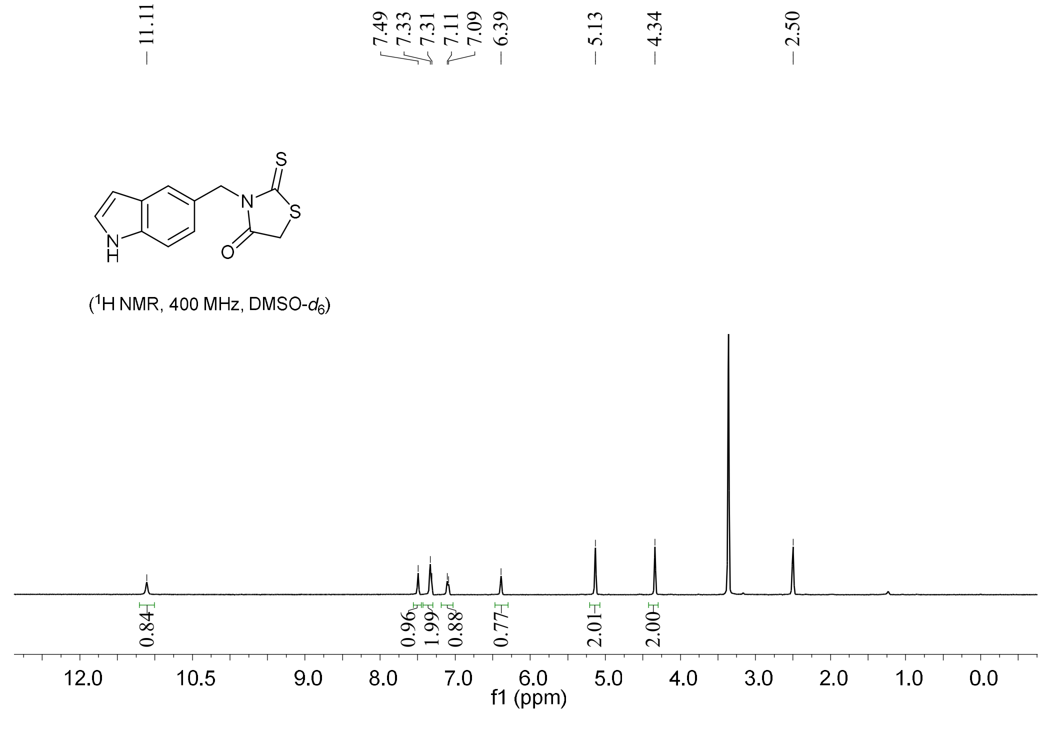

^{1}H NMR of compound **4n**

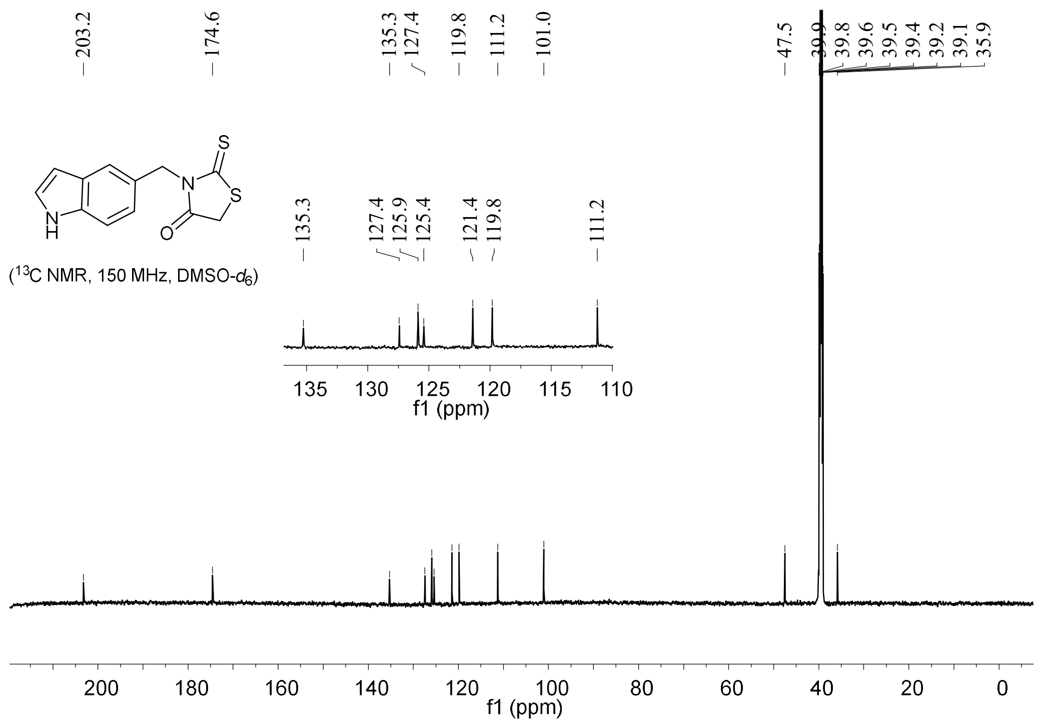

^{13}C NMR NMR of compound **4n**

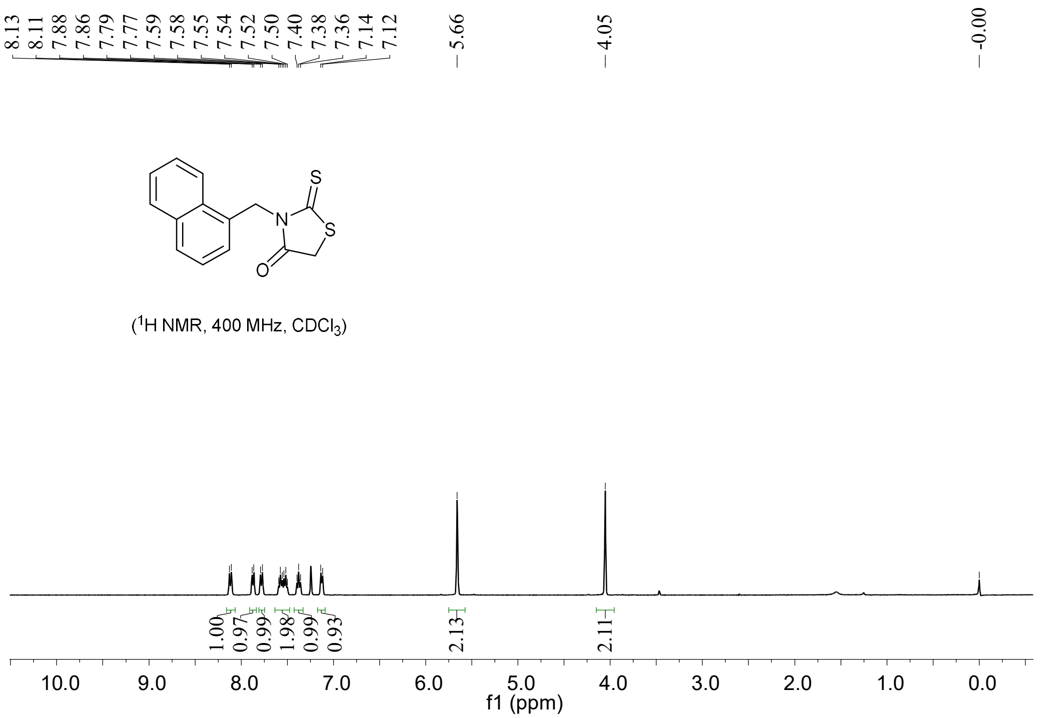

^{1}H NMR of compound **4o**

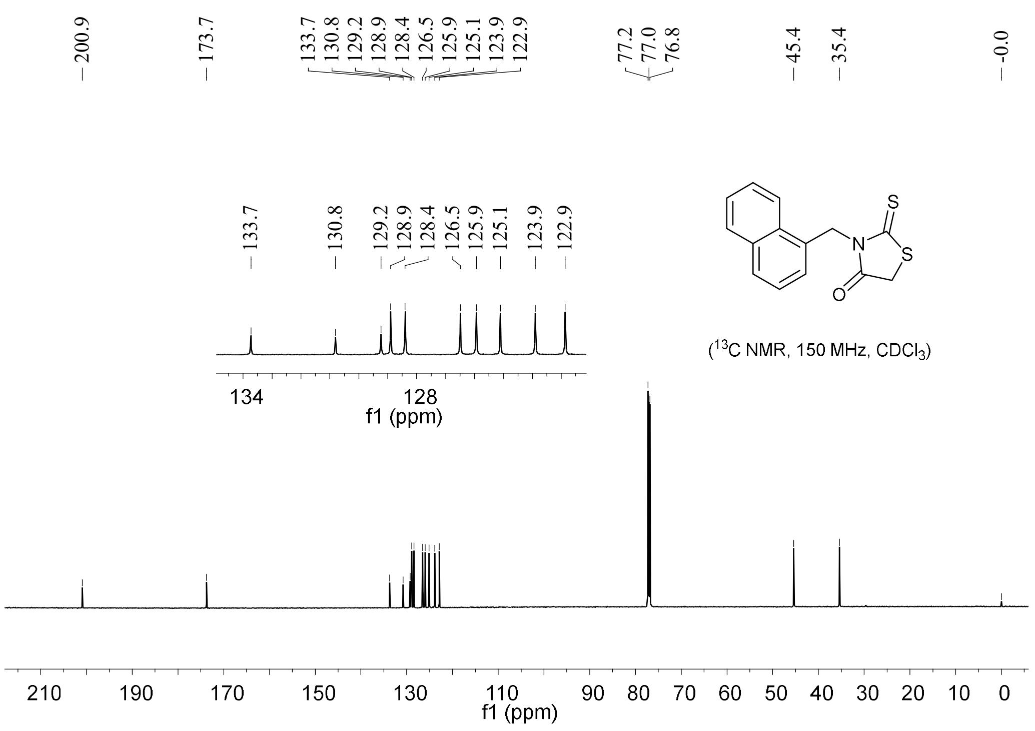

^{13}C NMR NMR of compound **4o**

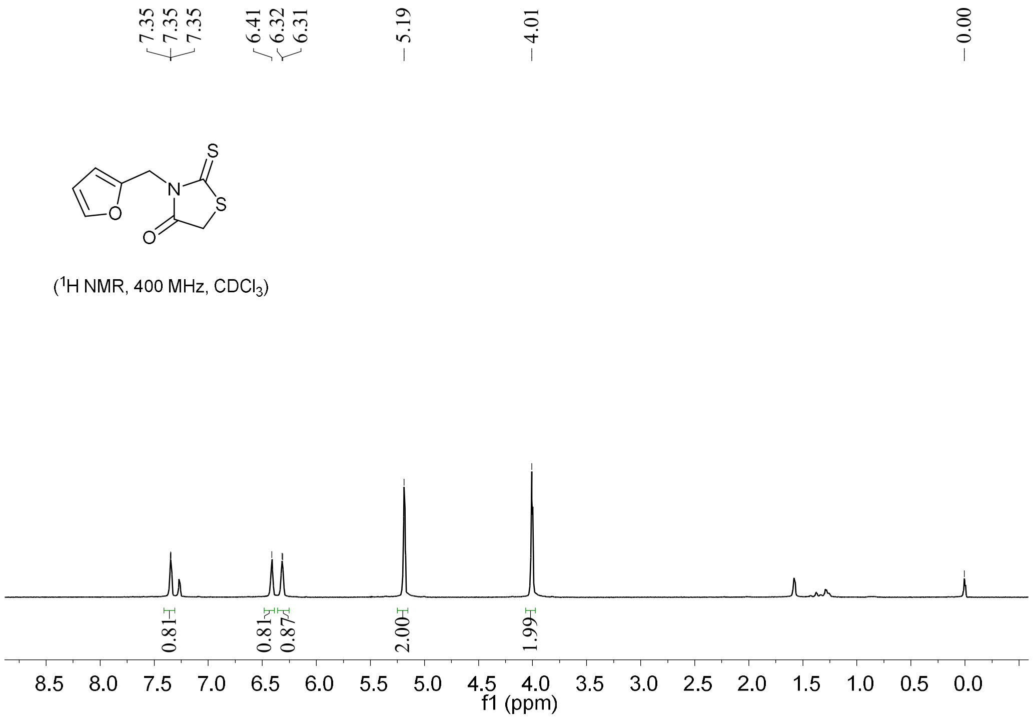

^{1}H NMR of compound **4p**

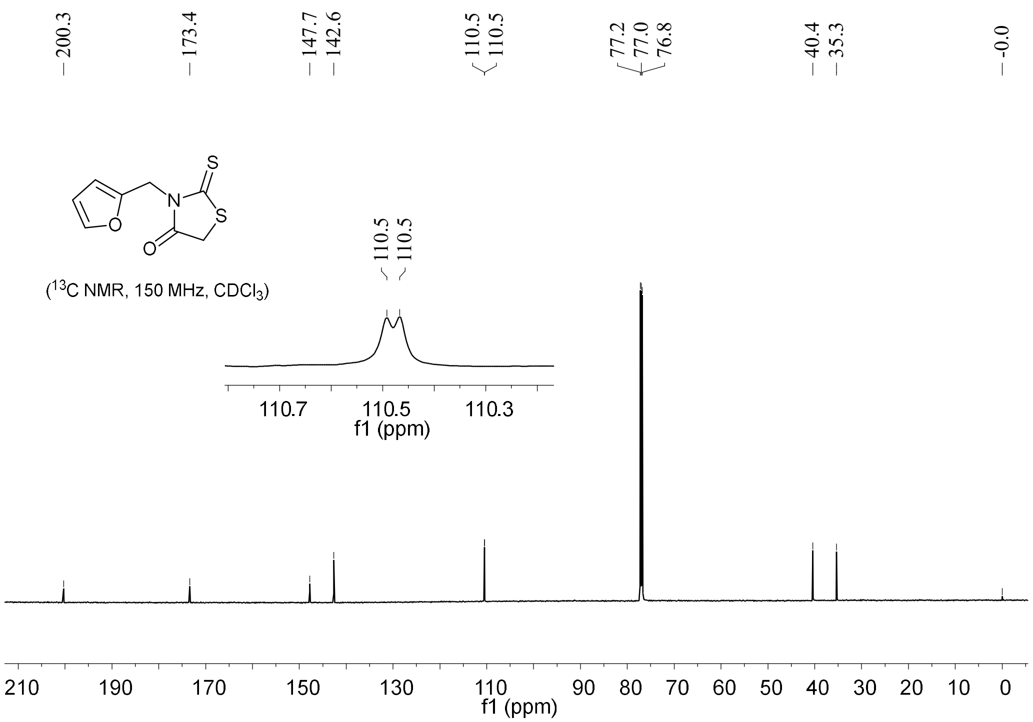

^{13}C NMR of compound **4p**

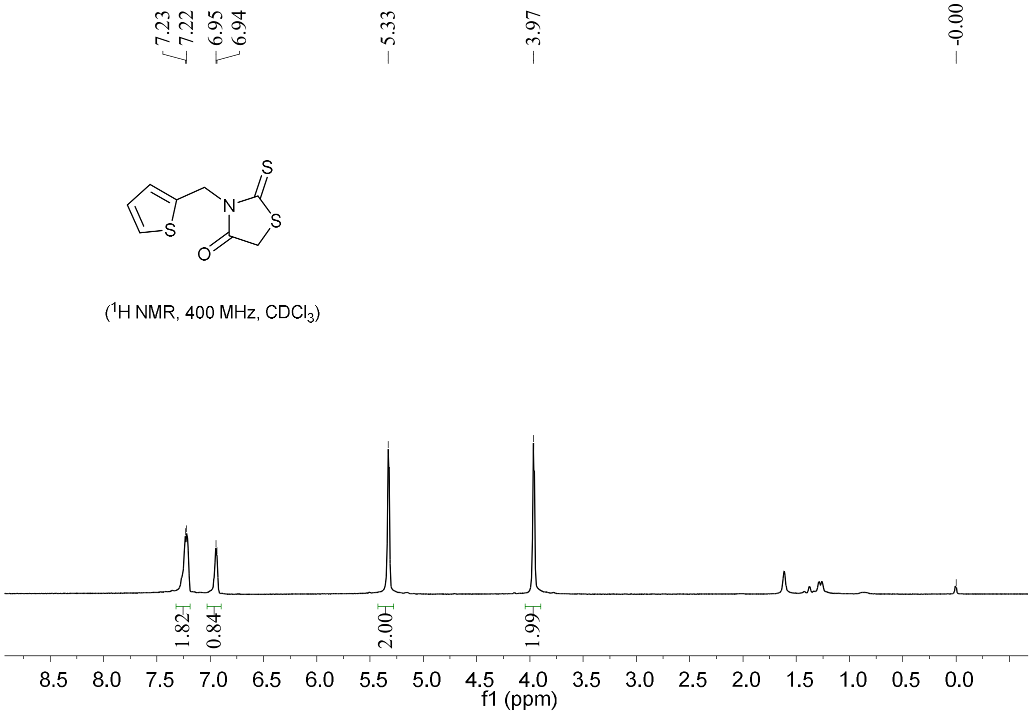

^{1}H NMR of compound **4q**

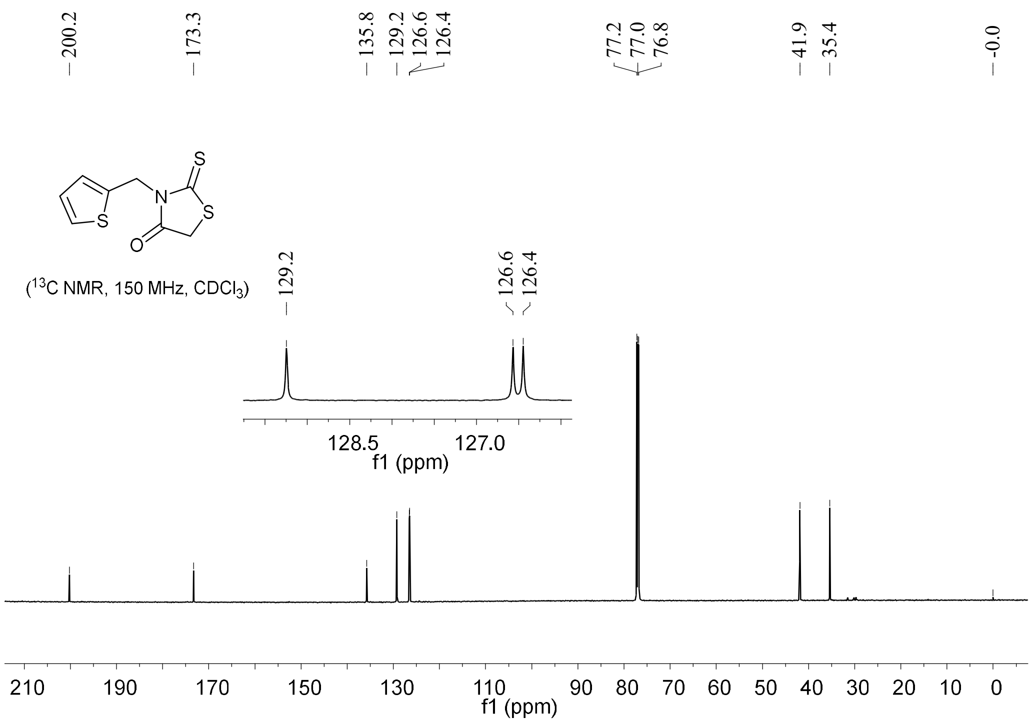

^{13}C NMR of compound **4q**

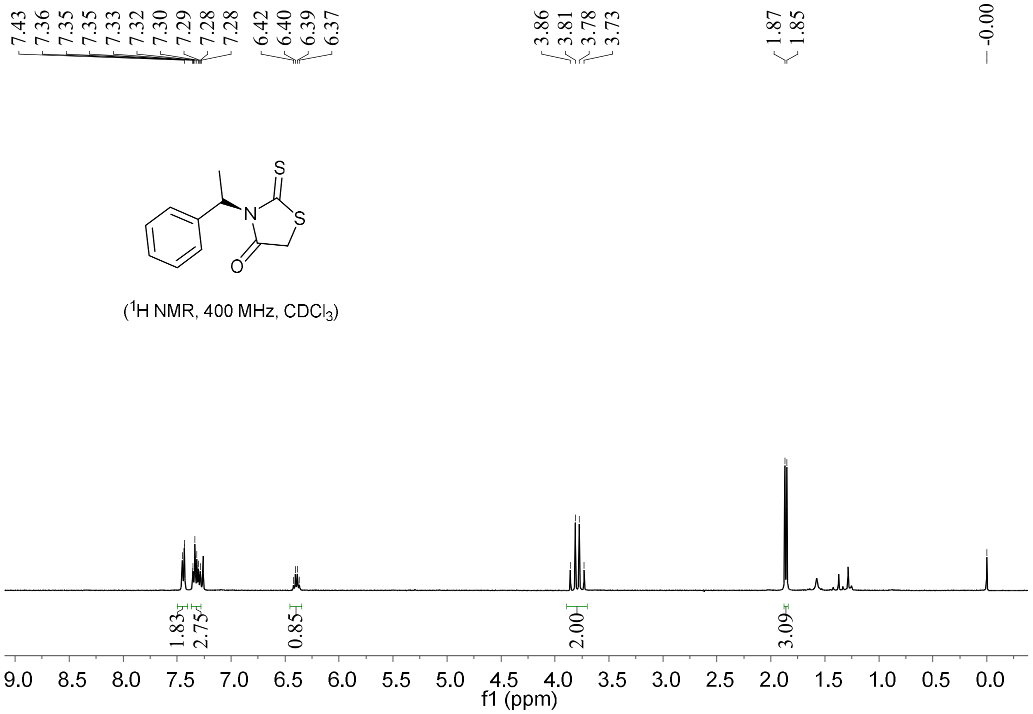

^{1}H NMR of compound **4r**

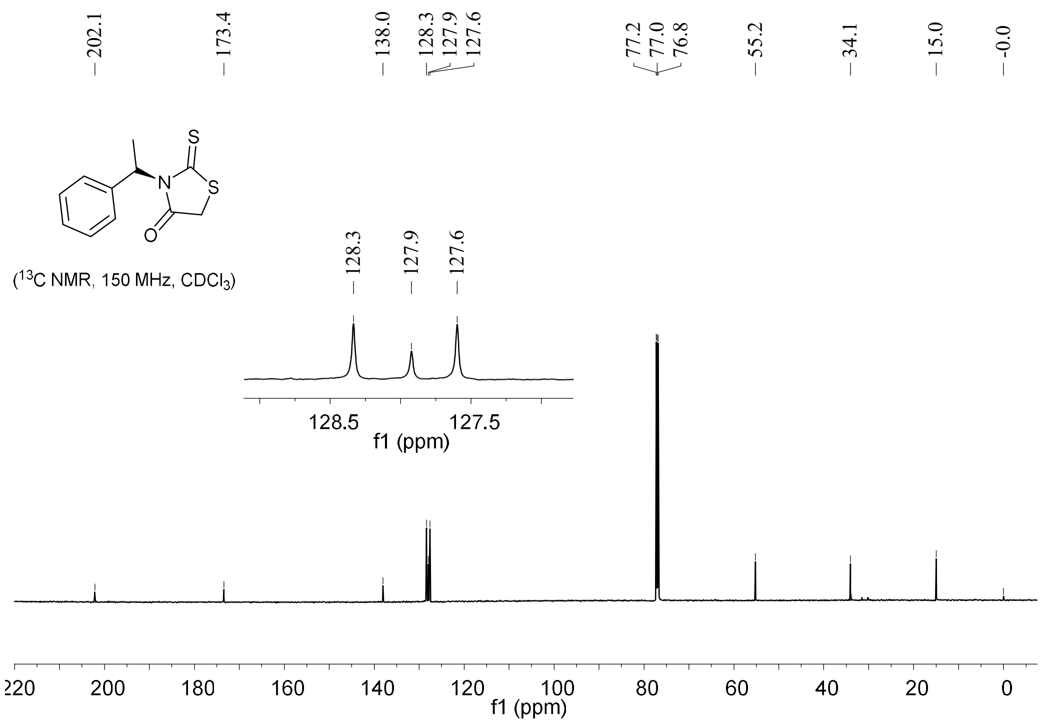

^{13}C NMR of compound **4r**

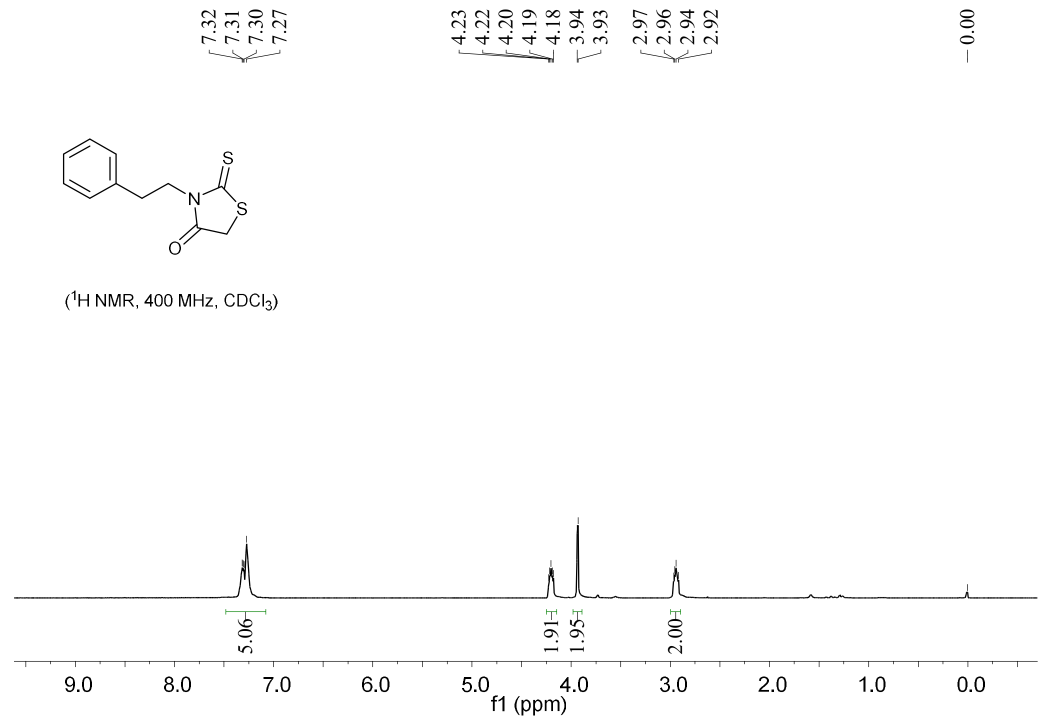

^{1}H NMR of compound **4s**

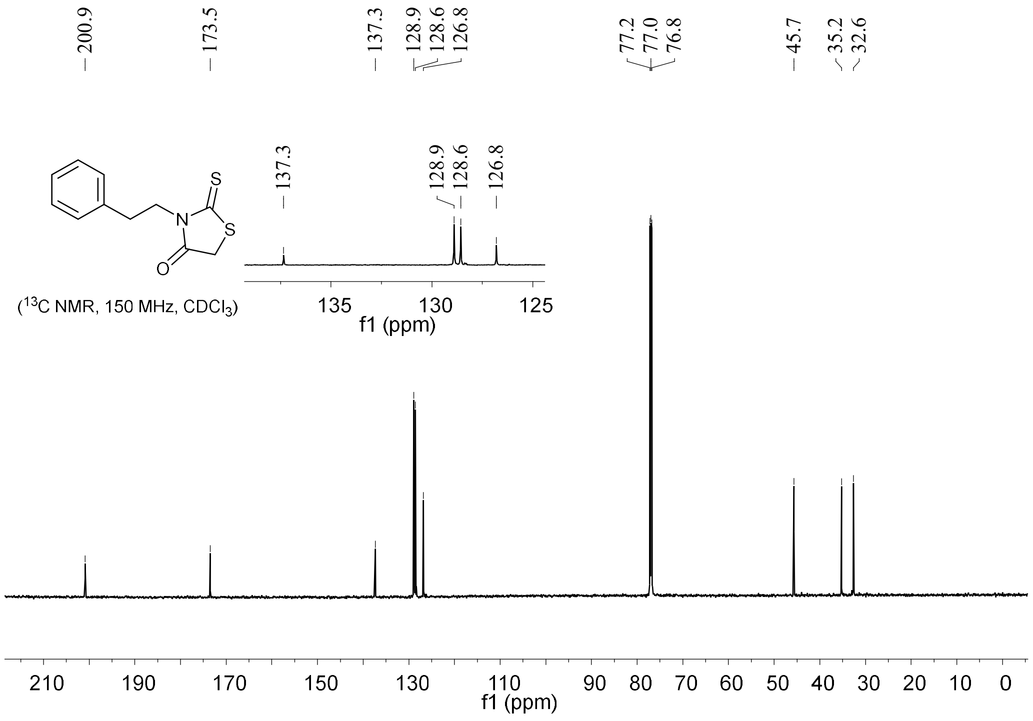

^{13}C NMR of compound **4s**

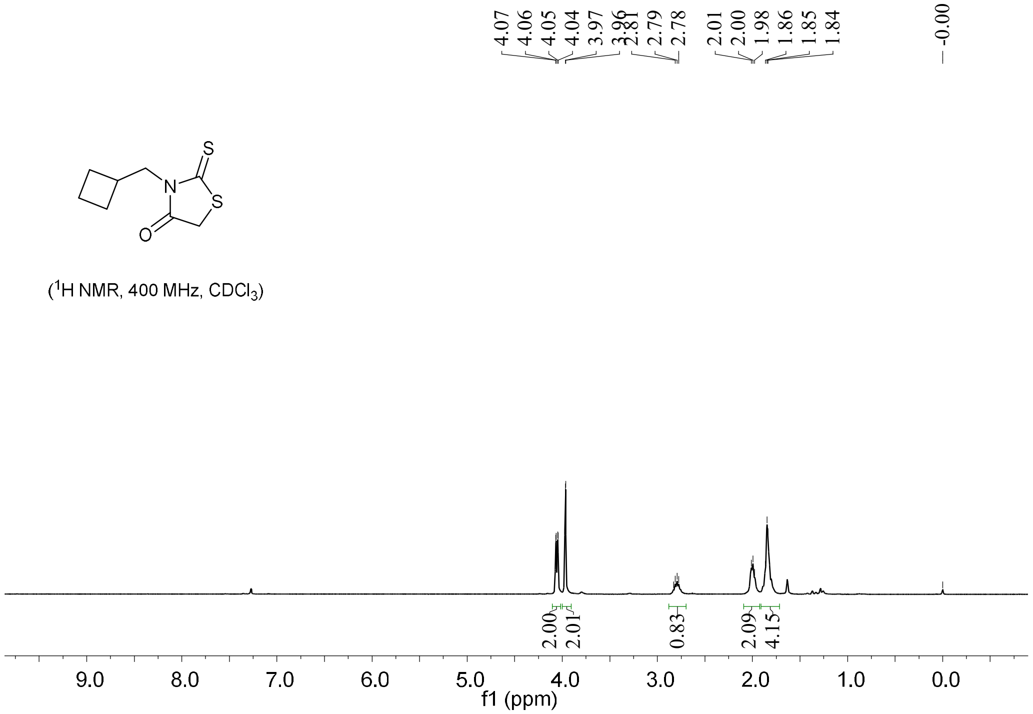

^{1}H NMR of compound **4t**

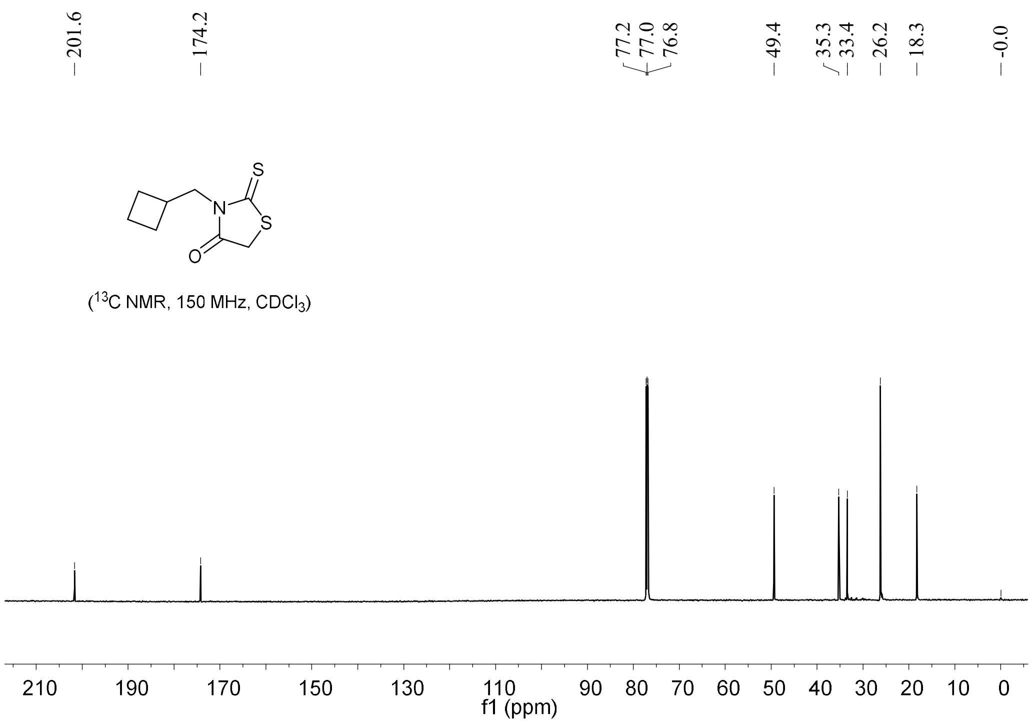

^{13}C NMR of compound **4t**

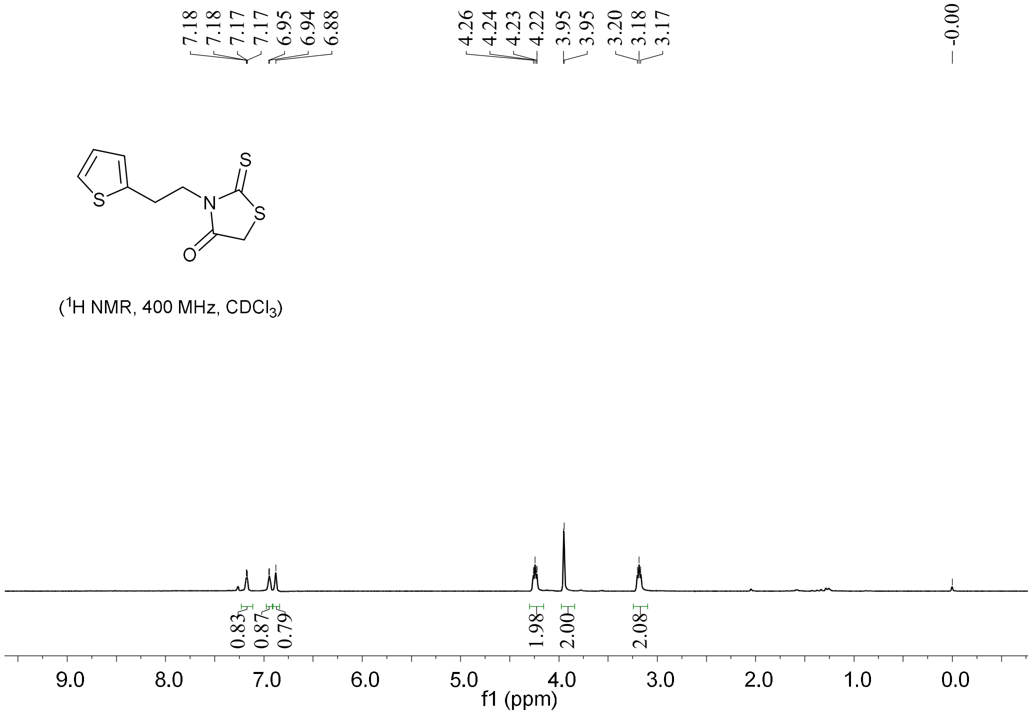

^{1}H NMR of compound **4u**

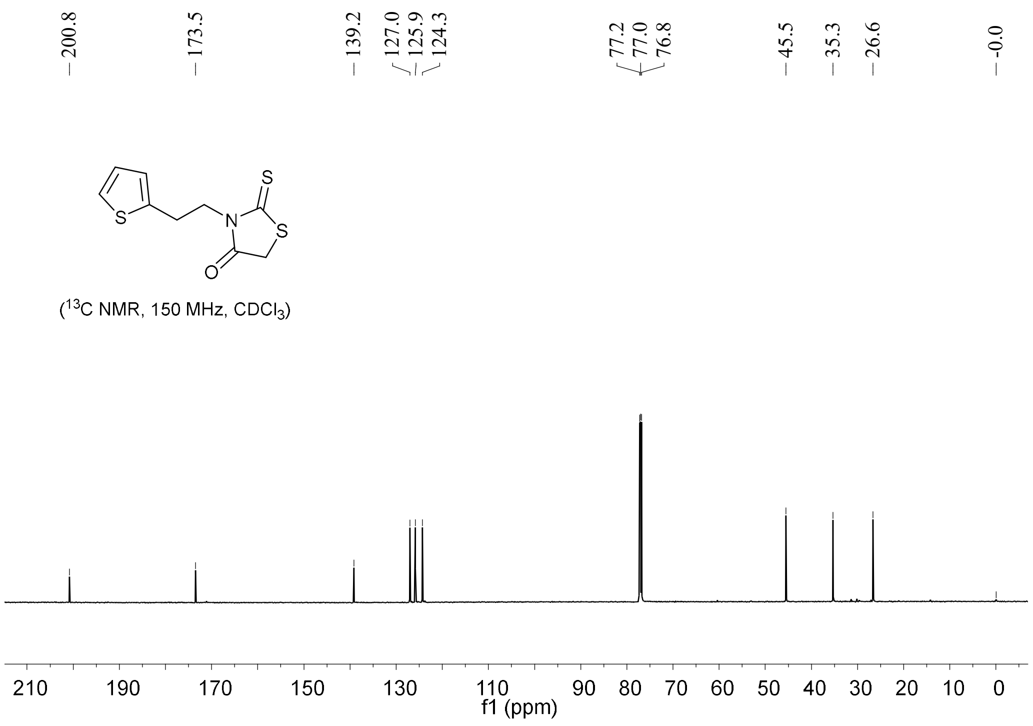

^{13}C NMR of compound **4u**

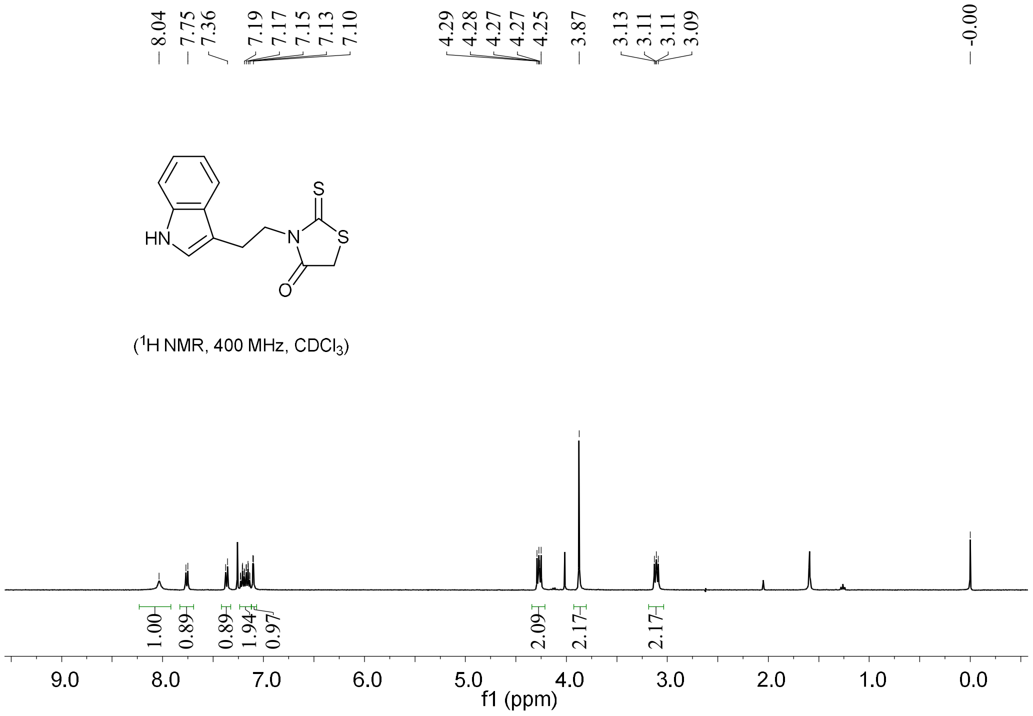

^{1}H NMR of compound **4v**

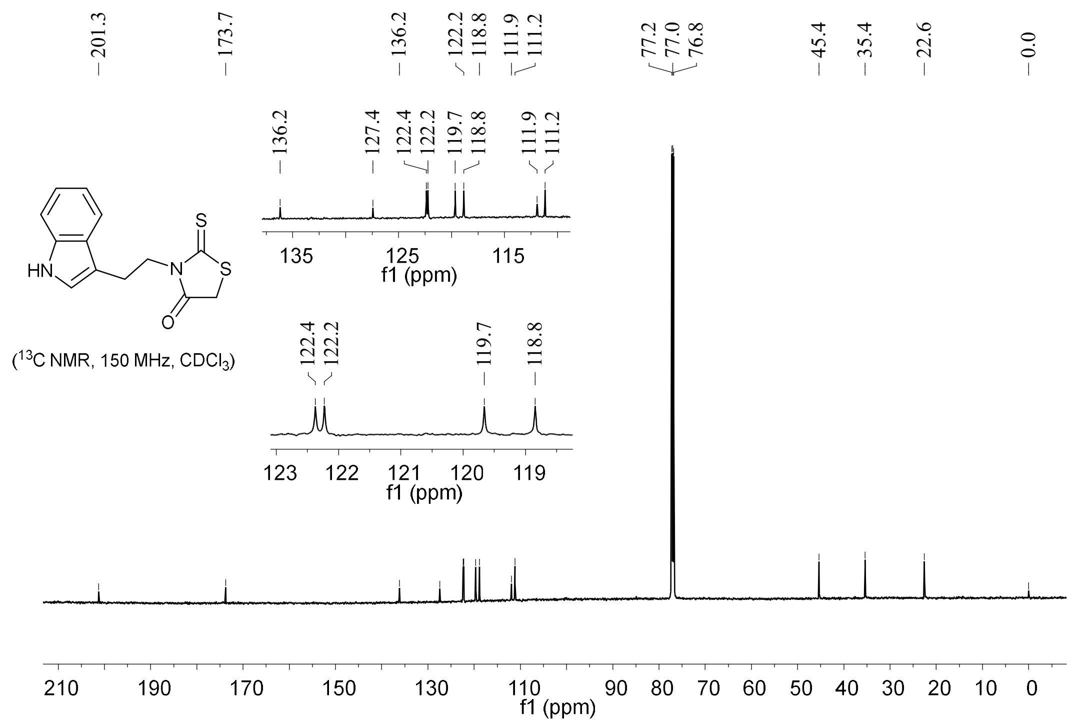

^{13}C NMR of compound **4v**

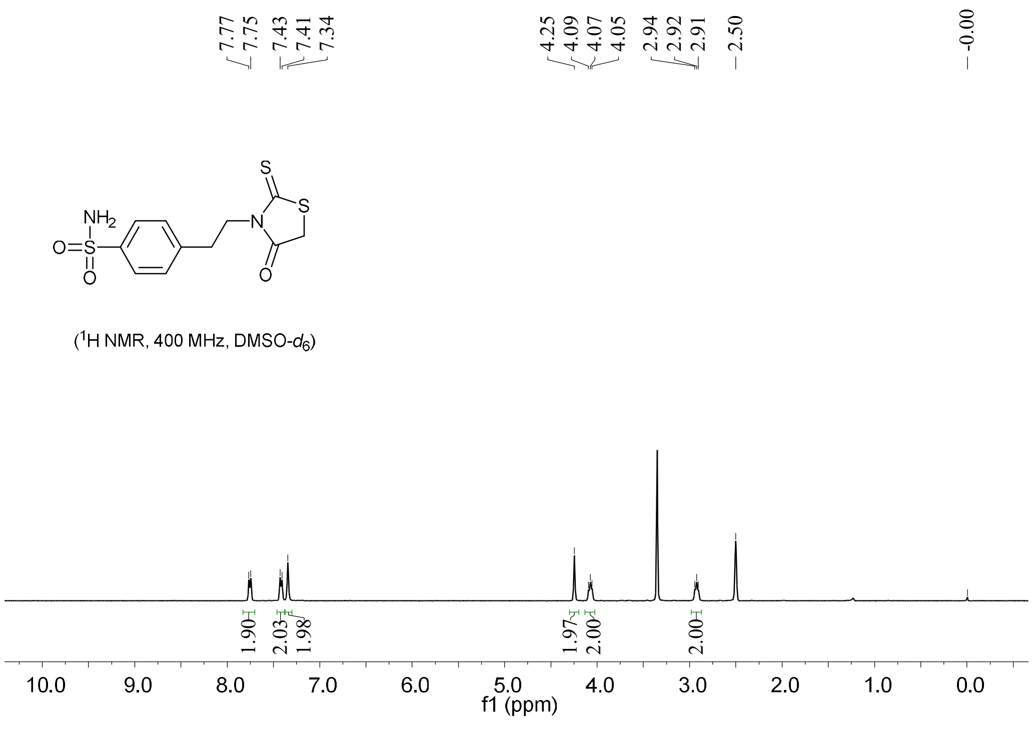

^{1}H NMR of compound **4w**

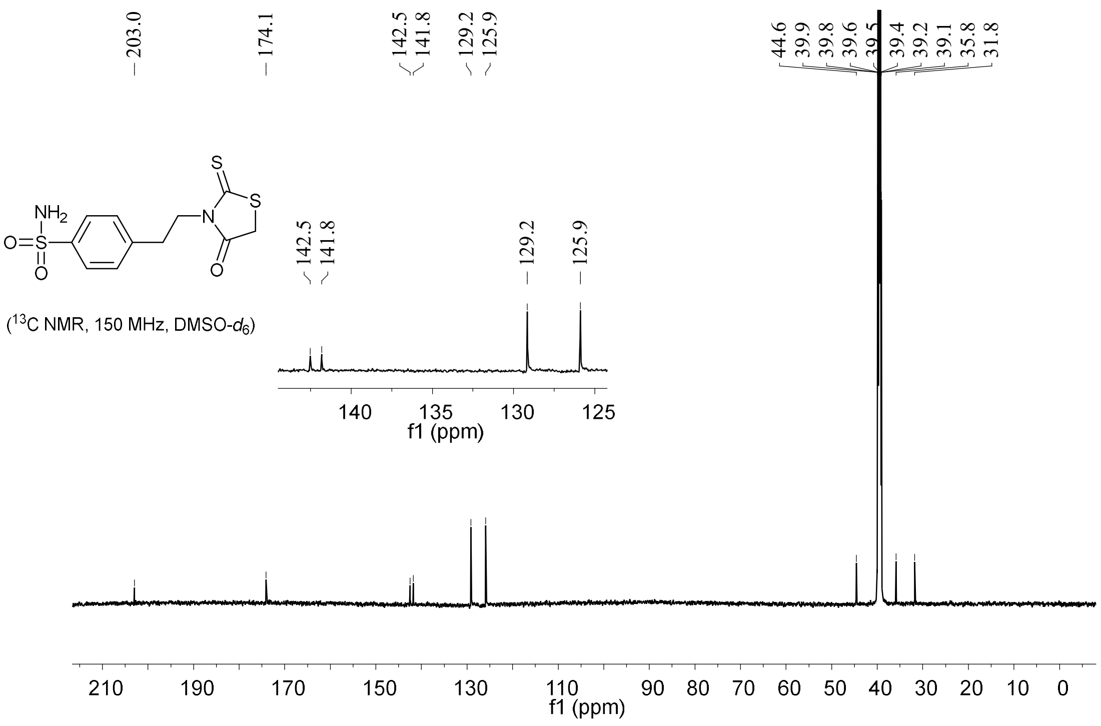

^{13}C NMR of compound **4w**

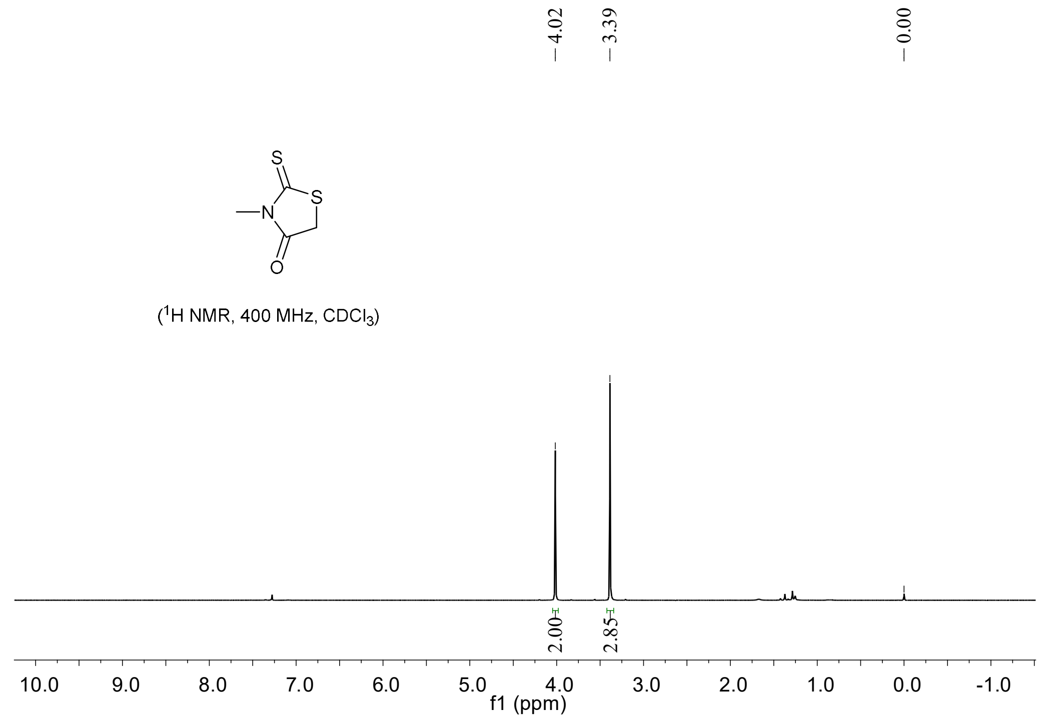

^{1}H NMR of compound **4x**

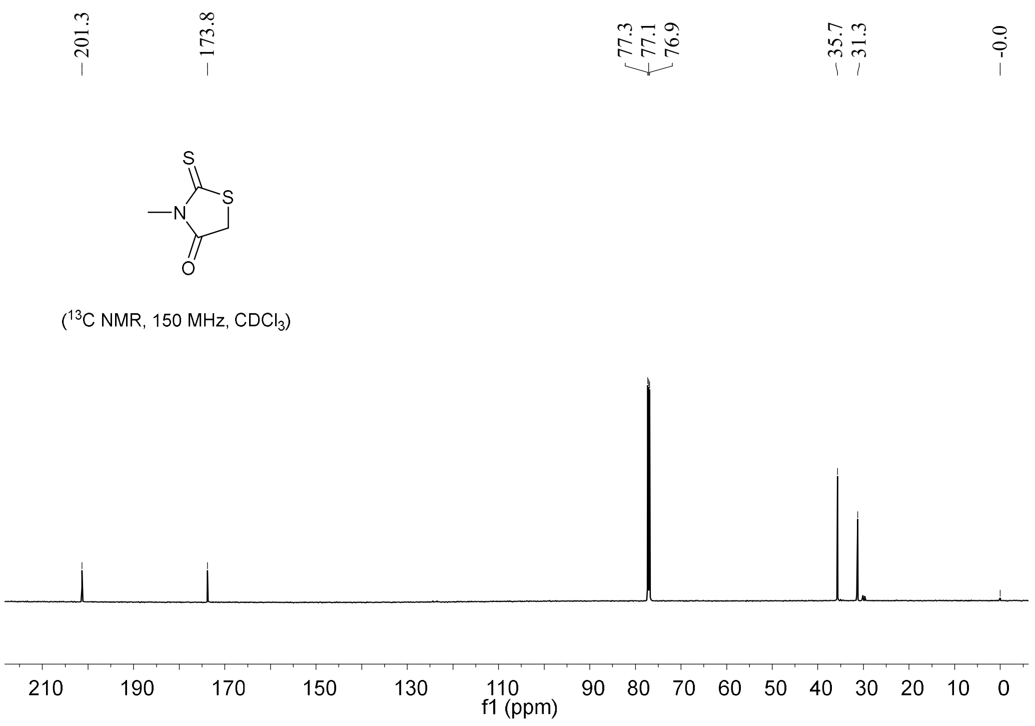

^{13}C NMR of compound **4x**

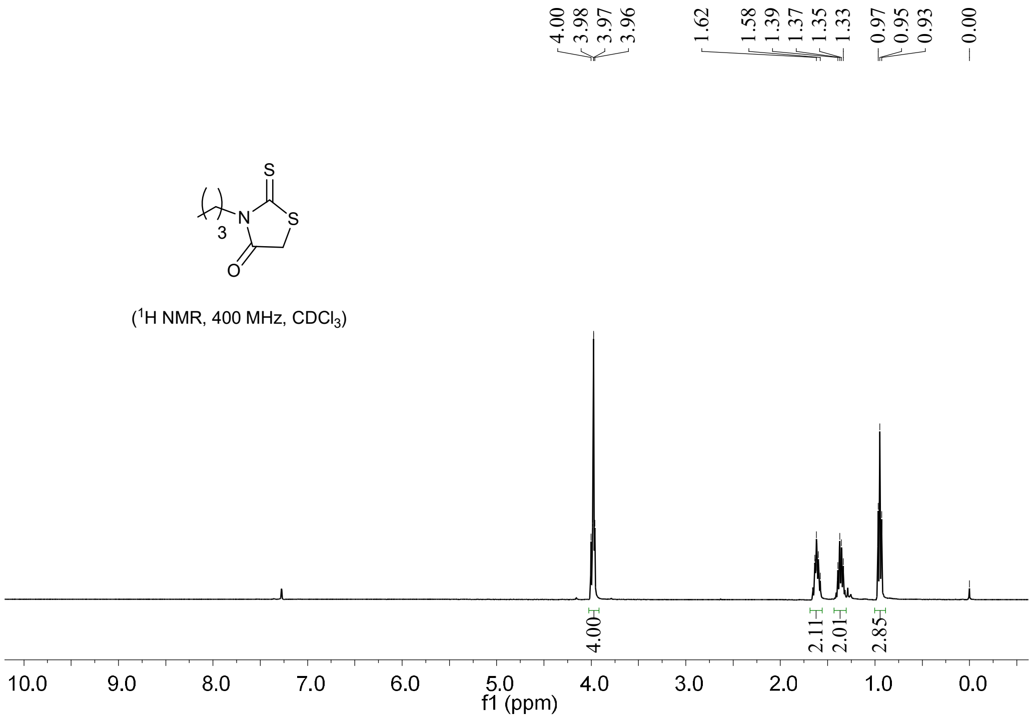

^{1}H NMR of compound **4y**

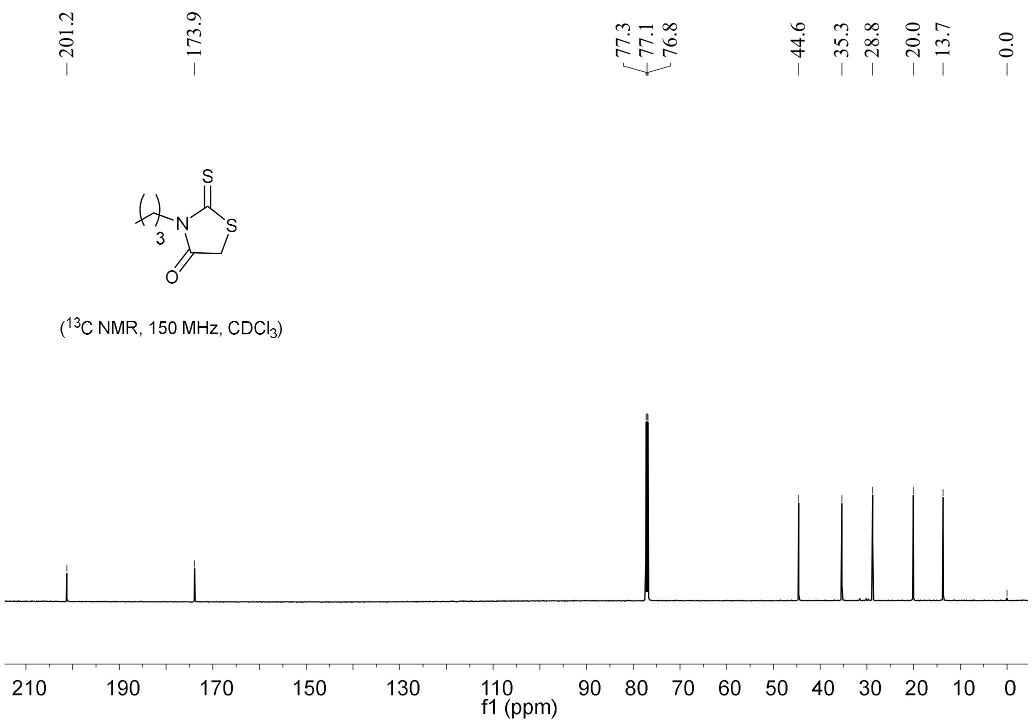

^{13}C NMR of compound **4y**

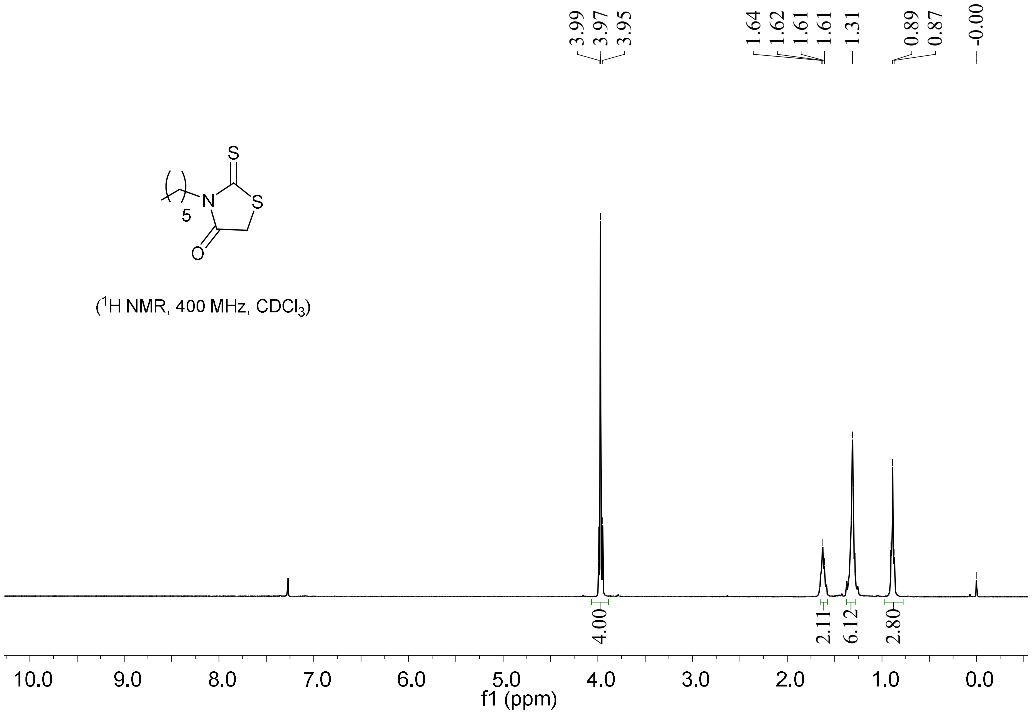

^{1}H NMR of compound **4z**

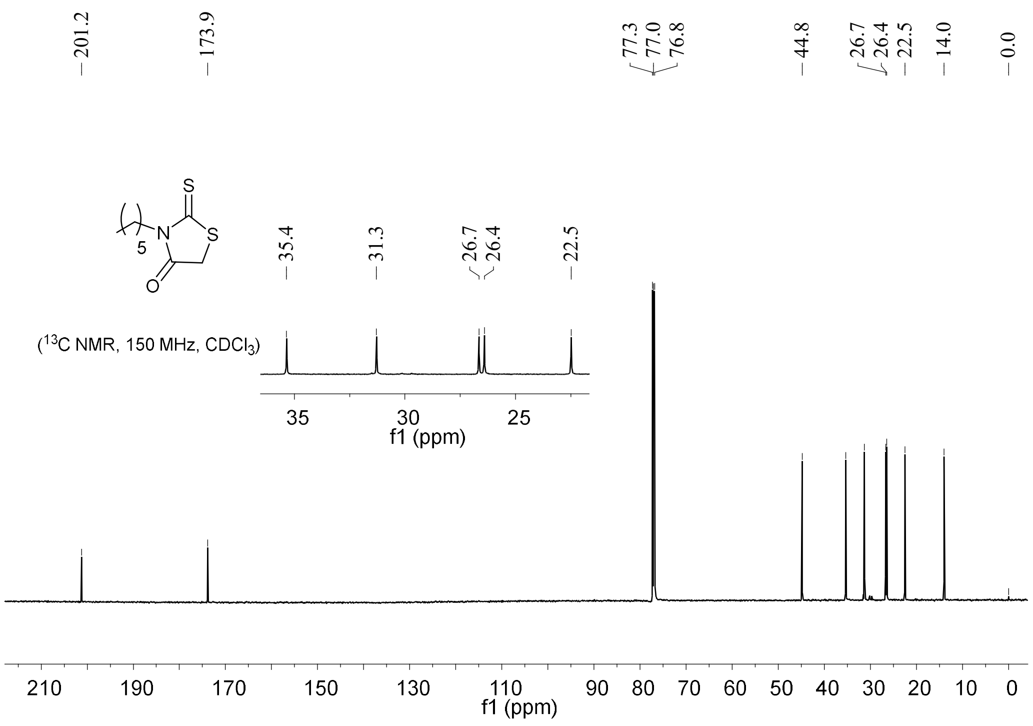

^{13}C NMR of compound **4z**

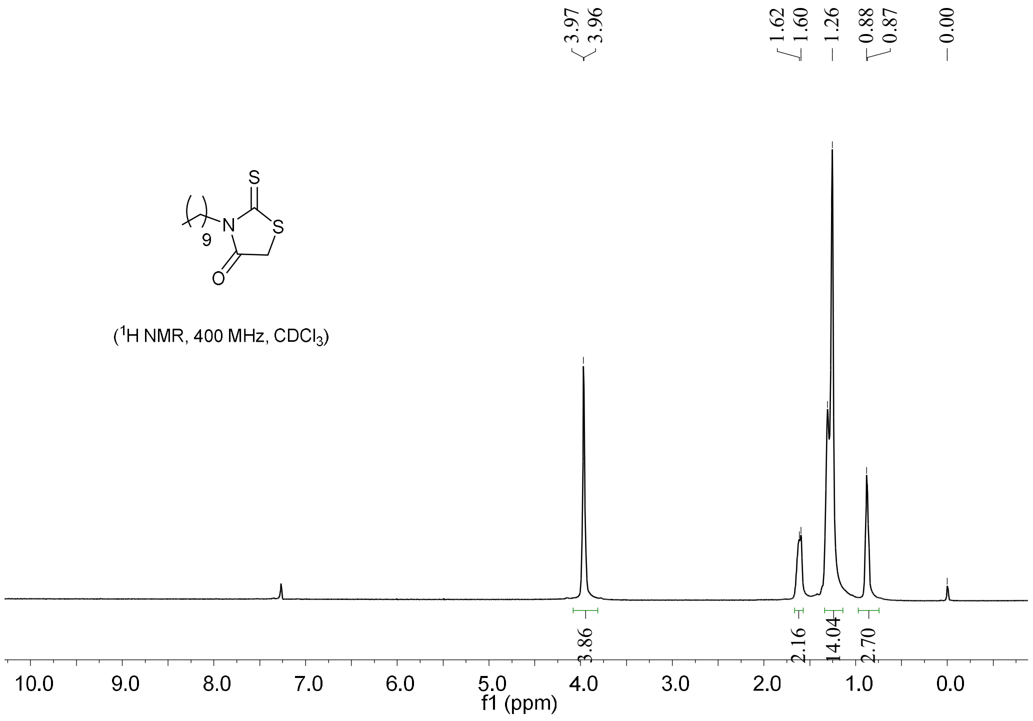

^{1}H NMR of compound **4aa**

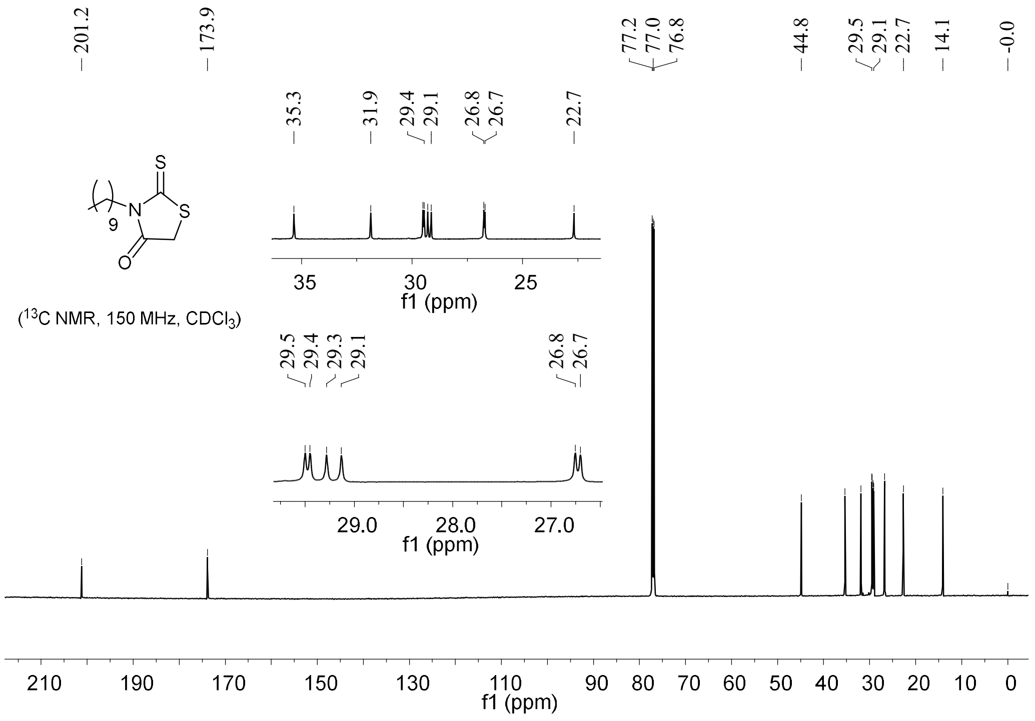

^{13}C NMR of compound **4aa**

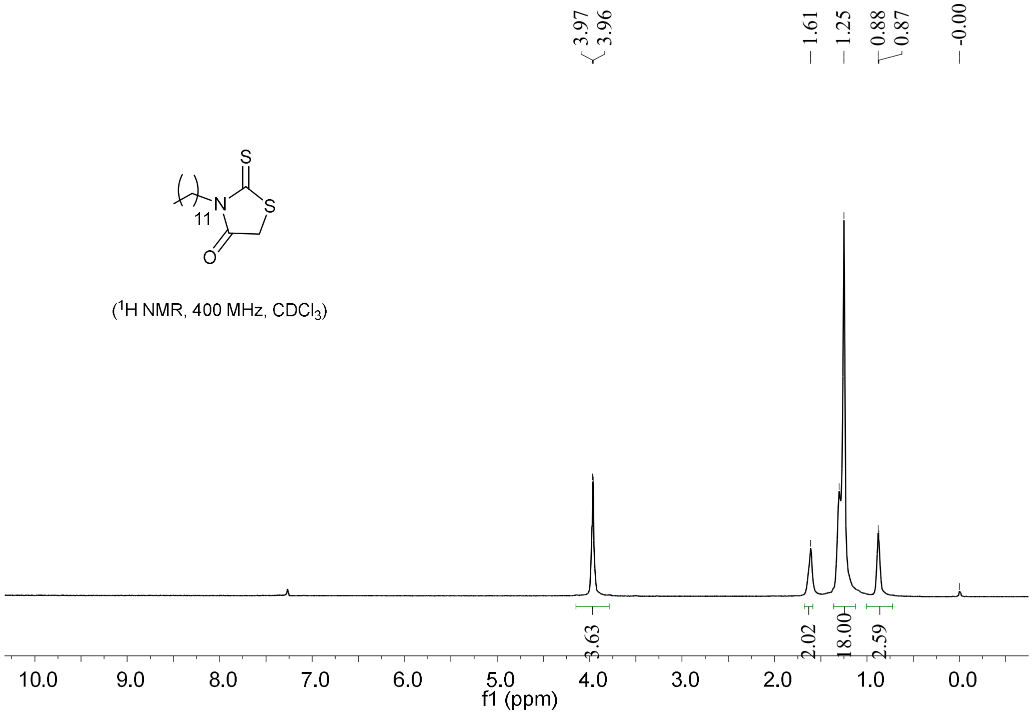

^{1}H NMR of compound **4ab**

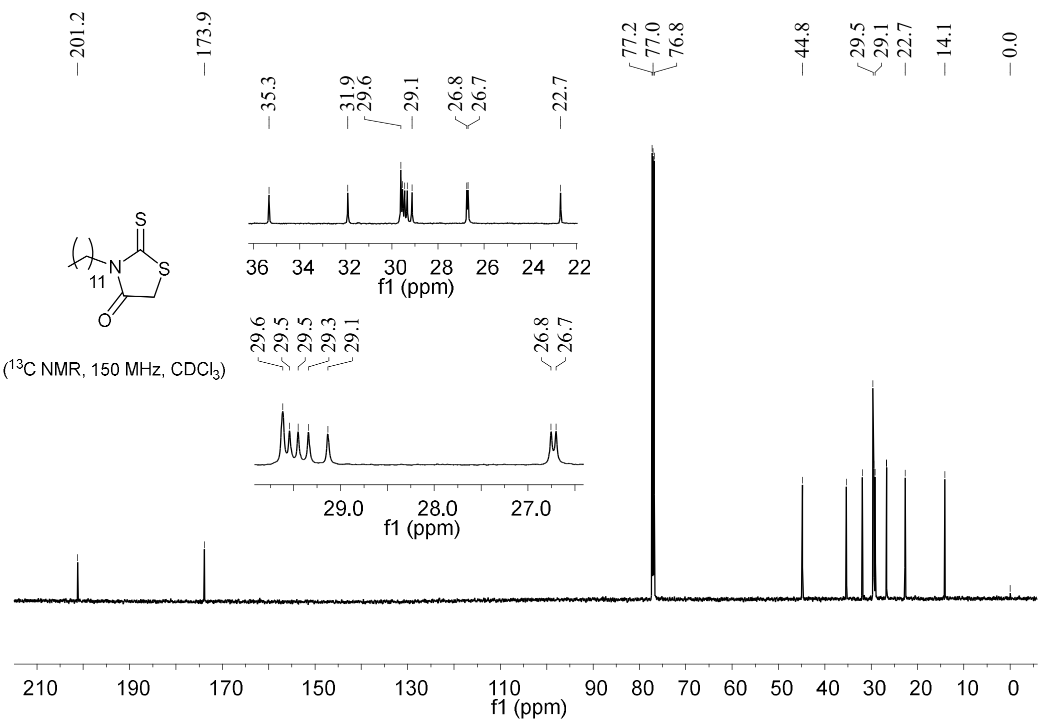

^{13}C NMR of compound **4ab**

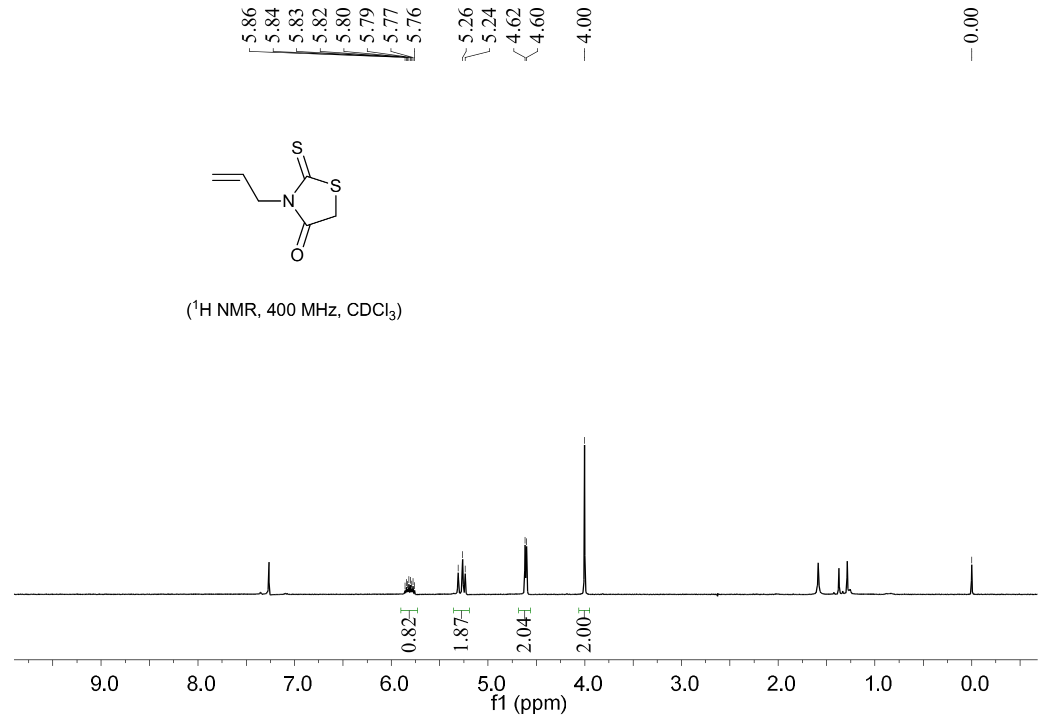

^{1}H NMR of compound **4ac**

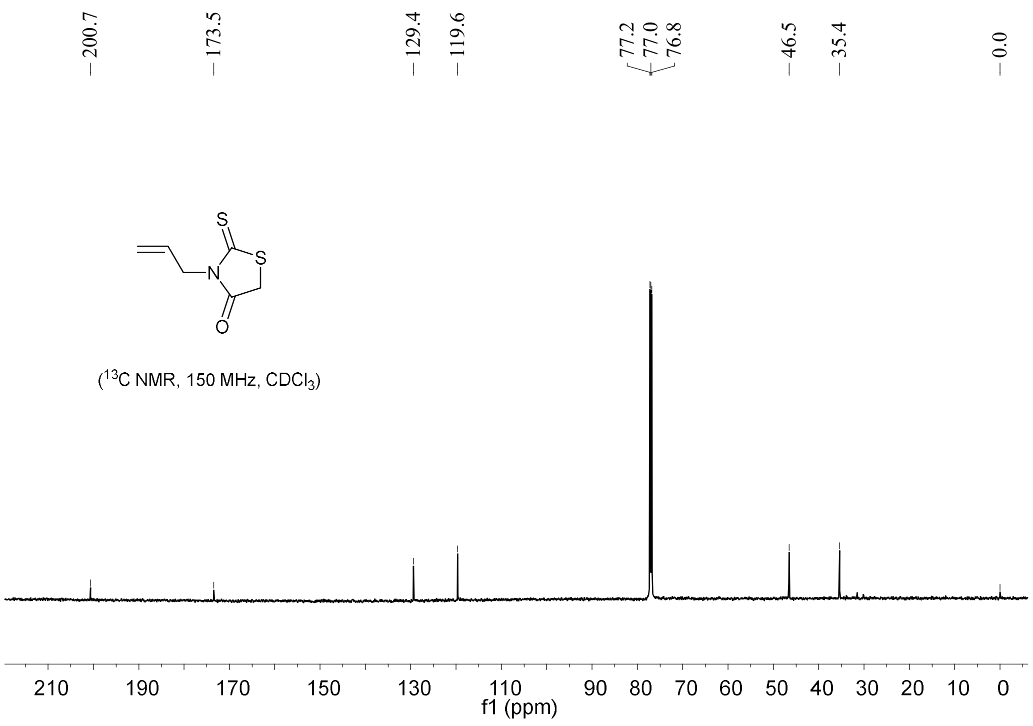

^{13}C NMR of compound **4ac**

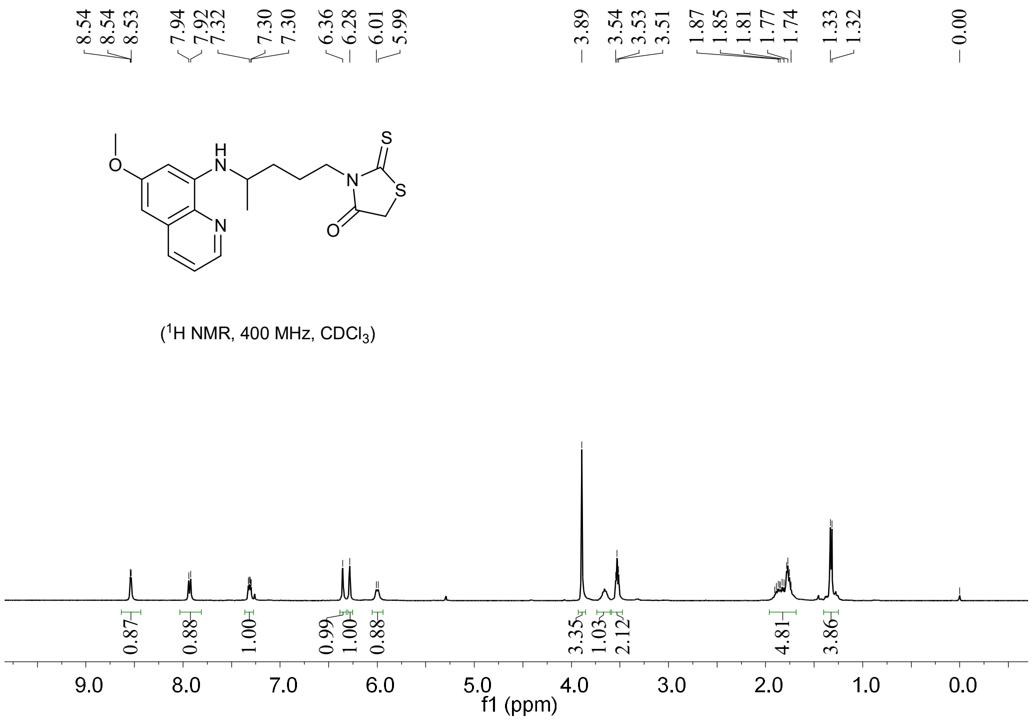

^{1}H NMR of compound **4ad**

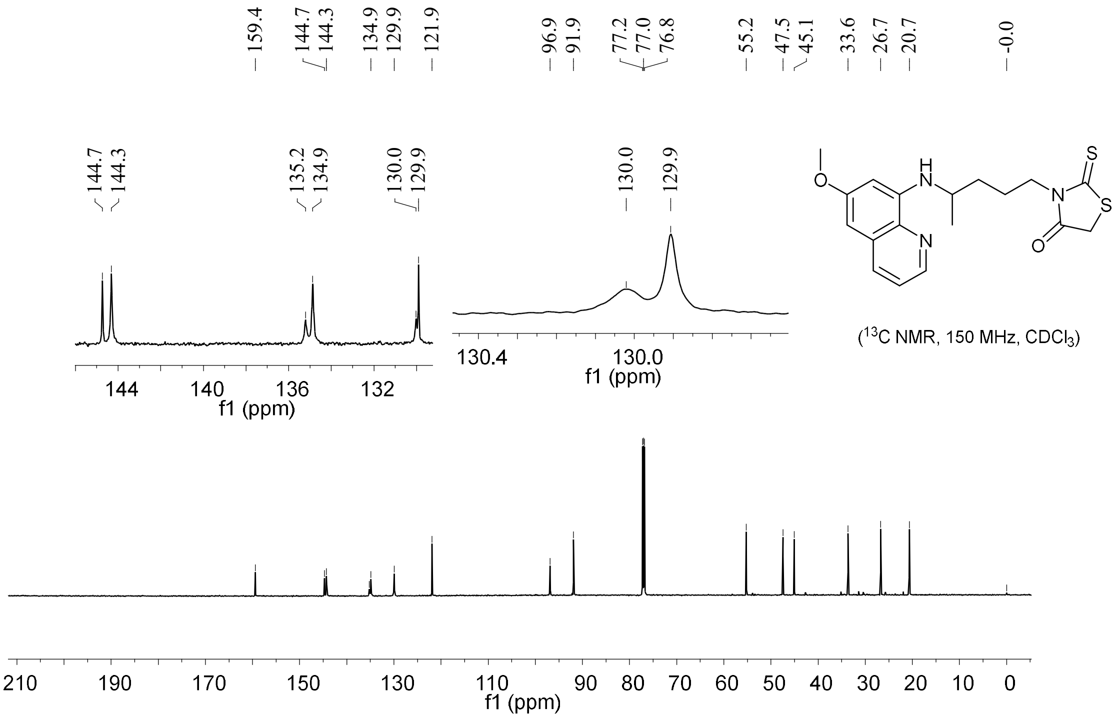

^{13}C NMR of compound **4ad**

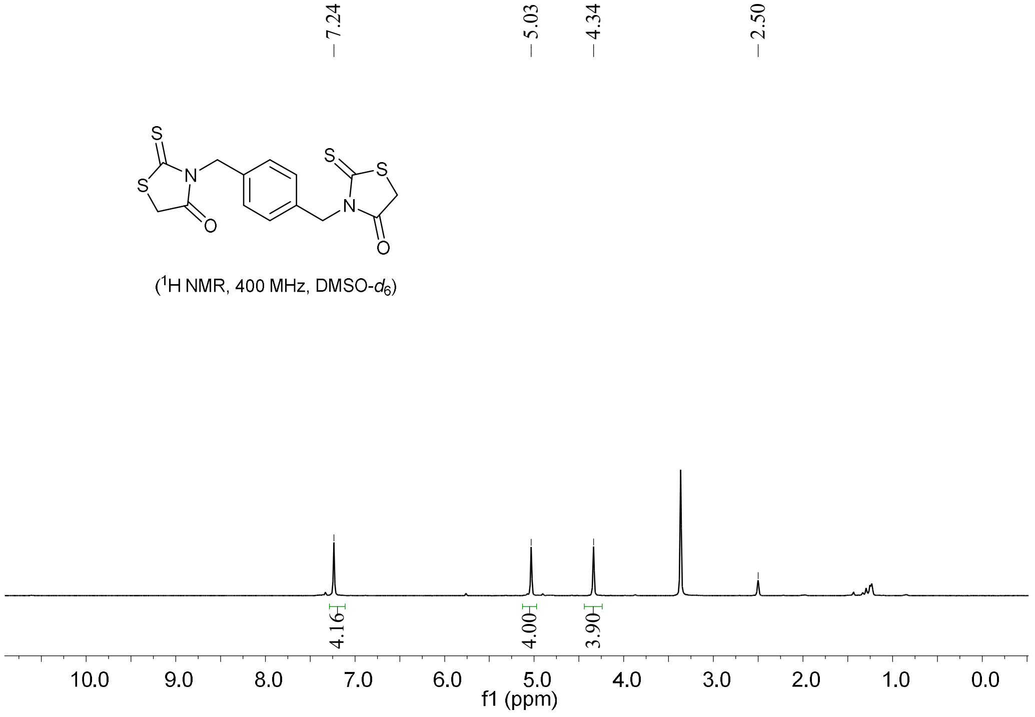

^{1}H NMR of compound **4ae**

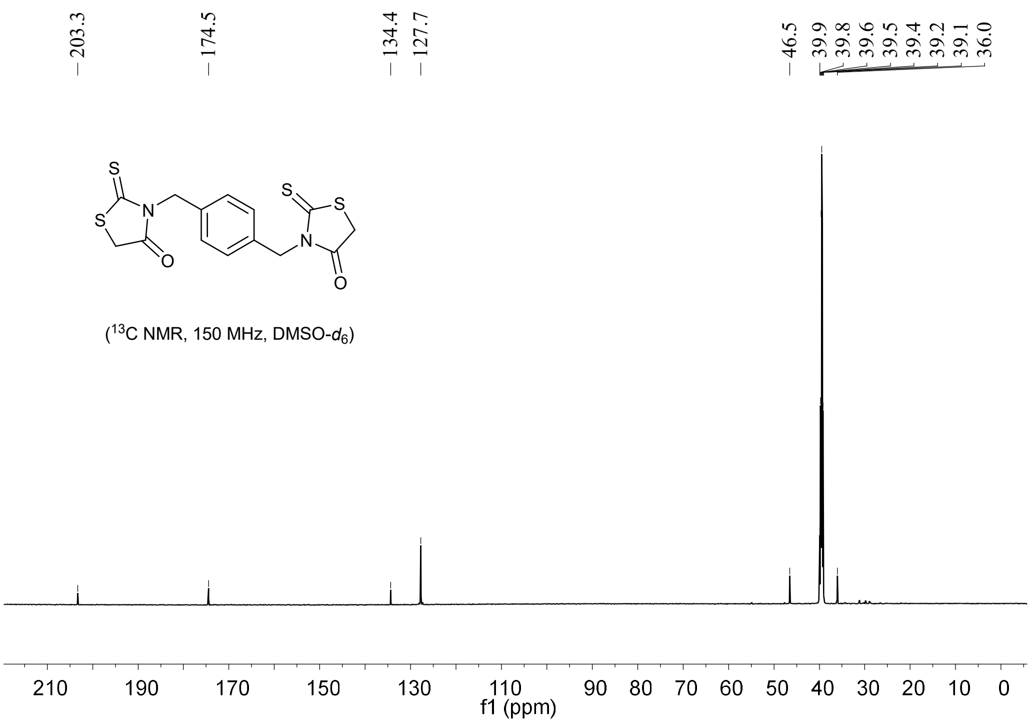

^{13}C NMR of compound **4ae**

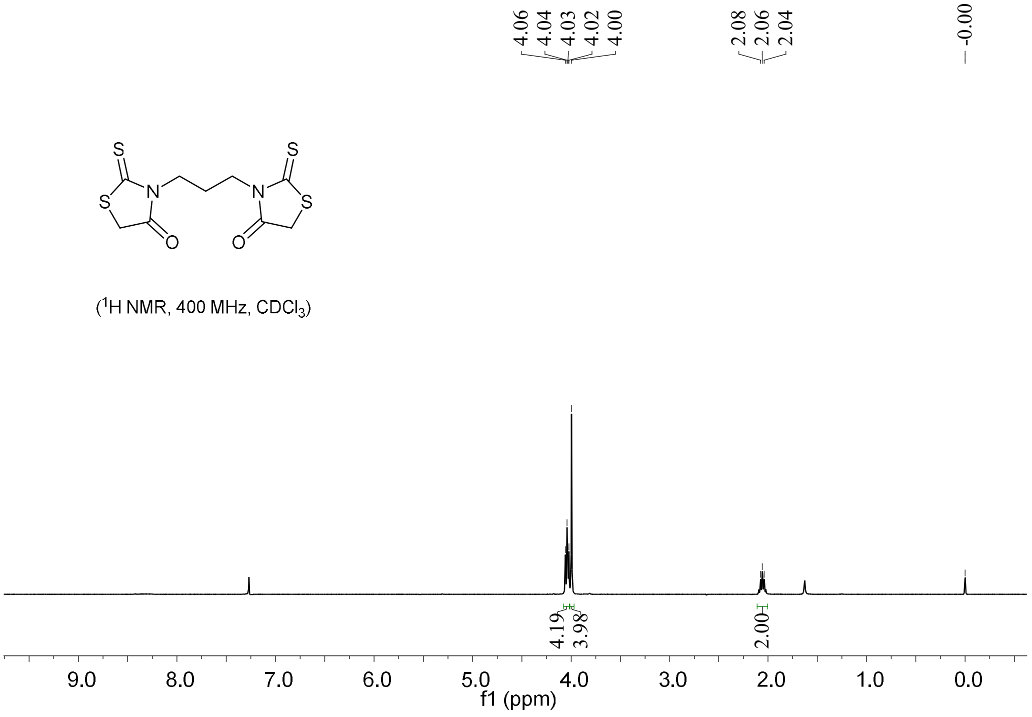

^{1}H NMR of compound **4af**

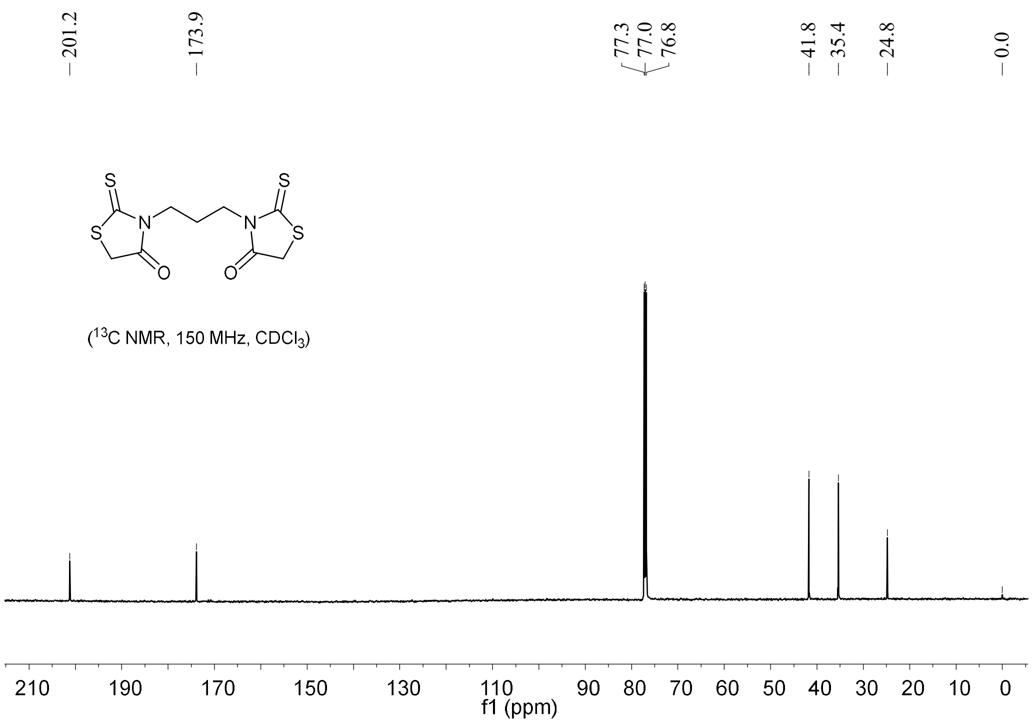

^{13}C NMR of compound **4af**

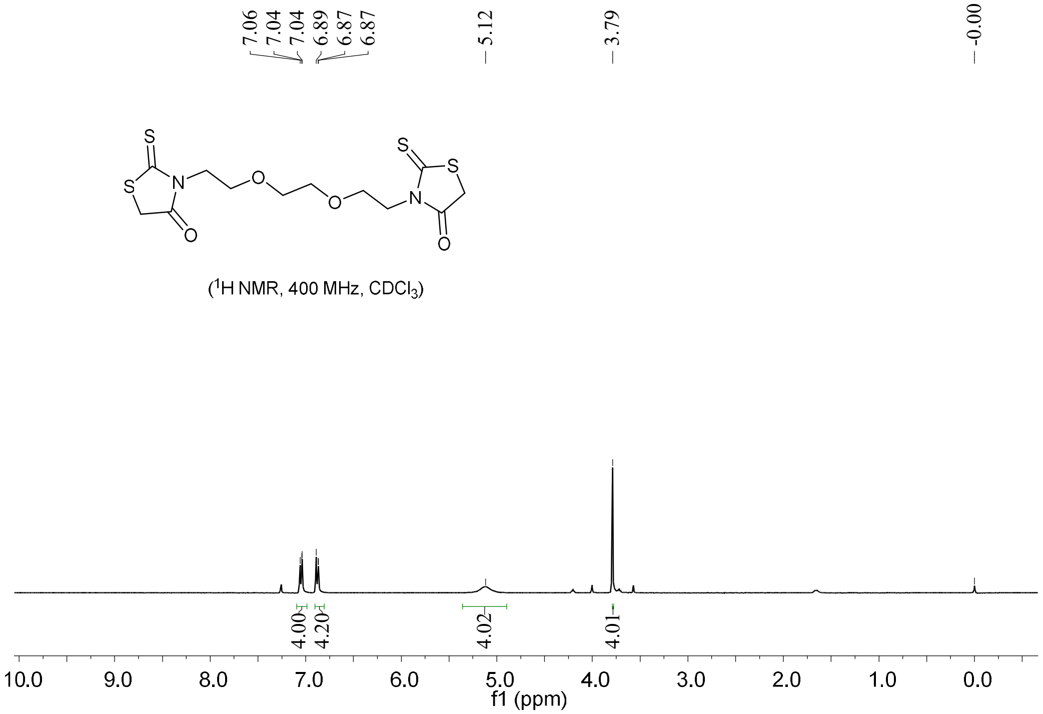

^{1}H NMR of compound **4ag**

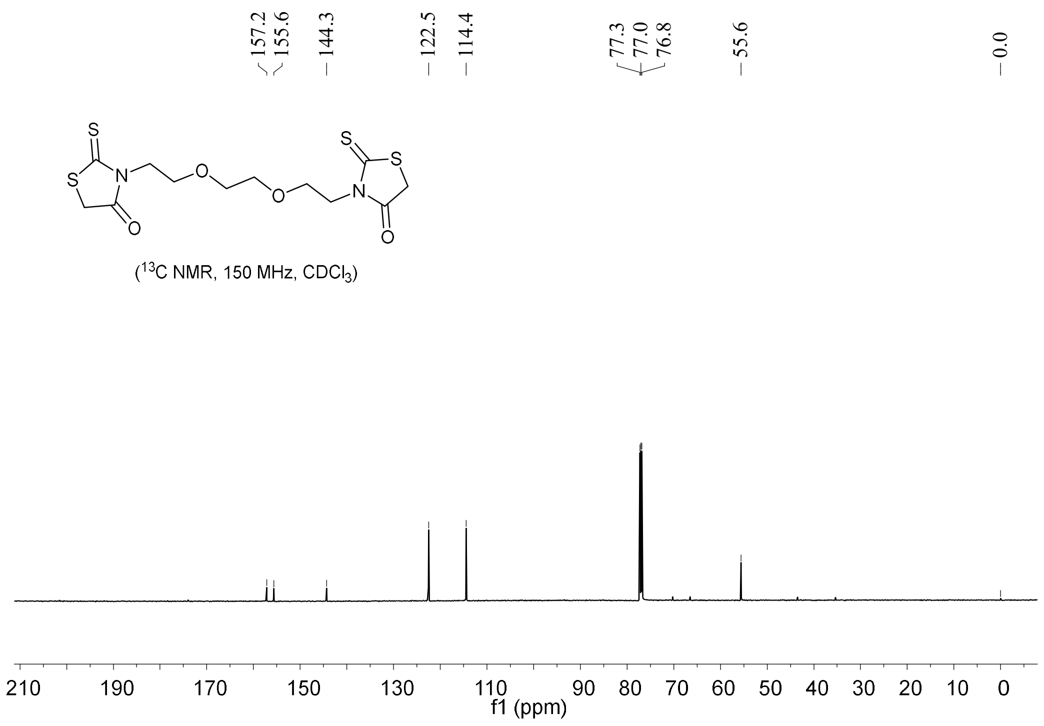

^{13}C NMR of compound **4ag**

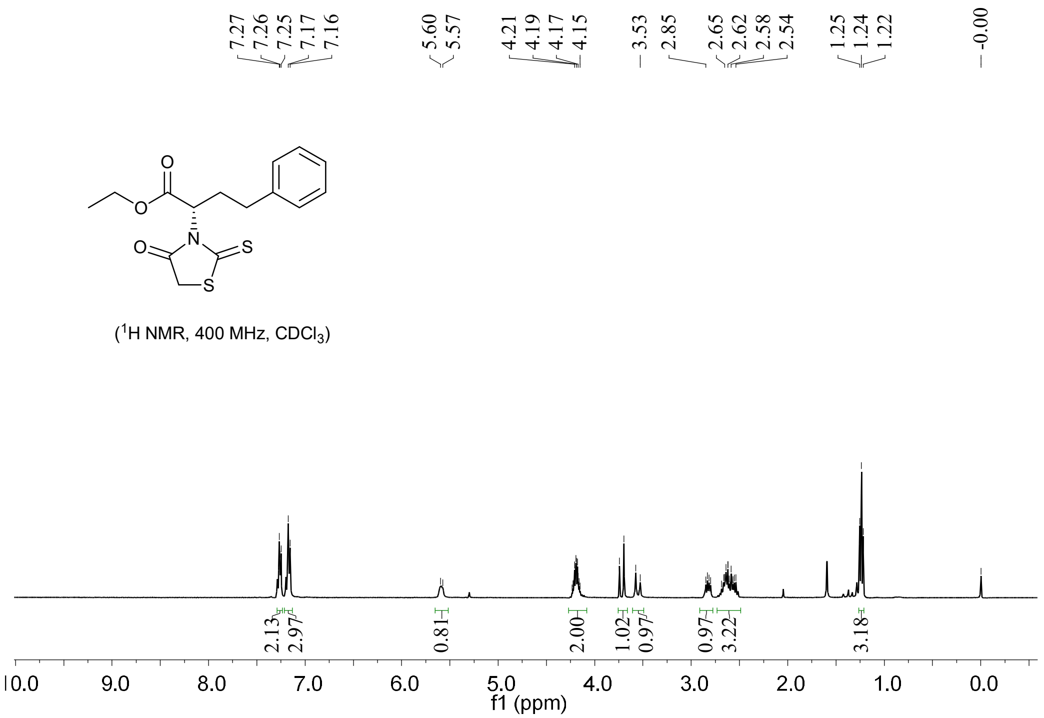

^{1}H NMR of compound **4ah**

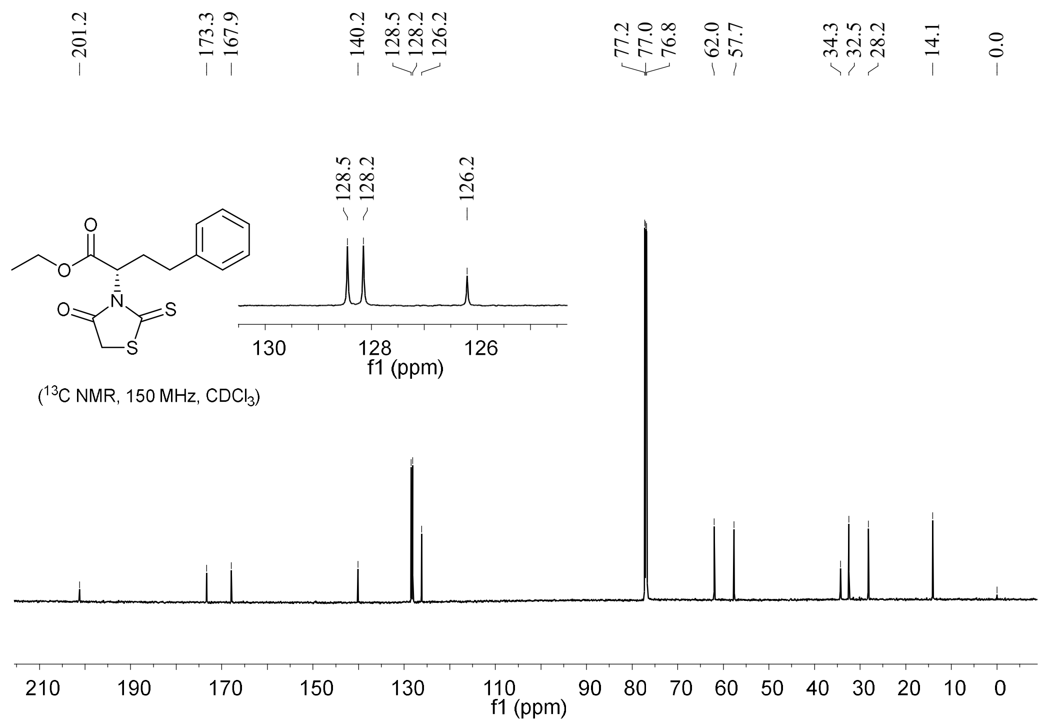

^{13}C NMR of compound **4ah**

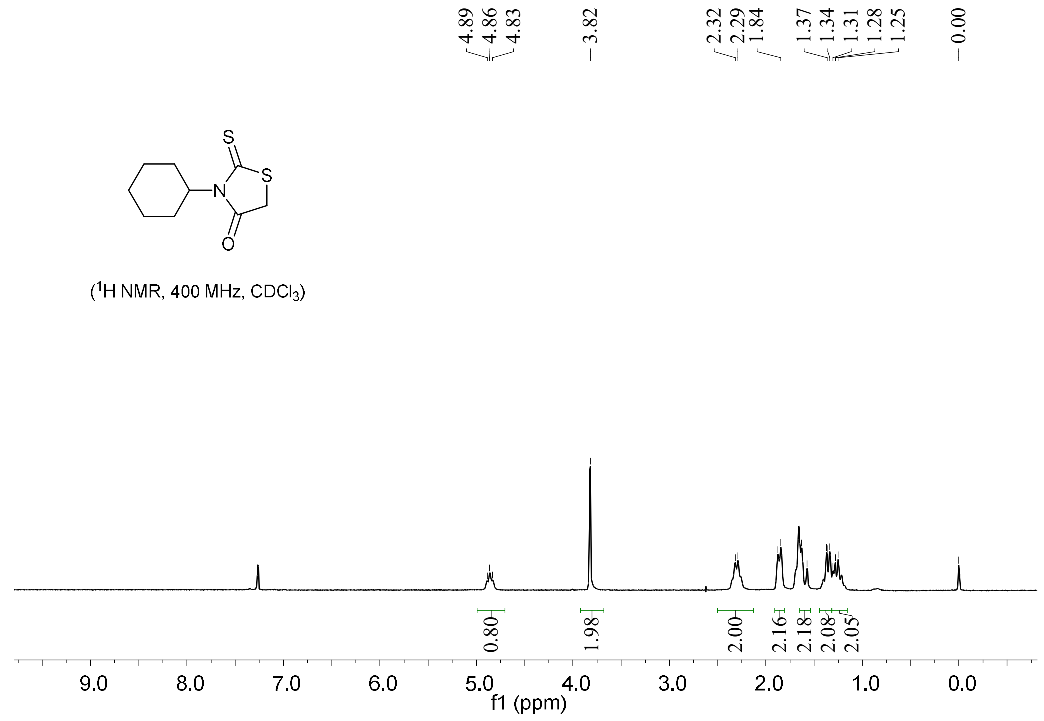

^{1}H NMR of compound **4ai**

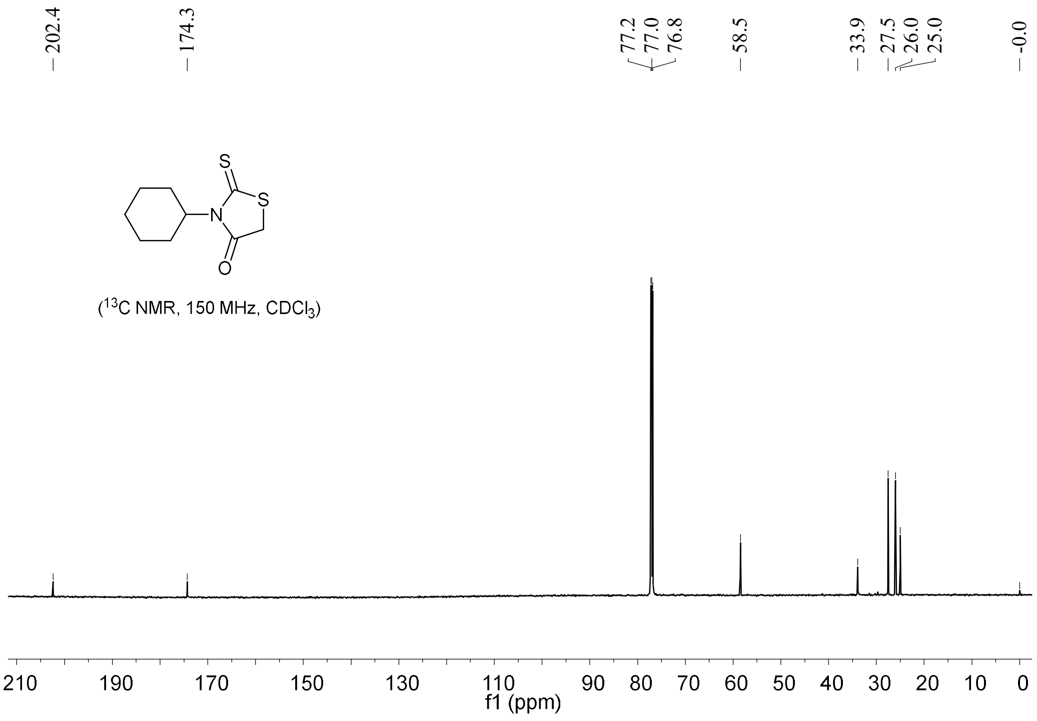

^{13}C NMR of compound **4ai**

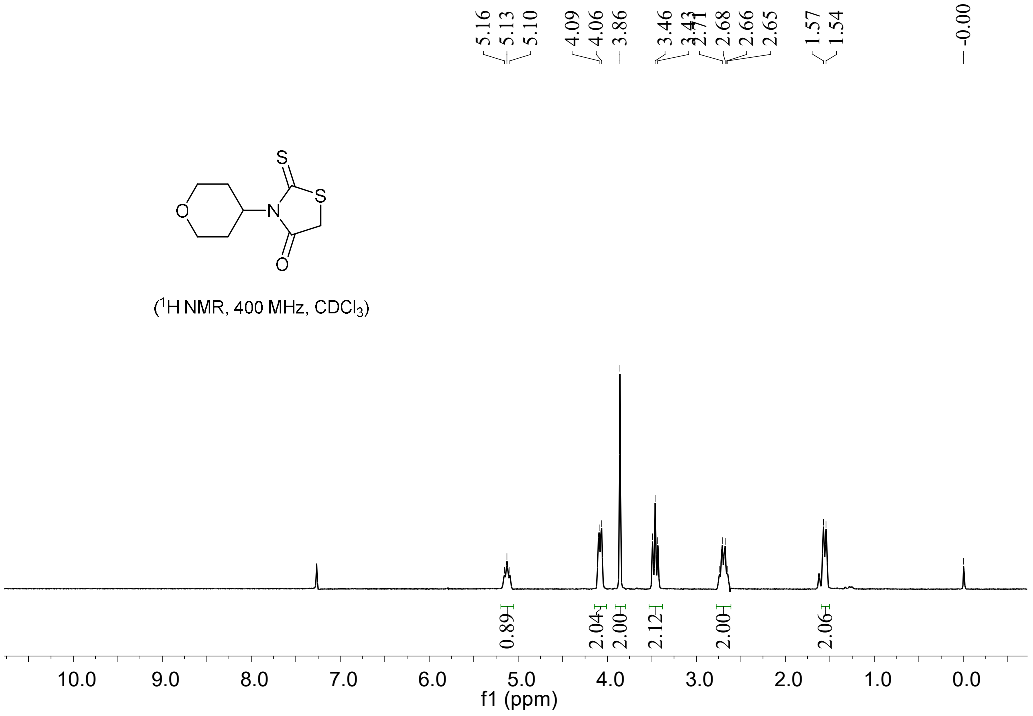

^{1}H NMR of compound **4aj**

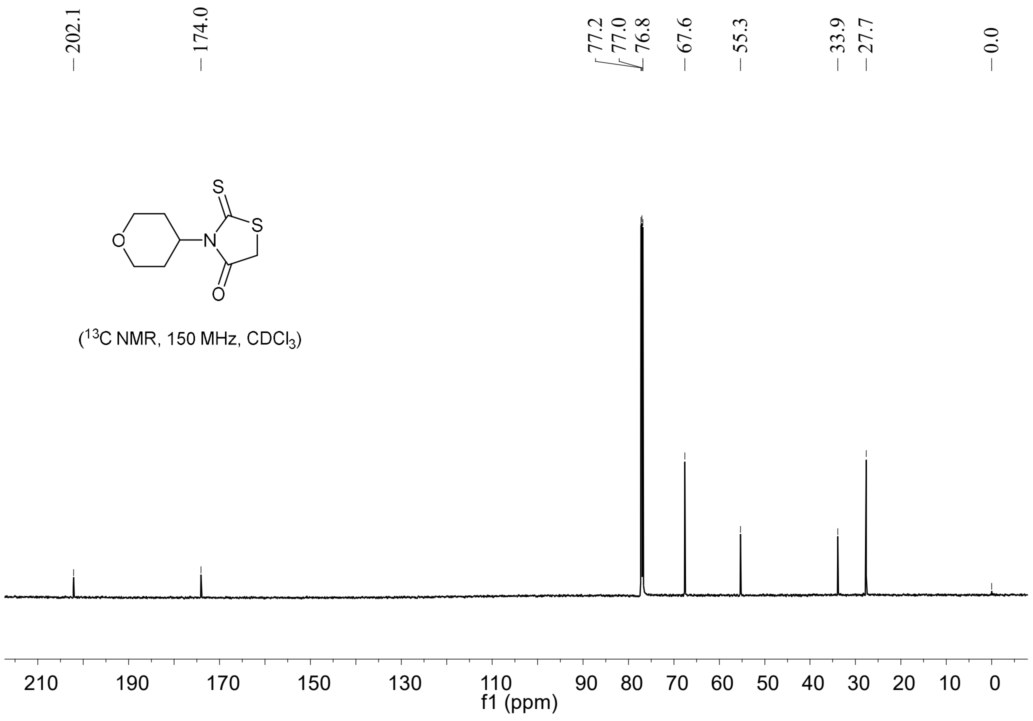

^{13}C NMR of compound **4aj**

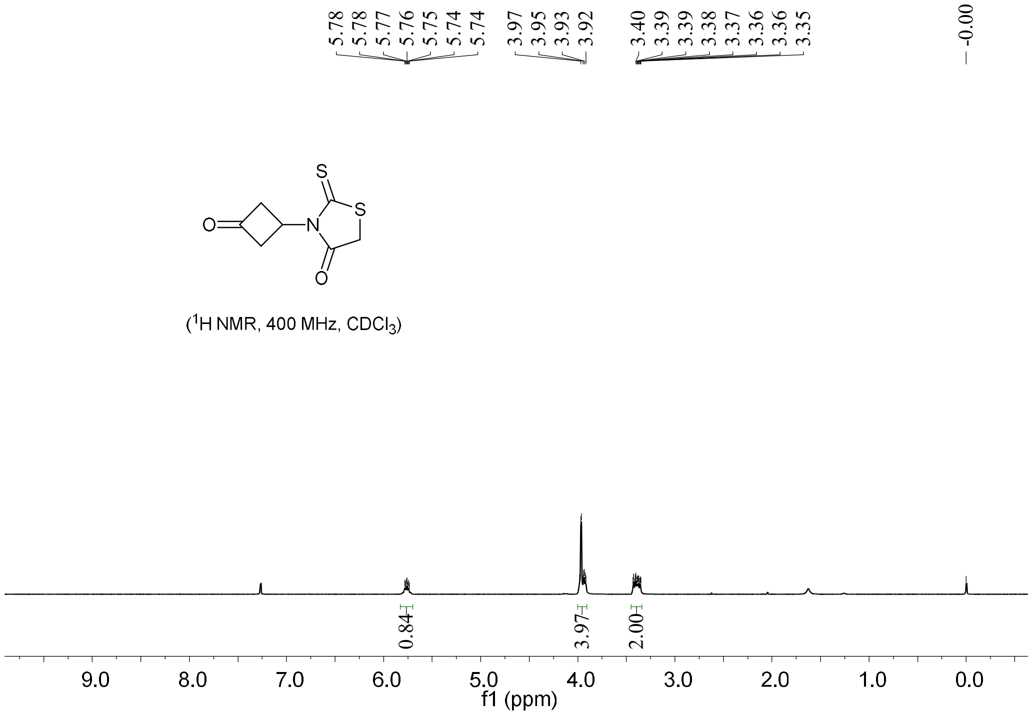

^{1}H NMR of compound **4ak**

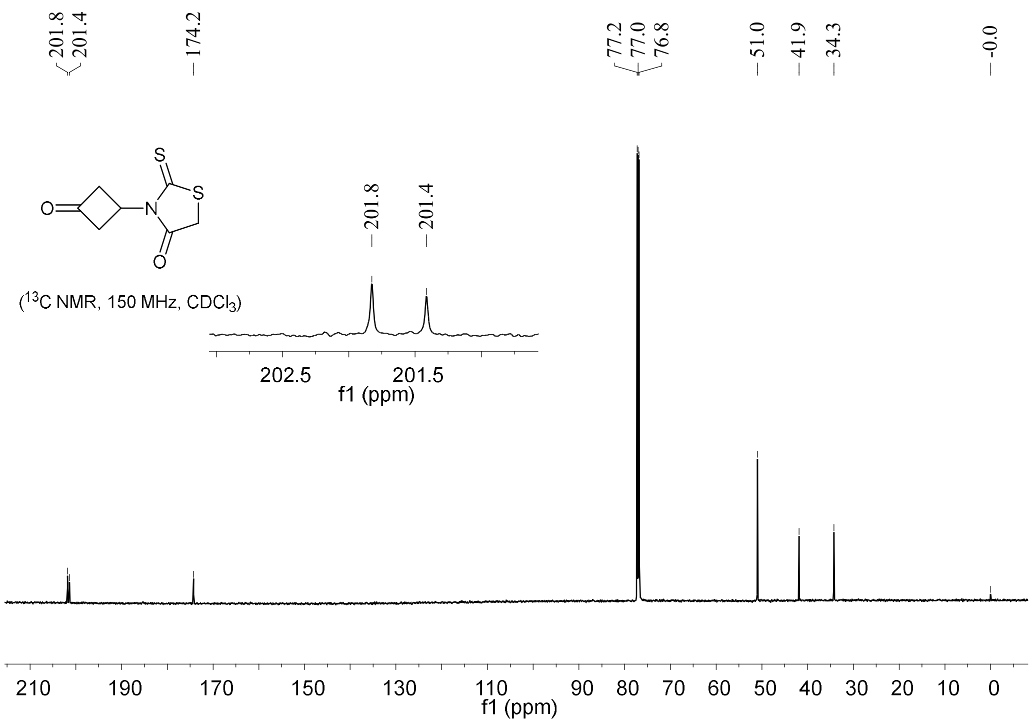

^{13}C NMR of compound **4ak**

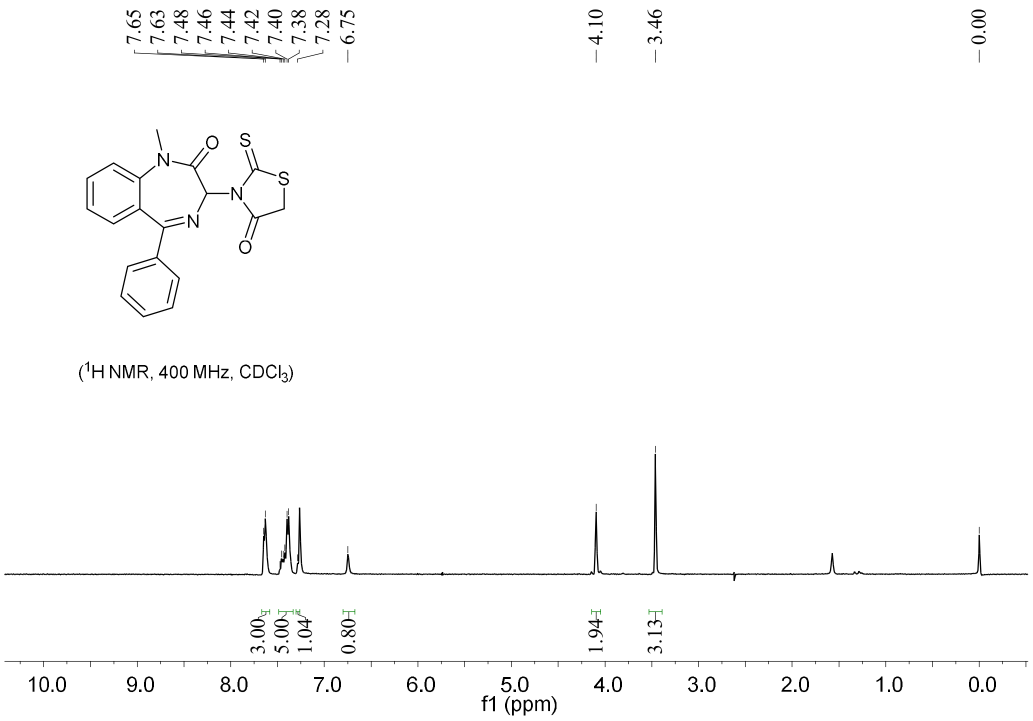

^{1}H NMR of compound **4al**

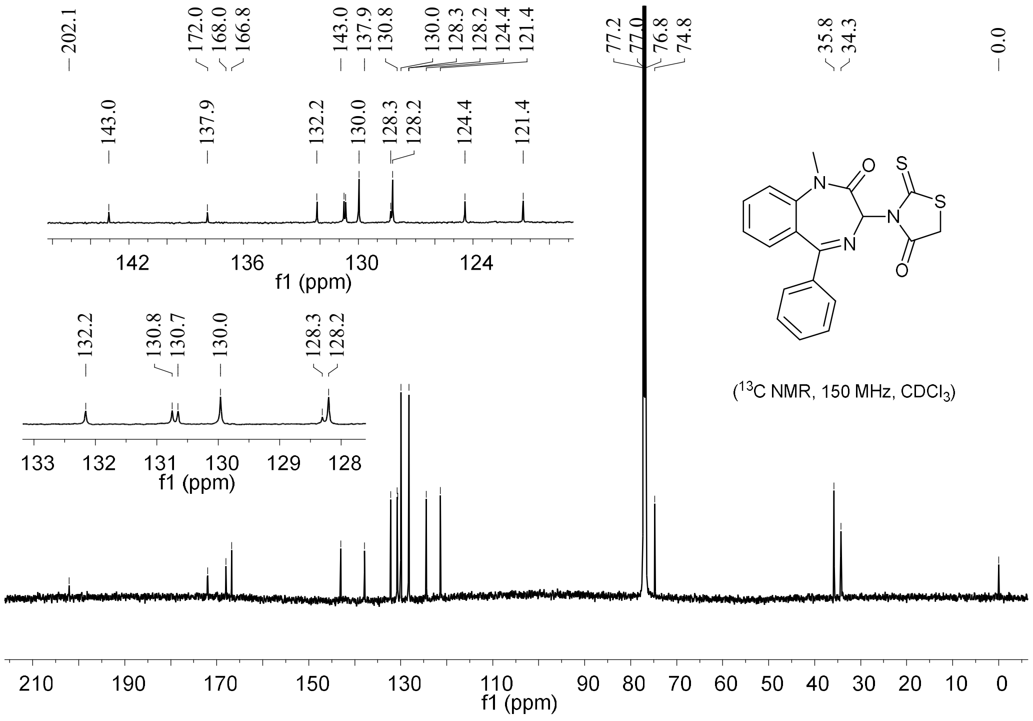

^{13}C NMR of compound **4al**

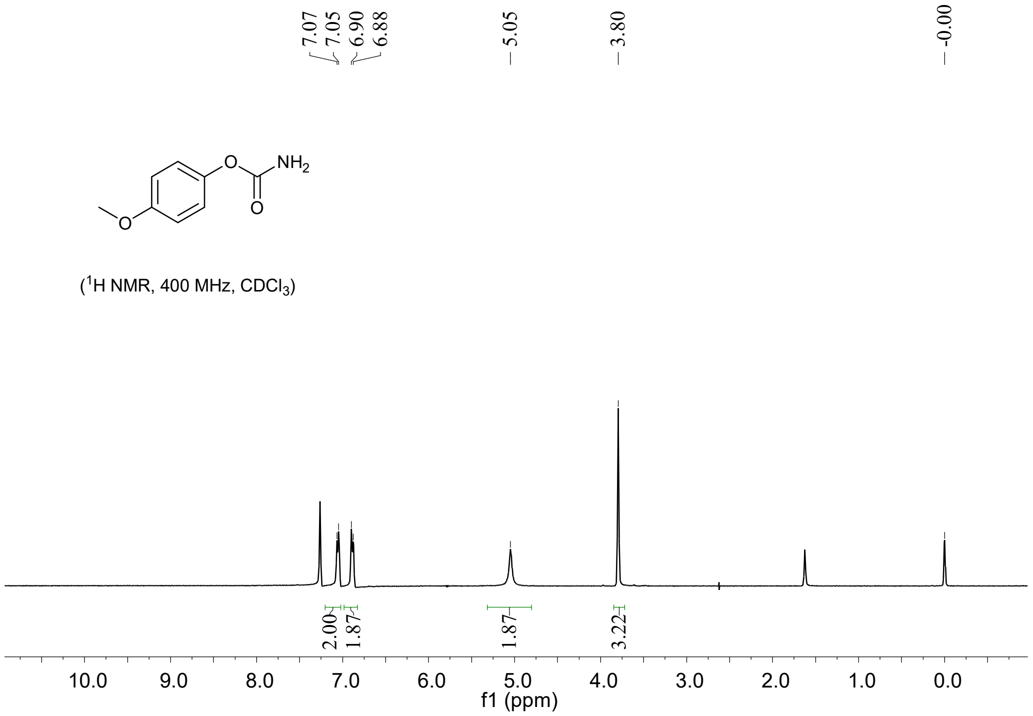

^{1}H NMR of compound **5a**

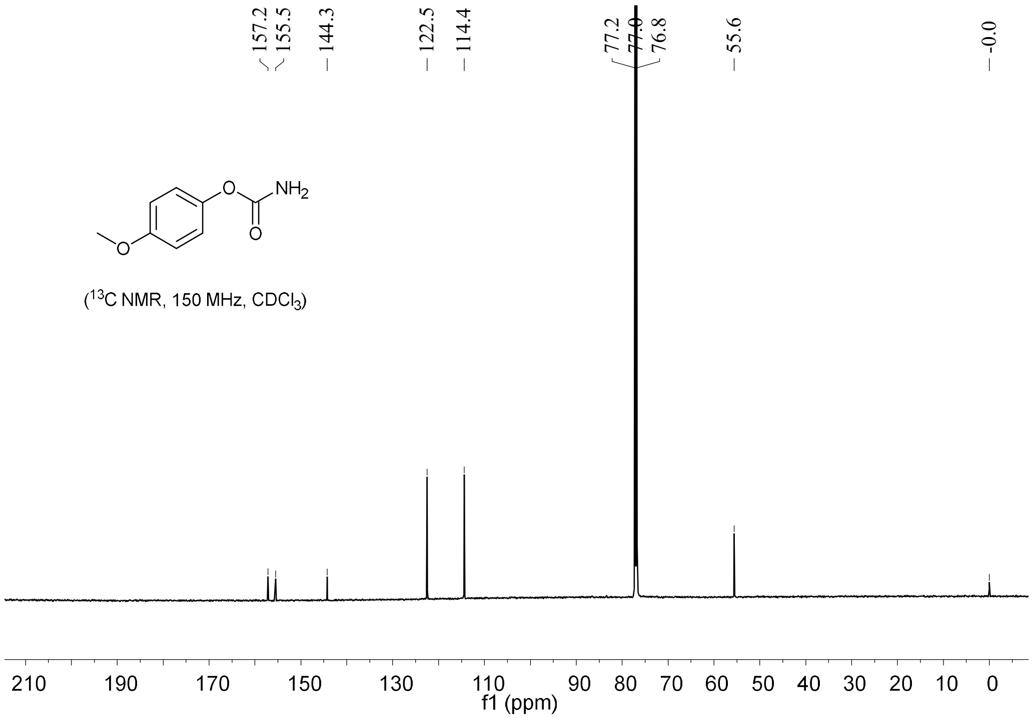

^{13}C NMR of compound **5a**

2 X-ray Crystallography of Compounds **4af**, **4al** and **5a**

3,3'-(Propane-1,3-diyl)bis(2-thioxothiazolidin-4-one) (4af, CCDC 1970414), obtained by recrystallization from MeOH

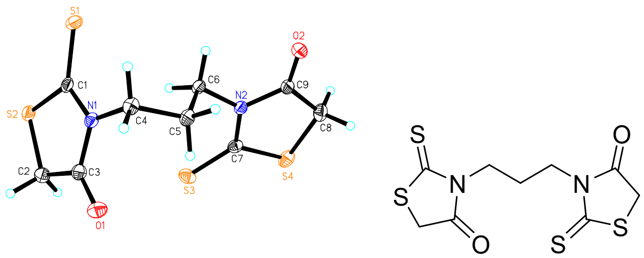

Table 1. Crystal data and structure refinement for **4af**

Identification code	**4af**
Empirical formula	$C_9H_{10}N_2O_2S_4$
Formula weight	306.43
Temperature	167(2) K
Wavelength	1.54178 Å
Crystal system	Monoclinic
space group	P 2(1)/c

Unit cell dimensions	a = 10.2877(7) Å α= 90°.<br>b = 8.9950(6) Å β = 106.999(2)°.<br>c = 14.3510(10) Å γ= 90°.
Volume	1269.99(15) $Å^3$
Z	4
Calculated density	1.603 Mg/m^3
Absorption coefficient	6.819 mm^{-1}
F(000)	632
Crystal size	0.100 x 0.020 x 0.010 mm^3
Theta range for data collection	4.494 to 74.769°
Limiting indices	-12<=h<=12, -11<=k<=10, -17<=l<=17
Reflections collected	10816
Independent reflections	2583 [R(int) = 0.0302]
Completeness to theta = 67.679°	99.7 %
Absorption correction	None
Max. and min. transmission	0.7531 and 0.5475
Refinement method	Full-matrix least-squares on F^2
Data / restraints / parameters	2583 / 0 / 154
Goodness-of-fit on F^2	1.063
Final R indices [I>2sigma(I)]	R1 = 0.0252, wR2 = 0.0657
R indices (all data)	R1 = 0.0260, wR2 = 0.0663
Extinction coefficient	n/a
Largest diff. peak and hole	0.283 and -0.243 e.$Å^{-3}$

3-(1-Methyl-2-oxo-5-phenyl-2,3-dihydro-1H-benzo[e][1,4]diazepin-3-yl)-2-thioxothiazolidin-4-one (4al, CCDC 1970415), obtained by recrystallization from MeOH

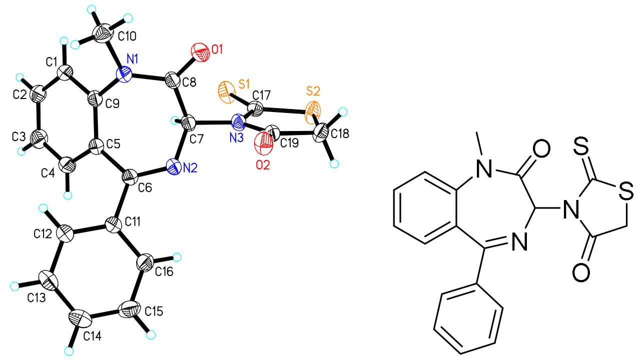

Table 2. Crystal data and structure refinement for **4al**

Identification code	**4al**
Empirical formula	$C_{19}H_{15}N_3O_2S_2$
Formula weight	381.46

Temperature	296(2) K
Wavelength	1.54178 Å
Crystal system	Monoclinic
space group	P 21/c
Unit cell dimensions	a = 8.8074(12) Å α= 90°.<br>b = 19.277(3) Å β = 101.942(16)°.<br>c = 10.540(2) Å γ= 90°.
Volume	1750.7(5) $Å^3$
Z	4
Calculated density	1.447 Mg/m^3
Absorption coefficient	2.921 mm^{-1}
F(000)	792
Crystal size	0.18 x 0.10 x 0.08 mm^3
Theta range for data collection	4.59 to 68.70°
Limiting indices	-10<=h<=9, -22<=k<=23, -12<=l<=12
Reflections collected	12010
Independent reflections	3166 [R(int) = 0.0425]
Completeness to theta = 68.70°	97.5 %
Absorption correction	Semi-empirical from equivalents
Max. and min. transmission	0.7531 and 0.5475
Refinement method	Full-matrix least-squares on F^2
Data / restraints / parameters	3166 / 0 / 236
Goodness-of-fit on F^2	1.096
Final R indices [I>2sigma(I)]	R1 = 0.0721, wR2 = 0.1925
R indices (all data)	R1 = 0.0911, wR2 = 0.2284
Extinction coefficient	0.030(3)
Largest diff. peak and hole	0.790 and -0.641 e.$Å^{-3}$

4-Methoxyphenyl carbamate (5a, CCDC 1970413), obtained by recrystallization from MeOH

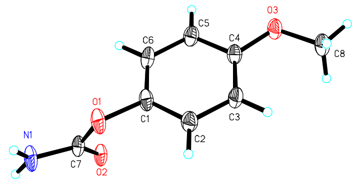

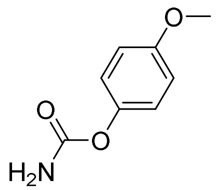

Table 3. Crystal data and structure refinement for **5a**

Identification code	**5a**
Empirical formula	$C_8H_9NO_3$
Formula weight	167.16
Temperature	167(2) K

Wavelength	1.54178 Å
Crystal system	Monoclinic
space group	P 2(1)/c
Unit cell dimensions	a = 15.2088(13) Å α= 90°.<br>b = 5.5527(5) Å β = 90.269(6)°.<br>c = 9.9043(8) Å γ= 90°.
Volume	836.41(12) $Å^3$
Z	4
Calculated density	1.327 Mg/m^3
Absorption coefficient	0.866 mm^{-1}
F(000)	352
Crystal size	0.120 x 0.080 x 0.030 mm^3
Theta range for data collection	2.905 to 74.481°
Limiting indices	-18<=h<=19, -6<=k<=5, -5<=l<=12
Reflections collected	6602
Independent reflections	1699 [R(int) = 0.1213]
Completeness to theta = 67.679°	99.9 %
Absorption correction	Semi-empirical from equivalents
Max. and min. transmission	0.97 and 0.65
Refinement method	Full-matrix least-squares on F^2
Data / restraints / parameters	1699 / 0 / 109
Goodness-of-fit on F^2	1.138
Final R indices [I>2sigma(I)]	R1 = 0.0755, wR2 = 0.2384
R indices (all data)	R1 = 0.0849, wR2 = 0.2530
Extinction coefficient	n/a
Largest diff. peak and hole	0.385 and -0.369 e.$Å^{-3}$
